# Plant Copper Metalloenzymes As Prospects for New Metabolism Involving Aromatic Compounds

**DOI:** 10.3389/fpls.2021.692108

**Published:** 2021-11-29

**Authors:** Lisa S. Mydy, Desnor N. Chigumba, Roland D. Kersten

**Affiliations:** Department of Medicinal Chemistry, University of Michigan, Ann Arbor, MI, United States

**Keywords:** copper enzyme, plant metabolism, biosynthesis, copper, biocatalysis

## Abstract

Copper is an important transition metal cofactor in plant metabolism, which enables diverse biocatalysis in aerobic environments. Multiple classes of plant metalloenzymes evolved and underwent genetic expansions during the evolution of terrestrial plants and, to date, several representatives of these copper enzyme classes have characterized mechanisms. In this review, we give an updated overview of chemistry, structure, mechanism, function and phylogenetic distribution of plant copper metalloenzymes with an emphasis on biosynthesis of aromatic compounds such as phenylpropanoids (lignin, lignan, flavonoids) and cyclic peptides with macrocyclizations via aromatic amino acids. We also review a recent addition to plant copper enzymology in a copper-dependent peptide cyclase called the BURP domain. Given growing plant genetic resources, a large pool of copper biocatalysts remains to be characterized from plants as plant genomes contain on average more than 70 copper enzyme genes. A major challenge in characterization of copper biocatalysts from plant genomes is the identification of endogenous substrates and catalyzed reactions. We highlight some recent and future trends in filling these knowledge gaps in plant metabolism and the potential for genomic discovery of copper-based enzymology from plants.

## Introduction

Copper is an essential trace metal for plants that is required for control of the cellular redox state and electron transport reactions in oxidative phosphorylation and photosynthesis. It is also an important cofactor for metabolic reactions in lignin biosynthesis during cell wall formation and in biosynthesis of alkaloids, flavonoids, lignans and cyclic peptides (Barros et al., 2015; Chigumba et al., 2021). Copper is a redox-active transition metal and generally exists in two oxidation states, Cu(I) and Cu(II), under plant physiological conditions. Before the evolution of photosynthetic organisms and the oxygenation of the atmosphere, copper was mainly bound as insoluble copper sulfide [Cu(I)], which was less accessible to metabolism of early life forms. Life during this time period is hypothesized to have evolved mostly iron-based biocatalysts due to the broad electron potential of Fe(III)/Fe(II) (−0.5 to 0.6 eV) and solubility of Fe(II) under the anaerobic conditions that characterized the early Earth (Crichton and Pierre, 2001). The emergence of photosynthetic cyanobacteria on Earth eventually led to an increase in atmospheric oxygen about 2.2–2.4 billion years ago (Luo, 2016; Gumsley et al., 2017; Poulton et al., 2021) and, consequently, to increased copper bioavailability by oxidation of Cu(I) to soluble Cu(II) (Crichton and Pierre, 2001). This, together with a redox potential in the range of 0 to 0.8 eV for Cu(II)/Cu(I), likely made copper a metal cofactor alternative for oxidative biocatalysis in an aerobic atmosphere, while iron remained a dominant metal cofactor in metabolism due to its broad electron potential and ability to oxidize unactivated substrates (Kaim and Rall, 1996; Crichton and Pierre, 2001). As oxygen-generating organisms, plants have expanded their use of copper in metabolism due to its versatility in catalyzing metabolic reactions in an aerobic environment and its ability to reduce dioxygen. Copper is a cofactor for several classes of metalloenzymes that expanded in plant genomes after their transition to land (Weng and Chapple, 2010). Copper enzymes often have iron-based counterparts in plant metabolism catalyzing similar reactions, based on the ability of copper- and iron-proteins to participate in similar biological reactions (Bertini et al., 1994). Parallel metabolic routes utilizing either copper or iron are hypothesized to enable plants to respond better to temporary copper or iron nutrient shortages (Merchant et al., 2006; Burkhead et al., 2009). This review focuses on the currently known catalyzed reactions, enzymatic mechanisms, functions, and phylogenetic distributions of copper metalloenzymes in plant metabolism. For in depth discussion of bioinorganic chemistry of copper and plant copper homeostasis, we refer to excellent reviews by Burkhead et al. (2009), Messerschmidt (2010), Solomon et al. (2014), and Printz et al. (2016), respectively. This review does not cover cytochrome c oxidase, which is an essential copper-containing protein in oxidative phosphorylation and, therefore, plant energy metabolism. For insights into structure and function of this protein complex in terms of copper biochemistry, we also recommend the reviews by Messerschmidt (2010) and Solomon et al. (2014).

## Copper-Catalyzed Reactions in Plant Metabolism

**FIGURE 1:**
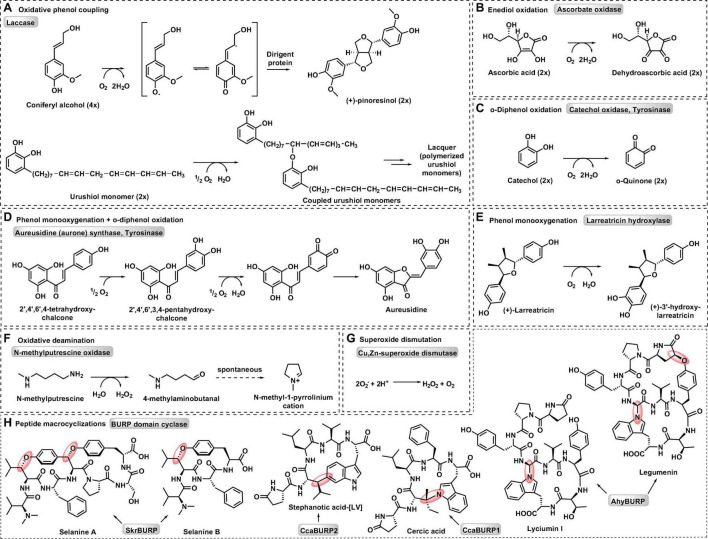
Representative metabolic reactions catalyzed by plant copper metalloenzymes. **(A)** Oxidative phenol coupling by laccase. **(B)** Enediol oxidation by ascorbate oxidase. **(C)** *o*-Diphenol oxidation by catechol oxidase and tyrosinase. **(D)** Phenol monooxygenation and *o*-diphenol oxidation by aurone synthase and tyrosinase. **(E)** Phenol monooxygenation by larreatricin hydroxylase. **(F)** Oxidative deamination by N-methylputrescine oxidase. **(G)** Superoxide dismutation by Cu, Zn-superoxide dismutase. **(H)** Peptide macrocyclizations by BURP domain cyclase.

Six classes of copper metalloenzymes involved in plant metabolism are reviewed here: laccase, ascorbate oxidase, type III polyphenol oxidases, copper-dependent amine oxidase, and Cu,Zn-superoxide-dismutase. Metabolic reactions catalyzed by these enzymes are diverse oxidative transformations that use dioxygen as a general oxidant (Figure 1; Solomon et al., 2014). In addition, we review a recent addition to plant copper enzymes in BURP domain peptide cyclases (Chigumba et al., 2021), which have yet to be characterized in their use of dioxygen. Laccases catalyze one-electron oxidations of monophenolic substrates in order to generate phenoxy radicals, which subsequently react with each other to form neolignans, such as (+)-pinoresinol via stereoselective guidance by non-catalytic dirigent proteins (Davin et al., 1997), or lignin and urushiol polymers via oxidative coupling (Barros et al., 2015; Figure 1A). Similar to laccases, ascorbate oxidases mediate one-electron oxidation of ascorbate to semidehydroascorbate radicals, which further dismutate to ascorbate and dehydroascorbate (Figure 1B) resulting in ascorbate enediol oxidation. Type III polyphenol oxidases (PPO) such as catechol oxidases, tyrosinases and aurone synthases catalyze enediol oxidations by two-electron oxidations of *o*-diphenol substrates to *o*-quinones (Figure 1B). Tyrosinases and aurone synthases also catalyze a preceding monooxygenation of monophenol substrates to *o*-diphenols before *o*-quinone formation. Some type III PPOs only catalyze this monooxygenation of monophenols. An example for this is the enantiospecific 3′-hydroxylation of (+)-larreatricin in 8–8′-lignan biosynthesis in creosote bush (*Larrea tridentata*) (Cho et al., 2003; Figure 1E). In aurone synthases, the generated chalcone quinone is further cyclized to an aurone flavonoid (Nakayama et al., 2000; Figure 1D). Amine oxidases convert organic amines or polyamines into aldehydes by oxidative deamination. For example, *N*-methylputrescine oxidase catalyzes the formation of 4-methylaminobutanal from *N*-methylputresine, which subsequently cyclizes spontaneously to *N*-methyl-4-pyrrolinium-cation, a building block of tropane alkaloid biosynthesis (Figure 1F; Katoh et al., 2007). Superoxide dismutation to oxygen and hydrogen peroxide is catalyzed by superoxide dismutases (SOD) such as Cu,Zn-SOD (Figure 1G). These enzymes reduce oxidative stress from reactive oxygen species and generate oxygen and hydrogen peroxide for lignin biosynthetic laccases and class III peroxidases, respectively (Barros et al., 2015). Finally, BURP domain peptide cyclases catalyze the formation of chemically diverse crosslinks between tyrosine and tryptophan amino acid side chains and unactivated carbons in other amino acids (Figure 1H; Chigumba et al., 2021). Characterized BURP domain peptide cyclases are autocatalytic enzymes, which are involved in the biosynthesis of plant ribosomally-encoded and posttranslationally-modified peptides (RiPPs) with side-chain-derived macrocyclizations (Chigumba et al., 2021). BURP-domain-derived RiPPs are mono- or bicyclic peptides with C(sp^3^)-C(sp^2^)-, C(sp^3^)-O- and C(sp^3^)-N-crosslinks.

## Mechanistic Basis of Plant Copper Metalloenzymes

Plant copper metalloenzymes are generally classified by their catalyzed reactions, substrate specificity and copper binding site (Figure 1 and Table 1). Laccase, tyrosinases, catechol oxidases and aurone synthases have phenolic substrates and belong to the family of PPO. Similarly, ascorbate oxidase uses ascorbate as a substrate, amine oxidases have organic amines as substrates (Solomon et al., 2014) and BURP domain peptide cyclases have intrinsic core peptide sequences with C-terminal tyrosines and tryptophans as substrates (Chigumba et al., 2021). Importantly, dioxygen serves as either a substrate or electron receptor for all plant copper metalloenzymes (Messerschmidt, 2010), however this has not been proven for BURP domain enzymes.

**TABLE 1:** Plant copper metalloenzyme classes.

Class	EC	Substrate(s)	Catalysis	Copper center (total # of Cu atoms) per subunit	Metabolic pathways	Cellular localization	PDB ID (Cu center state), source organism	References
Laccase	1.10.3.2	Monolignols, oligolignols flavonoids, urushiols	Oxidative coupling (one electron oxidation)	1 T1 + 1 TNC (4 copper atoms)	Lignin, neolignans	Apoplast	6KLG (oxy), 6KLJ (oxy, coniferyl complex), 6KLI (oxy, sinapyl complex), *Zea mays*	Sterjiades et al. (1992), Bao et al. (1993), Xie et al. (2020), Yonekura-Sakakibara et al. (2020)
Ascorbate oxidase	1.10.3.3	Ascorbate	Enediol oxidation (two electron oxidation)	1 T1 + 1 TNC (4 copper atoms)	Cellular redox control	Apoplast, intercellular spaces, vacuole	1AOZ (met), 1ASQ (deoxy, azide complex), 1ASP (peroxy), 1ASO (deoxy), *Cucurbita pepo*	Messerschmidt et al. (1992, 1993b)
Tyrosinase	1.14.18.1	Monophenolic substrates (e.g., tyramine, tyrosine)	Phenol monooxygenation and *o*-diphenol oxidation (two electron oxidation)	1 T3 (2 copper atoms)	*o*-Quinones	Thylakoid lumen	6ELS (met), apple; 5CE9 (met), *Juglans regia*; 6HQI (met), 6HQJ (apo), *Solanum lycopersicum*	Nakayama et al. (2000), Bijelic et al. (2015), (Kampatsikas et al., 2019a,b)
Catechol oxidase	1.10.3.1	*o*-Diphenolic substrates	*o*-Diphenol oxidation (two electron oxidation)	1 T3 (2 copper atoms)	*o*-Quinones	Thylakoid lumen, Golgi apparatus	1BT1 (met), 1BT2 (met) 1BT3 (met), 1BUG (deoxy, inhibitor complex), *Ipomoea batatas*; 2P3X (met), *Vitis vinifera*	Klabunde et al. (1998), Virador et al. (2010)
Aurone synthase	1.21.3.6	Monophenolic substrates (flavonoids, e.g., isoliquiritigenin)	Phenol monooxygenation and *o*-diphenol oxidation (two electron oxidation), flavonoid cyclization	1 T3 (2 copper atoms)	Aurone flavonoids	Vacuole	4Z11 (met) 4Z12 (met), 4Z13 (oxy), 4Z0Y (deoxy), 4Z0Z (deoxy), *Coreopsis grandiflora*	Molitor et al. (2016)
Copper-containing amine oxidase (CuAO)	1.4.3.21 (monoamine oxidases), 1.4.3.22 (diamine oxidases)	Organic amines	Oxidative deamination (aldehyde formation)	1 T2 (1 copper atom)	Tropane alkaloids	Peroxisome, apoplast	1KSI (oxy), *Pisum sativum*	Hashimoto et al. (1990), Kumar et al. (1996), Heim et al. (2007), Naconsie et al. (2014)
Cu,Zn-superoxide dismutase	1.15.1.1	Superoxide, reactive oxygen species	Superoxide dismutation	1 T2 (1 copper atom)	Lignin	Mitochondria, peroxisome, glyoxysome, cytosol, chloroplast, vacuole and tonoplast, nucleus, and extracellular space	1SRD (deoxy), *Spinacia oleracea*	Kitagawa and Katsube (1994)
BURP domain peptide cyclase	Undefined	Core peptide motifs	Oxidative coupling involving tyrosine or tryptophan side chains	N/A	Cyclic peptides	Vacuole, apoplast	N/A	Chigumba et al. (2021)
Plastocyanin	N/A	N/A	Electron transfer	1 T1 (1 copper atom)	Photosynthesis	Thylakoid lumen	1PLC (oxy) *Populus nigra*	Guss et al. (1992)

Copper binding sites found in plant copper metalloenzymes are distinguished based on common biological copper centers, and are denoted as type I (T1 or blue copper), type II (T2 or normal copper), type III (T3 or binuclear copper) and trinuclear copper centers (TNC), which consist of a T2 and a T3 center (Solomon et al., 1996). Laccases and ascorbate oxidases have four copper atoms in a T1 Cu center and a TNC, catechol oxidases and tyrosinases have two copper atoms in a T3 Cu center, and amine oxidases and Cu,Zn-superoxide dismutases have one copper atom in a T2 Cu center (Table 1).

### Laccases

Laccases (EC 1.10.3.2) are multicopper oxidases belonging to the PPO family and are present in bacteria, fungi, and plants. The first laccase was discovered in the Japanese lacquer tree (*Toxicodendron vernicifluum*, formerly *Rhus verniciflua*) as a substance involved in lacquer hardening during wound healing (Yoshida, 1883). The substance was identified by Gabriel Bertrand as an enzyme, which was named laccase (Bertrand, 1894) and subsequently characterized as a copper-dependent oxidase (Keilin and Mann, 1939). Originally it seemed that lacquer tree laccase did not catalyze the polymerization of monolignols and, therefore, laccases were assumed to be involved in lacquer hardening and not lignin formation (Nakamura, 1967). However, *in vivo* and *in vitro* studies on laccases purified from Sycamore maple (*Acer pseudoplatanus*) and loblolly pine (*Pinus taeda*) characterized laccase catalysis of monolignol polymerization and provided the first direct evidence of plant laccase involvement in lignin formation in plant cell walls (Sterjiades et al., 1992; Bao et al., 1993).

Laccases have broad substrate specificity of phenolic substrates with common substrates of lignin and neolignan biosynthesis being the monolignols *p*-coumaryl alcohol (CouA), coniferyl alcohol (ConA) and sinapyl alcohol (SinA). The preferred substrates of the lacquer tree laccase are catechol-containing molecules such as urushiol and lignocatechols yielding a catechol-crosslinked polymer, in lacquer tree sap (Figure 1A; Kumanotani, 1978; Yoshida et al., 2009). In addition, simple phenols, flavonoids and ascorbate acid have been shown to be oxidized by laccases in *in vitro* reconstitution experiments (Sterjiades et al., 1992; Bao et al., 1993), and laccase catalysis can also include crosslinking of tyrosine-side-chains in proteins (Mattinen et al., 2005). This broad substrate specificity of laccases can translate into relatively high *K*_*m*_ values for these tested compounds. For example, ZmLac3, a laccase from maize (*Zea mays*) implicated in lignification (Caparrós-Ruiz et al., 2006), showed *K*_*m*_ values for monolignol substrates SinA and ConA of 346.47 μM and 134.4 μM, respectively (Xie et al., 2020). Laccases such as ZmLac3 also appear to be slow and less efficient enzymes as *k*_*cat*_ values for turnover of monolignols SinA and ConA are 27.8 s^–1^ and 2.98 s^–1^, respectively, and *k*_*cat*_/*K*_*m*_ values are 0.08 μM^–1^s^–1^ (SinA) and 0.022 μM^–1^s^–1^ (ConA). Steady-state kinetic experiments determined a ping-pong mechanism of lacquer tree laccase with a maximum *k*_*cat*_ of 560 s^–1^ (Petersen and Degn, 1978).

**FIGURE 2:**
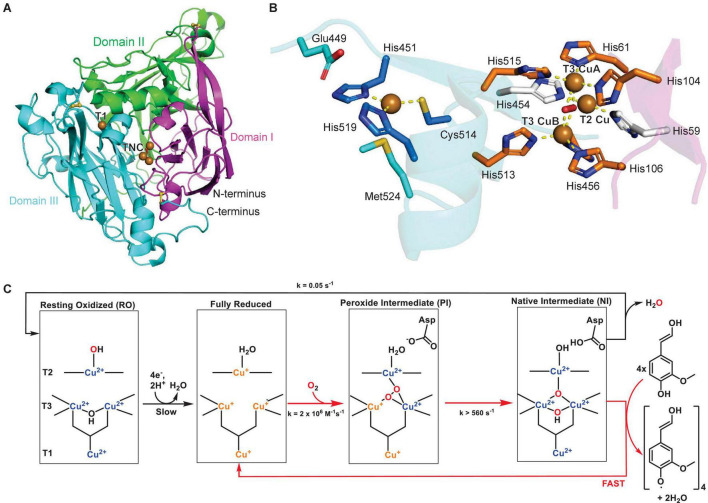
Structure of a plant laccase. **(A)** Protein structure of maize laccase ZmLac3 (PDB ID: 6KLG) (Xie et al., 2020). The three domains are highlighted in magenta, green and blue, the copper atoms are shown as brown spheres and disulfide bonds as yellow sticks. **(B)** Copper center of ZmLac3 laccase with T1 and TNC copper atoms and corresponding copper-binding residues. A water or hydroxide molecule is shown as a red sphere. Copper coordination is highlighted by yellow dashed lines. **(C)** Catalytic mechanism of laccase with catalytic cycle highlighted by red arrows and reduction of resting state (RO) or decay of native intermediate (NI) state to resting state highlighted by black arrows. Adapted with permission from Augustine et al. (2010; Copyright 2010 American Chemical Society).

The characterization of the first protein structure of a plant laccase by Xie et al. (2020) gave insights into substrate binding and catalytic residues of these copper enzymes. The maize laccase structure ZmLac3 revealed three cupredoxin domains (I, II, and III), which included three disulfide bonds (Figure 2A). A high degree of glycosylation ranging from 20 to 45% was previously described for plant laccases (Bligny and Douce, 1983; Bao et al., 1993) and was confirmed by seven characterized surface *N*-glycosylations in ZmLac3. The copper in the T1 Cu center is trigonally coordinated by two imidazole-nitrogens of histidines, His451 and His519, and a thiol-sulfur of cysteine, Cys514. In the TNC center the two T3 copper atoms are coordinated by six histidine-imidazole-nitrogens, and the T2 copper is coordinated by two histidine-imidazole-nitrogens (Figure 2B). Importantly, these characterized copper binding residues are highly conserved in all plant laccases. The substrate binding pocket of ZmLac3 features a conserved hydrophobic wall and a glutamate in the pocket bottom, which is close to the T1 Cu center. This residue, Glu449, is hypothesized to favor substrate binding via hydrogen bond formation to the phenoxy- and methoxy-groups of monolignol substrates and act as a deprotonation base during the catalytic cycle. Crystal structures of ZmLac3 bound to its two major lignin biosynthetic building blocks, SinA and ConA, revealed distinct orientations of these substrates in the binding pocket. The different positioning of these monomers is due to interactions with their differing numbers of methoxy groups with the substrate binding pocket, which results in closer positioning of the phenoxy group of SinA to both the T1 copper center, the site of substrate oxidation, and to glutamate 449, the presumed proton acceptor in the laccase catalytic mechanism. This closer binding of SinA to the T1 Cu center is hypothesized to cause an almost four times more efficient turnover of SinA compared to ConA by ZmLac3. Subtle differences in the substrate binding pocket as identified by Xie *et al.* in ZmLac3 could therefore contribute to different ratios of monolignol monomers in lignin (Xie et al., 2020).

The catalytic mechanism of laccases was characterized based on enzymatic studies of the original plant laccase from Japanese lacquer tree and of the closely related ascorbate oxidase (Augustine et al., 2010). In general, the TNC center catalyzes the reduction of dioxygen to water, whereas the T1 Cu center oxidizes four phenolic substrates per catalytic cycle (Figure 2C). Before the first step, the enzyme is in the resting state (RO), in which all copper atoms are oxidized and the T3 Cu atoms are bridged by a hydroxide. The T1 Cu then reduces four phenolic substrates consecutively and transfers three of the electrons to the TNC center to yield a fully reduced active site. The reduced TNC center then binds dioxygen in a fast step to yield the peroxide intermediate (PI). Herein, one T3 copper and the T2 copper are oxidized, while one oxygen atom is coordinated between the T3 copper atoms and the other oxygen atom is coordinated by the T2/T3 Cu(II) atoms in a μ3-1,1,2 bridging mode (Solomon et al., 2008). Subsequently, the dioxygen bond is cleaved in the rate-limiting step of the catalytic cycle, which results in all T3 coppers and the T1 copper being oxidized to Cu(II) in the native intermediate (NI). In the final step, the oxidized T1 copper abstracts four protons and four electrons from phenolic substrates such as monolignols and the TNC-bound oxygen species are reduced and released as water. This step is fast and results in the return to the fully reduced active site (Figure 2C; Lee et al., 2002; Solomon et al., 2014).

After formation of monolignol phenoxy radicals by plant laccases, the radical center can delocalize throughout the phenylpropanoid structure and subsequently quench by coupling with another monolignol radical. The dimerization of monolignol radicals can be controlled in a region- and stereoselective manner by non-catalytic dirigent proteins (Davin et al., 1997). Interestingly, plants have evolved dirigent proteins which enable enantiocomplementary formations of dilignols such as (+)- or (−)-pinoresinol (Pickel et al., 2010) via laccase-catalyzed oxidative coupling.

### Ascorbate Oxidases

Ascorbate oxidase (EC 1.10.3.3) was characterized in plant tissues as an enzyme which oxidizes ascorbate under aerobic conditions (Szent-Györgyi, 1930). Like laccases, ascorbate oxidases are a member of the multicopper enzyme class, which has a T1 Cu center and a TNC center and which catalyzes the reduction of dioxygen to water via four one-electron oxidations of ascorbate to a semidehydroascorbate radical (Marchesini et al., 1977). There are reductases for semidehydroascorbate radicals (Bérczi and Møller, 1998) that will regenerate ascorbate, despite the short lifetime of the radical (Noctor and Foyer, 1998). Ascorbate oxidases have also shown *in vitro* activity toward phenolic substrates, likely because of their similarity to laccases (Marchesini et al., 1977).

**FIGURE 3:**
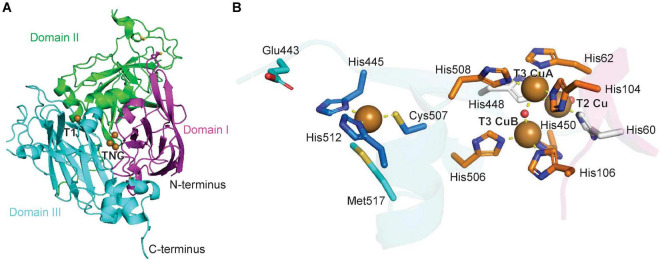
Structure of a plant ascorbate oxidase. **(A)** Protein structure model of zucchini ascorbate oxidase (PDB ID: 1AOZ) (Messerschmidt et al., 1992). The three domains are highlighted in magenta, green and blue, the copper atoms are shown as brown spheres. **(B)** Copper center of zucchini ascorbate oxidase with T1 and TNC copper atoms and corresponding copper-binding residues. A water or hydroxide molecule is shown as a red sphere. Copper coordination is highlighted by yellow dashed lines.

The structure of zucchini ascorbate oxidase has been solved and is a homodimer of a subunit similar to the laccase structure. The ascorbate oxidase monomer also consists of three cupredoxin domains, a T1 Cu center and a TNC center (Figure 3). The copper binding sites are conserved between laccases and ascorbate oxidases, including the axial coordination of the T1 copper atom by a methionine-sulfur (Figures 2B, 3B; Messerschmidt, 2010). For zucchini ascorbate oxidase, X-ray structures of the fully oxidized state, fully reduced state and the peroxide intermediate state were generated, which revealed significant changes in the TNC copper site between each state, whereas the T1 Cu center remained structurally unchanged (Messerschmidt et al., 1992, 1993a,b). The catalytic mechanism and kinetic model for laccases also applies to ascorbate oxidases with ascorbate as a reducing agent based on mechanistic studies of intermediate species in both enzyme reactions (Figure 2C; Solomon et al., 2014).

### Type III Polyphenol Oxidases

Plant catechol oxidases (CO, EC 1.10.3.1), tyrosinases (TYR, EC 1.14.18.1) and aurone synthases (AUS, 1.21.3.6) are T3 multicopper enzymes. Catechol oxidases were first discovered in plants as enzymes associated with defense-related fruit browning (Szent-Gyrgyi and Vietorisz, 1931) and tyrosinases were characterized later in plants as catechol oxidases associated with monophenolase activity (Robb et al., 1966; Harel and Mayer, 1971; Kahn and Pomerantz, 1980; Zekiri et al., 2014). Like laccases, catechol oxidases, tyrosinases and aurone synthases belong to the PPO family but are distinct from laccases in that they only have a T3 copper center, and they catalyze two two-electron-oxidation reactions to reduce one dioxygen to water (Table 1). Catechol oxidases, tyrosinases and aurone synthases all catalyze the oxidation of *o*-diphenols (diphenolase reaction), whereas tyrosinases and aurone synthases also catalyze the monooxygenation of monophenols to *o*-diphenols (monophenolase reaction). Despite this clear theoretical distinction in catalysis between COs and TYRs, many COs have been reported with varying degrees of monophenolase activity, which can complicate the differentiation of type III polyphenol oxidases (T3 PPOs) into COs or TYRs due to putative evolutionary transition states between both enzyme activities (Robb et al., 1966; Harel and Mayer, 1971; Kahn and Pomerantz, 1980; Zekiri et al., 2014). A special case of T3 PPO biochemistry is (+)-larreatricin hydroxylase from *Larrea tridentata*, which only shows monophenolase activity (Cho et al., 2003). Catechol oxidases purified from plants have broad substrate specificity for substituted catechols, including flavonoids such as catechin, amines such as dopamine, and amino acids such as L-DOPA (Flurkey and Jen, 1980; Paul and Gowda, 2000). Tyrosinases also show broad substrate specificity in mono- and diphenol substrates (Robb et al., 1966; Harel and Mayer, 1971; Kahn and Pomerantz, 1980; Zekiri et al., 2014) related to their primary substrate tyrosine. An exception to this substrate-based definition of tyrosinases are aurone synthases (AUS), which do not accept tyrosine but rather use chalcones as substrates, while possessing both mono- and diphenolase activity (Nakayama et al., 2000; Molitor et al., 2016). Given the overlap of plant CO and TYR enzyme activities and few characterized endogenous substrates of plant T3 PPOs, with the exception of aurone synthases, an update of T3 PPO nomenclature independent of catechol- and tyrosine-substrate specificity has been proposed and might be needed in the future with more knowledge about endogenous PPO substrate space (Molitor et al., 2016).

**FIGURE 4:**
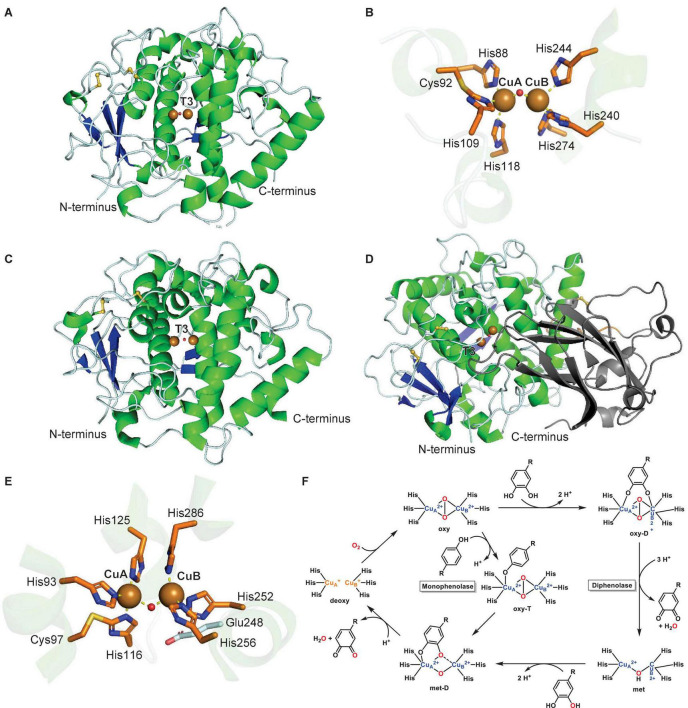
Structures and mechanism of plant type III polyphenol oxidases. **(A)** Protein structure of active form of sweet potato catechol oxidase *Ip*CO (PDB: 1BT1) (Klabunde et al., 1998). **(B)** T3 copper center of sweet potato catechol oxidase *Ip*CO. **(C)** Protein structure of active walnut tyrosinase *Jr*TYR (PDB ID: 5CE9) (Bijelic et al., 2015). **(D)** Protein structure of latent aurone synthase *Cg*AUS (PDB: 4Z11) (Molitor et al., 2016). C-terminal shielding domain is highlighted in dark gray. **(E)** T3 copper center of walnut tyrosinase *Jr*TYR. **(F)** Catalytic mechanism of monophenolase reaction (inner cycle) and diphenolase reaction (outer cycle) in plant type III polyphenol oxidases. Adapted with permission from Solomon et al. (2014; Copyright 2014 American Chemical Society). In PPO protein structures, α-helices are highlighted in green, β-sheets in blue, disulfide bonds in yellow, copper atoms are brown spheres and water or hydroxides are red spheres. Copper coordination is highlighted in copper center figures by yellow dashed lines.

The first crystal structure of a plant T3 PPO, *Ip*CO, a catechol oxidase, from sweet potato (*Ipomoea batatas*) revealed a monomeric protein with a central four-helix-bundle, which contains the T3 copper center (Klabunde et al., 1998; Figure 4A). Each of the two copper atoms, CuA and CuB, is coordinated by three histidines, with CuA and CuB 2.9 Å apart in the oxidized copper center. One of the CuA-coordinating histidines, His109, is covalently linked in its Cε to the sulfur atom of a cysteine, Cys92 (Figure 4B). This thioether bridge has also been characterized in other T3 Cu centers of copper-containing proteins such as the oxygen transporter hemocyanin (Lerch, 1982; Gielens et al., 1997). The *Ip*CO structure contains a hydrophobic substrate binding pocket for its phenolic substrates as highlighted by a protein structure with a bound aromatic PPO inhibitor (Klabunde et al., 1998) as a substrate mimic. The first crystal structure of a plant tyrosinase, the walnut enzyme *Jr*TYR (*Jr*PPO1), showed high similarity to its catechol oxidase counterpart in overall protein fold and in the T3 copper center (Figures 4C,D; Bijelic et al., 2015). *Jr*TYR was crystallized in the resting met form, in which a hydroxide or water molecule is bound in between the CuA and CuB atoms, which are 4.2 Å apart. Both crystal structures of *Ip*CO and *Jr*TYR represent the catalytic domains of these PPOs.

In general, plant PPOs are ∼600-amino-acid-long proteins with an N-terminal domain of signaling and transit peptides, a central catalytic domain and a C-terminal shielding domain. The N-terminal domains are cleaved during PPO transport to their cellular destination, the thylakoid lumen, where they are activated by proteolytic separation of the C-terminal shielding domain (Marusek et al., 2006; Virador et al., 2010). A recent study of an apple PPO (*Md*PPO1) showed that this activation can occur by self-cleavage in the linker region between the catalytic and shielding domain. The self-cleavage event occurs in a sequence-independent manner in a four-amino-acid linker region, which is located four residues C-terminally of a cleavage-inducing nine-amino-acid peptide sequence (Kampatsikas et al., 2019a). The lack of this self-cleavage-inducing peptide in other plant PPOs prevents autocatalytic activation (Kampatsikas et al., 2019a). In addition to CO and TYR catalytic domains, the structure of an aurone synthase from *Coreopsis grandiflora*, *Cg*AUS, has been characterized in its catalytic domain and its latent state, i.e., including the C-terminal shielding domain. This latent PPO structure revealed that the shielding domain blocks access to the T3 Cu active site by an isoleucine “plug” residue above the active site entrance (Figure 4E; Molitor et al., 2016).

The structures of catechol oxidase *Ip*CO, tyrosinase *Jr*TYR and aurone synthase *Cs*AUS led to structure-based hypotheses of determinants for monophenolase and diphenolase activity. A phenylalanine residue near CuA in *Ip*CO was initially proposed to be a “gatekeeper” residue preventing monophenolase activity in catechol oxidases due to its interaction with PPO substrates (Klabunde et al., 1998). However, this hypothesis was rejected based on the presence of the same phenylalanine in walnut tyrosinase *Jr*TYR (*Jr*PPO1) (Bijelic et al., 2015). In addition, mechanistic studies on bacterial and plant tyrosinases indicated that a conserved water activated by asparagine (HB1+1) where HB-1 is a conserved copper-coordinated histidine residue and glutamate (HB1-4) “waterkeeper” residues is involved in deprotonating monophenolic substrates before CuA binding in tyrosinases (Goldfeder et al., 2014; Solem et al., 2016). This hypothesis was partially disproven by site-directed mutagenesis studies on dandelion PPOs, which showed TYR activity independent of the asparagine residue (Prexler et al., 2019) and a proposed distinction between CO and TYR activity in T3 PPOs based on residues surrounding the CuB binding site such as HB1+1 and HB2+1 (Pretzler and Rompel, 2018; Prexler et al., 2018). Furthermore, a site-directed mutagenesis study of residues adjacent HA1, HB1 and HB2 of the T3 Cu center of *Cg*AUS showed that the cysteine residue forming a thioether to HA1 and the two residues adjacent to HB1 and HB2 (HB1+1 and HB2+1) can alter the activity profile of aurone synthase to a tyramine monophenolase of catalytic capacity comparable to dedicated plant tyrosinases (Kampatsikas et al., 2020). Based on these recent mutagenesis studies on plant PPOs (Panis and Rompel, 2020), a catalytic model is emerging in which residues adjacent to three conserved copper-binding histidines (HA1, HB1, and HB2) dictate the histidines’ ability to aid in deprotonation of monophenol substrates and, thereby, enable monophenolase activity of a plant PPO (Kampatsikas et al., 2020). In addition, dandelion PPOs could be distinguished as either COs or TYRs based on phylogenetic separation into two distinct groups (Prexler et al., 2019). Further enzymatic activities of plant PPOs on endogenous substrates beyond canonical mono- and diphenol substrates will enable a better prediction of PPO functions in plant metabolism from a given PPO sequence.

To date, structures of four forms – deoxy, oxy, met (resting state), and inhibitor-bound – of plant T3 PPO Cu centers have been solved, which enabled together with spectroscopic and kinetic studies the formulation of a catalytic mechanism of the diphenolase reaction (Table 1 and Figure 4F – outer cycle) (Solomon et al., 2014). In this catalytic cycle, the enzyme requires two *o*-diphenol substrates and one dioxygen. The first *o*-diphenol binds to the resting met-state of the copper center, in which a hydroxide is bound between two Cu(II) atoms, to form a met-D state. The diphenol is subsequently oxidized to the *o*-quinone and released together with a water, resulting in a reduced (deoxy) copper center. Next, dioxygen is bound between the copper atoms in its oxy state, which binds the second *o*-diphenol substrate between the Cu(II) atoms in an oxy-T state. Oxidation of the diphenol to the *o*-quinone and subsequent release together with another water molecule completes the diphenolase cycle in the met state. The monophenolase reaction (Figure 4F – inner cycle) is based on studies of several plant tyrosinases and aurone synthase *Cg*AUS (Table 1). In this cycle, one monophenol substrate is oxidized to the *o*-quinone by turnover of one dioxygen. First, the deoxy copper center binds dioxygen to form its oxy state. Next, three conserved copper-binding histidines (HA1, HB1, and HB2) together with the “waterkeeper” glutamate (Figure 4E, Glu248) deprotonate the monophenol hydroxyl group and catalyze its binding to CuA in the oxy-T state. The monophenol is then oxygenated at the ortho-position. Subsequently, the corresponding *o*-quinone is released with one water to complete the monophenolase cycle in the deoxy state. Crystallographic studies on aurone synthase revealed that dioxygen binds initially between the copper atoms in PPO catalytic cycles in butterfly distorted orientations, which switches to an inverse butterfly distorted orientation upon phenol substrate binding (Molitor et al., 2016).

### Copper-Containing Amine Oxidases (CuAO)

**FIGURE 5:**
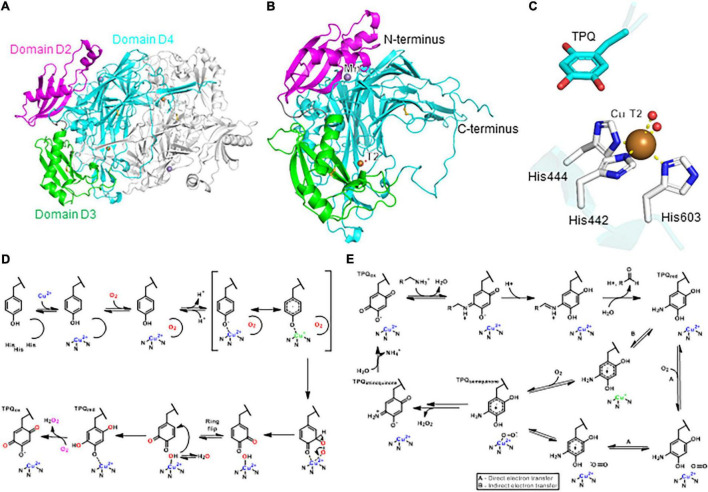
Structure and mechanism of a plant copper-dependent amine oxidase. **(A)** Homodimer protein structure of pea copper-dependent amine oxidase (PDB ID: 1KSI) (Kumar et al., 1996). **(B)** Monomer structure of pea CuAO. **(C)** T2 copper center with copper-binding residues and TPQ cofactor. Copper coordination is highlighted by yellow dashed lines. **(D)** Proposed mechanism of TPQ biogenesis. Adapted with permission from Dubois and Klinman (2005; Copyright 2005 Elsevier). **(E)** Proposed catalytic mechanism of CuAO. Adapted with permission from Mills et al. (2019; Copyright 2019 Springer Nature). In panels **(A,B)**, the three domains D2, D3, and D4 are highlighted in magenta, green, and cyan, respectively. In panels **(A–C)**, copper atoms are shown as brown spheres, manganese atoms as purple spheres, water molecules as red spheres.

Plant copper-containing amine oxidases are classified as E.C. 1.4.3.21 for primary amine oxidases (Schomburg and Schomburg, 2013b), and E.C. 1.4.3.22 (Schomburg and Schomburg, 2013a) for oxidation of diamines. Amine oxidases catalyze the deamination of primary amines and diamines to aldehydes by consumption of molecular oxygen and water with release of ammonia and hydrogen peroxide (Solomon et al., 2014). Copper-containing amine oxidases (CuAOs) have faster turnover and higher affinity for putrescine and cadaverine as substrates, and with less specificity spermidine and spermine (Padiglia et al., 1998; Agostinelli et al., 2005; Poonpipatgul, 2012; Tavladoraki et al., 2016; Zhang et al., 2016). Apoplastic CuAO can also oxidize long chain aliphatic and aromatic monoamines, e.g., 2-phenylethylamine and tyramine, *in vitro* (Zarei et al., 2015). The first analysis of plants for amine oxidases was in the 1940s (Cromwell, 1943; Werle and Raub, 1948; Mann, 1955; Andresen et al., 2018), although definitive evidence for copper as a cofactor was not discovered until 1961 in pea seedlings (*Pisum sativum*) (Mann, 1961; Andresen et al., 2018). These amine oxidases are characterized by a mononuclear T2 copper center and a protein-derived cofactor, 2,4,5-trihydroxyphenylalanine quinone (TPQ). TPQ was discovered in 1990 for bovine serum amine oxidase (Janes et al., 1990), and confirmed in pea and chickpea seedling CuAO in 1992 (Janes et al., 1992). Shortly thereafter, the first protein structure of a plant CuAO from pea seedling was determined (Kumar et al., 1996). It crystallized as a homodimer, and each subunit was about 73 kDa (Tipping and McPherson, 1995; Figure 5A). There are two disulfide bonds per subunit (not involved with catalysis) and four possible locations for N-linked glycosylation on pea CuAO based on the consensus sequence of Asn-X-Thr/Ser. All of these locations are on the surface of the structure, with confirmed electron density for sugars at Asn131 and Asn158 (Kumar et al., 1996). The pea CuAO can be separated into three domains, named after the structurally similar *E. coli* CuAO domains (Kumar et al., 1996). Domains D2, D3, and D4 are conserved (Figure 5B), where D4 contains the buried active site of a T2 Cu(II) and TPQ cofactor (Figure 5C). The Cu(II) is coordinated by three histidine residues and two waters. The two waters do not form hydrogen bonds to any nearby residues, nor to the nearby TPQ covalent cofactor in this resting state. CuAO from different plants can have sequence identity as low as 25%, but the residues surrounding the catalytic sites are almost entirely conserved (Planas-Portell et al., 2013).

Cu(II) aids in formation of TPQ in a CuAO-autocatalytic mechanism shown in Figure 5D (Schwartz et al., 2000; Kim et al., 2002; Dubois and Klinman, 2005; Davidson, 2011, 2020; Klinman and Bonnot, 2014). TPQ biosynthesis is a multi-step process, which begins with Cu(II) bound in the active site. Once dioxygen is bound near the active site of the enzyme, a conformational change instigates the binding of copper to the tyrosine hydroxyl group (Dubois and Klinman, 2005). UV-vis spectroscopy supports a ligand-metal charge transfer, in which Cu(II) becomes Cu(I) concomitantly with radical generation on the tyrosine (Dove et al., 2000). After the first, irreversible oxidation of the flexible active site tyrosine, the electrophilic ring of dopaquinone flips, giving access to the nucleophilic hydroxyl bound to copper, which originates from water rather than dioxygen based on radioisotope labeling studies (Nakamura et al., 1996; Klinman and Bonnot, 2014). Although the biosynthetic pathway of TPQ formation in CuAO was largely determined in bacteria, yeast, and human cells, it is likely also applicable to plant CuAO due to the high overall similarity in the active site architectures, despite sequence similarities of only 20–25% (Kumar et al., 1996). An alternative mechanism for TPQ biogenesis has been proposed for a bacterial CuAO, which does not involve a Cu(I) intermediate, and remains to be determined for plant CuAO TPQ biosynthesis (Adelson et al., 2019; Shoji et al., 2020). TPQ formation can be identified in a protein sequence by the consensus sequence Asn-Tyr-Asp/Glu, in which tyrosine becomes TPQ (Janes et al., 1992; Mu et al., 1992).

Despite the lack of crystallographic CuAO structures from plants, numerous studies provide kinetic and crystallographic data for CuAOs from other organisms, which suggest a mechanism for plant CuAO (Figure 5D; Medda et al., 1998; Agostinelli et al., 2005; Solomon et al., 2014). The proposed double displacement, or ping-pong, catalytic mechanism of CuAO can be distilled into two half reactions: a substrate-dependent reduction of TPQ, and a subsequent oxygen-dependent re-oxidation of TPQ. The primary amine substrate functions through a Schiff-base on TPQ (Figure 5E) before the cofactor is re-oxidized (Angelini et al., 2018). In order to regenerate TPQ, Cu(II) bound to reduced TPQ is in equilibrium with Cu(I) bound to the semiquinone version of TPQ. The superoxide attacks the semiquinone of the TPQ ring, and proton-coupled electron transfer results in the iminoquinone form of TPQ with release of hydrogen peroxide (Dooley et al., 2017). Lastly, the release of ammonia in the presence of water re-oxidizes TPQ (Mills et al., 2012). The oxidative half-reaction uses indirect, inner-sphere electron transfer, and is demonstrated in pea seedling CuAO (Mills et al., 2012, 2019). The outer-sphere electron transfer mechanism, where reduced TPQ transfers an electron to dioxygen directly so the oxidization state of Cu(II) is constant, is used in fungi (Mills et al., 2019). It remains to be seen whether the outer-sphere, direct electron transfer without a change in Cu(II) oxidation state will apply to other plant CuAOs (Pietrangeli et al., 2003).

### Cu,Zn-Superoxide Dismutase (Cu,Zn-SOD)

**FIGURE 6:**
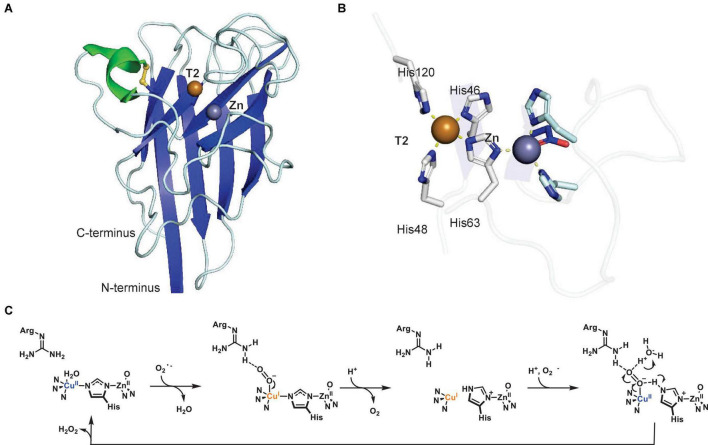
Structure of a plant Cu,Zn-superoxide dismutase. **(A)** Protein structure of spinach Cu,Zn-superoxide dismutase (PDB ID: 1SRD) (Kitagawa and Katsube, 1994). **(B)** T2 copper center of spinach Cu,Zn-SOD. Copper coordination is highlighted by yellow dashed lines. In panels **(A,B)**, α-helices are highlighted in green, β-sheets are highlighted in blue. Copper atoms are shown as brown spheres, zinc atoms as purple spheres. **(C)** Proposed catalytic mechanism of Cu,Zn-SOD. Adapted with permission from Tainer et al. (1983; Copyright 1983 Springer Nature).

Cu,Zn-superoxide dismutase (Cu,Zn-SOD, E.C. 1.15.1.1) was first isolated in 1969 from bovine erythrocytes as an enzyme that scavenges molecular oxygen radicals and converts them to hydrogen peroxide and molecular oxygen, as an enzymatic defense mechanism for the radical by-products of aerobic metabolism (McCord and Fridovich, 1969). The structure of mammalian Cu,Zn-SOD was determined in 1975 (Richardson et al., 1975), but the first structure from plants was not solved until 1991 from spinach (Kitagawa et al., 1991). The overall fold of spinach Cu,Zn-SOD has a monomer size of 16 kDa and forms a flattened β-barrel with one stabilizing disulfide bond (Figure 6A). The protein forms a homodimer in solution, but contains four subunits in the crystal structure. The oligomeric state of Cu,Zn-SOD depends on the isoform: the cytoplasmic form is a homodimer, whereas those in chloroplasts are homotetramers (Bordo et al., 1994; Alscher et al., 2002). Each active site functions independently and both Cu and Zn atoms are found in the active site, each coordinated by four residues (Figure 6B). Only a histidine residue separates the T2 Cu and Zn ions.

The mechanism of superoxide dismutation remains as originally proposed in 1983, and appears supported by the subsequently published plant crystallographic structure (Tainer et al., 1983). The redox reaction of Cu,Zn-SOD is carried out by the active site Cu, where Zn is strictly a structural component (Figure 6C). The Cu(II) becomes Cu(I) upon binding of the first superoxide in an axial position, which is stabilized and protonated by a conserved active site arginine (Messerschmidt, 2010). Subsequently, superoxide is transformed to dioxygen and released, while the histidine bridging Cu and Zn breaks the bond to Cu (Sheng et al., 2014). Another superoxide then enters the active site, binds Cu, and is transformed to and released as hydrogen peroxide, while Cu(I) is oxidized to Cu(II) and re-establishes the bond to the histidine shared with Zn (Tainer et al., 1983).

### BURP Domain Peptide Cyclases

**FIGURE 7:**
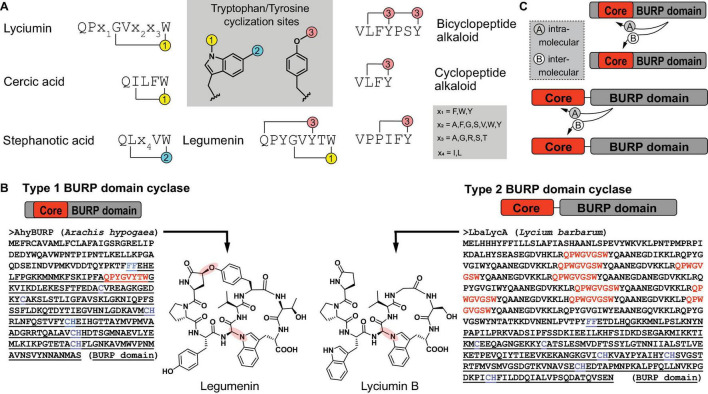
Primary structure of BURP domain peptide cyclases. **(A)** Characterized core peptide substrate sequences of known BURP domain peptide cyclases and characterized tyrosine- or tryptophan-derived macrocyclization sites (1–3). **(B)** Primary structures of representatives of the two general types of BURP domain cyclases and corresponding peptide natural products. In the BURP domain sequence, core peptides corresponding to the natural product are highlighted in red, the BURP domain sequence is underlined, the BURP domain-defining residues are highlighted in blue. **(C)** Two models of autocatalysis for BURP domain-based peptide macrocyclization.

BURP domain peptide cyclases constitute a new class of copper-dependent peptide cyclases, which catalyze the formation of macrocyclic bonds between amino acid side chains (Chigumba et al., 2021). These crosslinks generally involve the side chain of a tyrosine or tryptophan and an unactivated carbon of another amino acid side chain (Kersten and Weng, 2018; Chigumba et al., 2021). BURP domain macrocyclization sites are the indole nitrogen and the C6 position on tryptophan substrates and the phenol hydroxyl group on tyrosine substrates (Figure 7A). Characterized bonds include C(sp^3^)-C(sp^2^) in stephanotic acid by CcaBURP2, a BURP domain protein from Eastern redbud (*Cercis canadensis*), C(sp^3^)-N, where N is the indole nitrogen of a tryptophan, in legumenin and lyciumin I by AhyBURP, a BURP domain from peanut (*Arachis hypogaea*), and C(sp^3^)-O, where O is the phenol-hydroxyl-group of a tyrosine, in cyclopeptide alkaloids such as selanine A and B by SkrBURP, a BURP domain protein from African clubmoss (*Selaginella kraussiana*) (Figure 1H; Chigumba et al., 2021). To date, BURP domains have been characterized to be involved in the biosynthesis of six classes of ribosomally-encoded and posttranslationally-modified peptides (RiPPs) (Arnison et al., 2013) in plants: lyciumins, legumenin, cercic acid, stephanotic acid, monocyclic and bicyclic cyclopeptide alkaloids (Kersten and Weng, 2018; Chigumba et al., 2021). The substrates of BURP domains are short sequence motifs called core peptides, which are produced by ribosomal biosynthesis and, for characterized BURP domain peptide cyclases, are encoded in the same polypeptide as the BURP domain cyclase. Identified BURP domain core peptides have a C-terminal tyrosine or tryptophan and either an N-terminal glutamine, which is transformed to a pyroglutamate, or an N-terminal valine, which is *N*,*N*-dimethylated (Figure 1H). Cyclization residues coupling to the tryptophan or tyrosine are pyroglutamate, isoleucine, leucine, tyrosine, proline and glycine, with each being activated at a C(sp^3^)-H bond for macrocyclization.

BURP domain peptide cyclases are plant proteins named after the first letters of their four founding members, BNM2, a microspore-derived embryo protein from *Brassica napus* (Treacy et al., 1997), USP, an unidentified seed protein from *Vicia faba* (Bassüner et al., 1988), RD22, a drought-responsive protein from *Arabidopsis thaliana* (Yamaguchi-Shinozaki and Shinozaki, 1993) and PG1β, the β-subunit of polygalacturonase isozyme 1 involved in fruit ripening from *Solanum lycopersicum* (Zheng et al., 1992). These BURP domain proteins were defined by a conserved CHX_10_CHX_25–27_CHX_25_-_26_CH sequence motif identified in a C-terminal protein domain with an N-terminal FF-motif (Hattori et al., 1998). The BURP domain was recently characterized in a precursor peptide for lyciumins from Chinese wolfberry (*Lycium barbarum*) by transient expression of the corresponding BURP domain precursor peptide LbaLycA in *Nicotiana benthamiana* and subsequent detection of lyciumin chemotypes in transgenic tobacco leaf tissue, which established this domain being connected to RiPP biosynthesis in plants (Kersten and Weng, 2018). Subsequently, several BURP domains associated with plant RiPPs could be reconstituted *in vitro* in the presence of Cu(II), proving the catalytic role of BURP domains (Chigumba et al., 2021). Based on these studies, two general types of BURP domain precursor peptides can be distinguished. Type I BURP domain precursor peptides encode their core peptides within the BURP domain and the only biochemically characterized representative for a type I BURP domain cyclase is the legumenin precursor AhyBURP from peanut, which only has one core peptide in the N-terminal end of its BURP domain sequence. Type II BURP domain precursor peptides encode their core peptides in an N-terminal domain, which is separate from the BURP domain and often repetitive. Characterized type II BURP domain precursors are selanine cyclase SkrBURP, stephanotic acid-[LV] cyclase CcaBURP2, and cercic acid cyclase CcaBURP1. The repetitive substrate domains of type II BURP domain cyclases can encode multiple copies of either the same or different core peptides, yielding either one (cercic acid by CcaBURP1) or multiple cyclic peptides (lyciumin A, B, and D in LbaLycA) (Figure 7B; Kersten and Weng, 2018; Chigumba et al., 2021). To date, no protein structure of a BURP domain cyclase has been reported so that the Cu center in this new copper enzyme class remains to be determined.

An interesting feature of BURP domain catalysis is its autocatalytic mechanism as the enzyme also constitutes the substrate. Autocatalysis is a common regulatory mechanism in protein kinases (Dodson et al., 2013) but it is rare in natural product biosynthesis, with the only other example being peptide-*N*-methyltransferases involved in fungal RiPP biosynthesis (van der Velden et al., 2017). Structural and mechanistic studies will reveal if type I and II BURP domain cyclases catalyze macrocyclizations of their core peptides inter- or intramolecularly (Figure 7C; Dodson et al., 2013). In addition, it remains to be determined if BURP domains require dioxygen for catalysis such as all copper metalloenzymes discussed in this review and if BURP domains require other cofactors for multi-turnover catalysis as so far only single-turnover catalysis has been shown *in vitro* for BURP domain cyclases. Finally, spectroscopic studies will reveal if a radical oxidative mechanism underlies the formation of tryptophan- and tyrosine-macrocycles in BURP domain precursors (Chigumba et al., 2021). Interestingly, several macrocyclic bonds formed by BURP domain cyclases are similar to bonds formed by bacterial radical SAM Fe-S-cluster peptide cyclases, which are oxygen-sensitive enzymes (Broderick et al., 2014; Schramma et al., 2015; Imai et al., 2019; Nguyen et al., 2020). BURP domain cyclases and radical SAM Fe-S-cluster cyclases could therefore be an interesting example of convergent evolution of cyclic RiPP chemistry via aerobic copper-dependent cyclases in plants and anaerobic iron-based cyclases in bacteria.

### Plastocyanin

**FIGURE 8:**
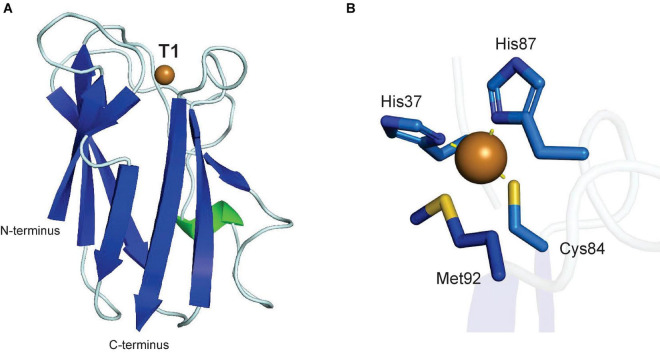
Structure of a plant plastocyanin. **(A)** Protein structure of poplar plastocyanin (PDB: 1PLC) (Guss et al., 1992). **(B)** T1 copper center of poplar plastocyanin. In panels **(A,B)**, α-helices are highlighted in green, β-sheets are highlighted in blue. Copper atoms are shown as brown spheres.

Although it is not a metabolic enzyme, plastocyanin is a copper-containing plant protein, which is essential to plant energy metabolism as it catalyzes electron transport between the cytochrome b_6_f complex of photosystem II and photosystem I in the thylakoid lumen during photosynthesis (Redinbo et al., 1994; Höhner et al., 2020). Plastocyanin is a 10.5 kDa protein with a single type I Cu center, which defines it as a blue copper protein based on its high absorbance at 600 nm. The first plastocyanin structure was reported for a poplar protein in 1978 and revealed a type I copper center, in which the single copper atom is coordinated by the side chains of a cysteine, two histidines and a methionine (Figure 8; Colman et al., 1978; Guss et al., 1992). The coordination of the plastocyanin copper atom by two histidine-imidazole-nitrogen atoms, which prefer Cu(II) binding, and two sulfur atoms from cysteine and methionine side chains, which prefer Cu(I), allow plastocyanin to interchange its copper site between Cu(II) and Cu(I) in a quasi-tetrahedral geometry during electron transfer from PSII to PSI (Colman et al., 1978; Redinbo et al., 1994). A current model of electron transfer mechanism from cytochrome b_6_f involves copper-binding residue His87 after binding an acidic patch on the plastocyanin surface (Ubbink et al., 1998). Plastocyanin contains a signal and transit peptide which directs it to the thylakoid lumen (Smeekens et al., 1985).

## Localization and Biological Function of Plant Copper Metalloenzymes

### Laccases

Laccases are localized in the plant cell wall, where they are involved in the biosynthesis of lignin, an essential plant polymer for mechanical support and defense of terrestrial plants. Lignin is the most stable portion of the plant cell wall lignocellulose due to its heterogeneous linkages including C-C-bonds between its monomers. In most recent proposals for lignin biosynthesis, laccases are anchored to the secondary cell wall (Yi Chou et al., 2018), where they are responsible for initiating lignin polymerization by oxidative coupling of starting lignin monomers such as the canonical monolignols SinA, CouA and ConA (Dixon and Barros, 2019). In addition to the canonical monolignols, laccases can also catalyze the free radical oxidation of γ-acylated monolignols and caffeyl alcohol (Tobimatsu and Schuetz, 2019; Wang et al., 2020). The homopolymer of caffeyl alcohol is catechyl lignin, which can be found in the seed coats of a few non-crop plants (Wang et al., 2020). Wang et al. determined that a specific laccase that forms catechyl lignin in *Cleome hassleriana* has substrate specificity for caffeyl and sinapyl alcohol, but no reaction with coniferyl alcohol. In addition to monolignols, the possibility of flavonoids being involved in lignin initiation reactions is entertained (Dixon and Barros, 2019) as these can function as laccase substrates due to laccases’ broad phenolic substrate specificity and because tricin has been characterized as a monomer in lignin of some grass species (Lan et al., 2015). The di- or oligolignol products of cell wall laccases are then used as substrates by cell wall class III peroxidases to elongate lignin polymers with monolignol substrates (Dixon and Barros, 2019). Both class III peroxidases and laccases can contribute to lignin formation by catalyzing monolignols to free radicals. Class III peroxidases use iron and hydrogen peroxide, whereas the laccases use oxygen and copper, which exemplifies the aforementioned ability of copper enzymes to mimic iron-based catalysis in plant metabolism.

Due to their role in lignin biosynthesis, laccases have important functions in vascular plant growth and plant defense. Genetic studies showed that plant laccases are important for vascular tissue growth by their role in lignin biosynthesis. For example, a triple laccase mutant knockout of *Arabidopsis thaliana* showed severe vascular growth defects compared to the wild-type plant (Zhao et al., 2013), as the mutant had almost no lignin within the stem, but it showed normal root development. This study showed that the Casparian strip, a ring-like lignin structure in the endodermis in the roots of vascular plants is formed by class III peroxidases independent of laccases. Besides their effect on plant development through lignin formation, laccases influence plant development by crosslinking flavonoids, too. For instance, a laccase knockout mutant in *Arabidopsis thaliana* resulted in an altered seed color linked to an accumulation of soluble proanthocyanidin. The altered seed coat browning is linked to the laccases ability to catalyze oxidative polymerization of flavonoids (Pourcel et al., 2005). Laccases contribute to plant defense by increasing lignin production when a more protective material is needed (Hu et al., 2018; Zhang et al., 2019). The down-regulation of laccase can lead to reduced lignin production and, therefore, more susceptibility to pests such as cotton aphids (Hu et al., 2018). In addition to lignin formation, laccases are also part of the biosynthesis of other plant-protective polymers like lacquer. Herein, laccases have been found in the resin ducts and resin in all family members of Anarcardiaceae, which include the Japanese lacquer tree (Mayer and Staples, 2002). Laccases have also been hypothesized to participate in the biosynthesis of protective plant natural products such as neolignans. Recently, a study of *Arabidopsis* gene knockouts showed that an *A. thaliana* laccase, AtLac5, and a dirigent protein, AtDir12, are involved in the biosynthesis of protective *Arabidopsis* seed neolignans (Yonekura-Sakakibara et al., 2020) highlighting the potential role of laccases in biosynthesis of lignan scaffolds. The elucidation of specific plant laccase functions is complicated by their high numbers in plant genomes, their often tissue-specific distribution and their hypothesized catalytic redundancy (Gavnholt et al., 2002; McCaig et al., 2005; Lu et al., 2013; Wang et al., 2015; Balasubramanian et al., 2016; Liu et al., 2017, 2020; Berni et al., 2019; Cheng et al., 2019; Wang Q. et al., 2019; Xu et al., 2019; Arcuri et al., 2020). Characterization of endogenous catalytic functions of plant laccases in plant metabolism, characterization of substrate-determining active site residues and characterization of their temporal and spatial expression profiles will further contribute to understanding their biological functions in plants.

### Ascorbate Oxidases

Ascorbate oxidase is only found in plants and fungi (Hoegger et al., 2006), and is localized along the cell wall in the apoplast, intercellular spaces (De Tullio et al., 2013), and within the vacuole (Liso et al., 2004). Ascorbate oxidase is vital to redox regulation in the extracellular space (Fotopoulos et al., 2006; De Tullio et al., 2013). It reduces the possibility of dioxygen to be transformed into a reactive oxygen species, which is formed by extracellular ascorbate oxidase. Dehydroascorbate can cross the plasma membrane (Pignocchi and Foyer, 2003), where its reduction in the cytosol by the ascorbate-glutathione pathway maintains reactive oxygen species homeostasis (Pignocchi and Foyer, 2003; De Tullio et al., 2013; Pandey et al., 2015).

Ascorbate oxidase is also involved in regulation of plant stress responses and plant growth. Both abiotic and biotic stresses can oxidize ascorbate in the extracellular space. Overexpression of ascorbate oxidase in tobacco increased dehydroascorbate in the apoplast, which led to altered stomatal closure (Fotopoulos et al., 2008). Ascorbate oxidase overexpression in tobacco has also resulted in increased biomass and elongation (Pignocchi et al., 2003; Li et al., 2017) and high ascorbate oxidase activity is found in fast-growing tissues (Lin and Varner, 1991). Herein, dehydroascorbate accumulation leads to cell wall loosening, facilitating growth and elongation (Lin and Varner, 1991; Kato and Esaka, 1999; Li et al., 2017). Ascorbate oxidase expression can be induced in the apoplast by auxin, which is due to auxin-sensitive promotors of ascorbate oxidase genes (Kisu et al., 1997; Pignocchi et al., 2003; Xin et al., 2016).

### Type III Polyphenol Oxidases

Type 3 polyphenol oxidases (Olmedo et al., 2018) contribute to metabolic plant defenses such as during fruit browning response (Zhang and Sun, 2021). Most plant PPOs are nuclear-encoded proteins (Olmedo et al., 2018) and contain signal and transit peptides that direct them to the thylakoid lumen, with some exceptions (Kaintz et al., 2014; Sullivan, 2014; Molitor et al., 2016). Aurone synthases are glycoproteins localized to the vacuole that aid in formation of yellow pigments in the petals of different Asteraceae species, carnations, and snapdragons (Molitor et al., 2015). As for laccases, most endogenous substrates and, thus, endogenous biochemical functions of T3 PPOs remain to be characterized. The broad substrate scope of these enzymes, however, suggests their potential role in biosynthesis of diverse aromatic plant natural products.

### Copper-Containing Amine Oxidases

Copper-containing amine oxidases are either localized to the peroxisome or apoplast (Planas-Portell et al., 2013). They have a prominent role in producing γ-aminobutyric acid (GABA) (Zarei et al., 2015) from putrescine, diamine homeostasis, and alkaloid biosynthesis in the peroxisome (Naconsie et al., 2014). CuAO catalyzes an early step in the biosynthesis of nicotine (Heim et al., 2007; Katoh et al., 2007) and tropane alkaloids (Hashimoto et al., 1990; Pua and Davey, 2010; Kohnen-Johannsen and Kayser, 2019) by the oxidative deamination of *N*-methylputrescine. The biosynthesis of *Lycopodium* alkaloids huperzine A (Sun et al., 2012) and quinolizidine alkaloid biosynthesis (Yang et al., 2017) is initiated by CuAO-mediated transformation of cadaverine to 5-aminopentanal, which spontaneously forms Δ^1^-piperideine, as confirmed by *in vitro* heterologous expression (Lichman, 2021). Based on characterized CuAOs in alkaloid scaffold generation, several alkaloid biosynthetic proposals suggest corresponding roles of CuAOs. For example, a CuAO is proposed to catalyze the transformation of homospermidine to 4-(4-oxobutylamino)butanal in the synthesis of pyrrolizidine alkaloid (Frölich et al., 2007; Schramm et al., 2019; Lichman, 2021).

Peroxisomal CuAO has an important role in grape ripening (Agudelo-Romero et al., 2013) and abscisic acid-induced stomatal closure (An et al., 2008; Planas-Portell et al., 2013; Naconsie et al., 2014; Qu et al., 2014; Fraudentali et al., 2019; Wang W. et al., 2019). The apoplastic CuAOs are implicated in processes such as abiotic and biotic stress responses, wound healing and defense (Rea et al., 2002), cross-linking cell wall components during growth (Tipping and McPherson, 1995), polyamine and abscisic acid-manipulated nitric oxide production (Wimalasekera et al., 2011; Planas-Portell et al., 2013), fruit flavor and flower fragrance biosynthesis (Zarei et al., 2015), and vascular development (Møller and McPherson, 1998; Ghuge et al., 2015). Additional recent work has indicated CuAO is also involved in seed imbibition, when seeds absorb water (Fabrissin et al., 2019).

### Cu,Zn-Superoxide Dismutase

Cu,Zn-SOD catalyzes the disproportionation of superoxide in mitochondria, peroxisome, glyoxysome, cytosol, chloroplast, vacuole and tonoplast, nucleus, and extracellular space (Sandalio and Del Río, 1987; Ogawa et al., 1996, 1997; Corpas et al., 1998; Szöllösi, 2014; Berwal and Ram, 2019; Mishra and Sharma, 2019). Many isoforms of Cu,Zn-SOD are found throughout a plant cell because superoxide and hydrogen peroxide cannot effectively penetrate phospholipid membranes (Takahashi and Asada, 1983; Bienert et al., 2006, 2007) and, thus, it cannot diffuse well between organelles (Huang et al., 2012; Janku et al., 2019). Catalytic superoxide dismutation aids in lignification in the apoplast (Ogawa et al., 1997) and in the prevention of fatal mutations of DNA by superoxide in the nucleus (Ogawa et al., 1996). Cu,Zn-SOD scavenges superoxide in the stroma of the chloroplast, specifically the outer surface of the thylakoid near photosystem I (Pilon et al., 2011). Cytosolic Cu,Zn-SOD is a stress response enzyme, induced under conditions of ozone, UV light exposure (Kliebenstein et al., 1998), and drought (Mittler and Zilinskas, 1994). Overall, Cu,Zn-SOD overexpression in various transgenic plants led to increased tolerance of oxidative stress, high salinity, drought, and cold temperatures (Mishra and Sharma, 2019). The extracellular Cu,Zn-SOD isoform is important for lignification (Ogawa et al., 1997; Kim et al., 2008). Up to 40% of the Cu,Zn-SOD isoforms in spinach leaves are localized to the apoplast rather than cytoplasmic organelles (Ogawa et al., 1997). Hydrogen peroxide is a substrate for class III peroxidases that form lignin, and those peroxidases can be inhibited by superoxide (Ogawa et al., 1997). In plant tissues without lignin formation, like cotton fiber, the extracellular location of Cu,Zn-SOD is proposed to aid in primary and secondary wall biosynthesis (Kim and Triplett, 2008).

### BURP Domain Peptide Cyclases

BURP domain proteins of the four founding member classes (BMN2, RD22, USP, and PG1β) have been investigated in potential roles in plant development and plant responses to abiotic and biotic stress. Several studies identified a role of BURP domain proteins in plant cell elongation and, in particular, cell wall expansion. A corresponding BURP domain protein belonging to the RD22 class has been characterized in cotton, which co-expresses with an expansin protein in the plant cell wall and their overexpression causes increased plant growth and cotton fiber length (Xu et al., 2013). Similarly, a polygalacturonase 1β subunit BURP domain, AtPGL3, has been characterized to promote cell enlargement in *Arabidopsis thaliana* (Park et al., 2015). In addition, BURP domain proteins have been associated with the development of seed coats such as SCB1 in soybean (Batchelor et al., 2002) and AtUSP in *Arabidopsis thaliana* (Van Son et al., 2009), where they localize to protein storage vacuoles. The founding member PG1β from tomato has also been characterized in cell wall rearrangement during fruit ripening (Zheng et al., 1992). Finally, a cereal-specific BURP domain protein called RAFTIN is essential for pollen development in rice and wheat (Wang et al., 2003). Despite the characterized physiological roles of BURP domains in plant growth and development, their functions and underlying biochemistry in these processes remains to be determined.

Several BURP domain proteins were characterized in responses to abiotic and biotic stresses. BURP domain cyclase Sali3-2, which is a lyciumin I precursor in soybean, was discovered as a highly expressed protein in soybean roots during aluminum stress (Tang et al., 2014). Candidate cyclopeptide alkaloid precursor GLYMA_04G180400 is also highly upregulated during salt stress in soybean plants (Zeng et al., 2019). Interestingly, this gene is also highly expressed in soybean plants, that are resistant to soybean mosaic virus infections (Xun et al., 2019). Finally, BURP domain proteins, which are potentially associated with the production of cyclopeptide alkaloids in mung bean strain TC1966 have been identified as candidate resistance genes against azuki bean weevils. A cyclopeptide alkaloid called vignatic acid A, which matched candidate core peptide motifs in the bruchid resistance BURP domain that was isolated from mung bean strain TC1966, showed insecticidal activity against the azuki bean weevil, indicating a potential role of BURP domain peptide cyclases in biotic plant defense (Sugawara et al., 1996). Similar to BURP domain proteins involved in plant development, BURP domain proteins involved in plant stress responses will need to be characterized in their biochemical mechanisms and how their cyclic peptide products help plants withstand biotic and abiotic stresses.

## Copper Enzyme Discovery in Plant Metabolism

Plant copper metalloenzymes represent an interesting area of unexplored plant metabolism for enzyme discovery and metabolic engineering of plant natural products. With growing plant genome sequences, the number of genes encoding cryptic plant copper enzymes is steadily growing. For example, a basic search for genes encoding enzymes of the defined copper enzyme classes laccase, T3 PPO, ascorbate oxidase, CuAO and BURP domain in 46 phylodiverse plant genomes representing 45 plant families in the JGI Phytozome genomic database (Goodstein et al., 2012) was performed by Keyword search of the corresponding E.C. number or ‘BURP domain.’ The query revealed that plants encode on average more than 70 copper metalloenzymes (Table 2). The identified full-length copper enzyme hits – except BURP domain proteins - from each genome were then classified by Muscle alignment with representative protein sequences of each copper enzyme class (see Supporting Information for representative protein list) including the structurally elucidated proteins covered in this review. Subsequent phylogenetic analysis of the aligned copper enzymes by neighbor-joining method classified candidate copper enzymes based on the relationship to characterized representatives and showed that the largest class of copper enzymes are laccases in plant genomes with an average 29 genes per genome, whereas genes of CuAOs and T3 PPOs are found on average six times (Table 2). This smaller number might be due to the role of CuAOs and T3 PPOs in small molecule metabolism compared to the additional role of laccases in cell wall polymer biosynthesis. Ascorbate oxidases appear on average four times in plant genomes, whereas the average number of BURP domain proteins is 12. The gene numbers of each copper enzyme class in genomes can vary significantly between plant genomes, which can be due to evolutionary position, life style, specialized metabolism and genome size of a given plant. For example, genomes of non-vascular plants and aquatic vascular plants have not more than seven laccases whereas terrestrial vascular plants have at least eleven and up to more than seventy laccases encoded in their genomes. This difference in laccase numbers could be due to their importance in development of cell walls and, therefore, support of vascular tissues. In addition, some classes of copper enzymes might be more represented in some plant families due to gene family expansion during evolution of beneficial metabolic traits (Weng et al., 2012). Interestingly, there is a well-supported clade of undefined multi-copper oxidases (average number of 17/genome) in most analyzed plant genomes, indicating the prospect of uncharacterized classes of multi-copper oxidases in plants (Table 2 and Supporting Information). The characterization of the BURP domain as a new class of copper-dependent plant peptide cyclase highlights that copper enzymes with new catalyzed reactions and metabolic products are hidden in plant genomes (Chigumba et al., 2021). A starting point for functional prediction of a plant copper enzyme can be the identification of signal and transit peptides in its sequence, which indicate the enzyme localization in the plant cell such as apoplast or vacuole via signal peptides or the thylakoid lumen via transit peptides. A hypothetical localization can then inform compartment-specific substrates and reaction conditions such as pH for a copper enzyme. Despite the presented understanding of the structural basis of copper plant metalloenzymes, a general bottleneck in their functional classification from genetic sequences is the prediction of catalyzed reactions of copper enzymes in plant metabolism because endogenous metabolic substrates have only been characterized for a few plant copper enzymes (Nakayama et al., 2000). In order to fully realize the biochemical roles of plant copper enzymes in plant metabolism and, in specific, in plant natural product biosynthesis, more plant copper enzymes have to be characterized in the context of their biosynthetic pathways, i.e., their endogenous substrates and catalyzed reactions.

**TABLE 2:** Genome mining of plant copper metalloenzymes.

Species (genome version - JGI Phytozome 13)	Family	Clade	Copper amine oxidases (EC 1.4.3.21)	T3 polyphenol oxidases (EC 1.10.3.1, 1.14.18.1, 1.21.3.6)	Laccases (EC 1.10.3.2)	Ascorbate oxidases (EC 1.10.3.3)	BURP domain proteins	Undefined multi-copper oxidases	Total copper enzyme genes
*Chlamydomonas reinhardtii (v5.6)*	Chlamydomonadaceae	Chlorophyta	2	0	0	0	0	7	9
*Marchantia polymorpha (v3.1)*	Marchantiaceae	Marchantiophyta	2	17	1	3	1	34	58
*Physcomitrium patens (v3.3)*	Funariaceae	Bryophyta	7	9	5	5	5	1	32
*Sphagnum fallax (v1.1)*	Sphagnaceae	Bryophyta	3	5	6	4	6	0	24
*Selaginella moellendorffii (v1.0)*	Selaginellaceae	Lycophytes	2	3	11	1	6	4	27
*Ceratopteris richardii (v2.1)*	Pteridaceae	Polypodiophyta	3	45	32	2	11	10	103
*Thuja plicata (v3.1)*	Cupressaceae	Pinophyta	4	4	62	9	14	10	103
*Amborella trichopoda (v1.0)*	Amborellaceae	Early angiosperms	2	0	13	3	10	6	34
*Nymphaea colorata (v1.2)*	Nymphaeaceae	Early angiosperms	2	0	31	3	25	11	72
*Cinnamomum kanehirae (v3)*	Lauraceae	Magnoliids	8	6	22	4	6	15	61
*Acorus americanus (v1.1)*	Acoraceae	Monocots	5	4	26	3	17	15	70
*Ananas comosus (v3)*	Bromeliaceae	Monocots	5	4	17	2	4	13	45
*Asparagus officinalis (v1.1)*	Asparagaceae	Monocots	3	0	12	1	5	10	31
*Dioscorea alata (v2.1)*	Dioscoreaceae	Monocots	5	11	19	2	10	12	59
*Musa acuminata (v1)*	Musaceae	Monocots	3	8	20	3	7	25	66
*Joinvillea ascendens (v1.1)*	Joinvilleaceae	Monocots	2	4	33	3	9	7	58
*Spirodela polyrhiza (v2)*	Araceae	Monocots	1	6	7	3	9	8	34
*Zostera marina (v3.1)*	Zosteraceae	Monocots	2	6	3	3	7	10	31
*Sorghum bicolor (v3.1.1)*	Poaceae	Monocots	4	8	26	6	10	10	64
*Zea mays (RefGen_V4)*	Poaceae	Monocots	3	6	21	4	9	13	56
*Amaranthus hypochondriacus (v2.1)*	Amaranthaceae	Eudicots	2	5	14	3	15	16	55
*Kalanchoe fedtschenkoi (v1.1)*	Crassulaceae	Eudicots	4	3	18	4	25	24	78
*Coffea arabica (v0.5)*	Rubiaceae	Eudicots	9	16	52	9	53	23	162
*Daucus carota (v2.0)*	Apiaceae	Eudicots	5	5	23	5	11	22	71
*Helianthus annuus (r1.2)*	Asteraceae	Eudicots	7	11	53	3	19	20	113
*Hydrangea quercifolia (v1.1)*	Hydrangeaceae	Eudicots	10	5	25	3	8	19	70
*Aquilegia coerulea (v3.1)*	Ranunculaceae	Eudicots	7	6	25	3	5	15	61
*Lactuca sativa (v8)*	Asteraceae	Eudicots	3	18	32	3	12	20	88
*Mimulus guttatus (TOL v5.0)*	Phrymaceae	Eudicots	4	6	19	2	8	19	58
*Olea europaea (v1.0)*	Oleaceae	Eudicots	7	12	34	2	11	22	88
*Solanum lycopersicum (ITAG3.2)*	Solanaceae	Eudicots	4	8	26	3	14	19	74
*Eucalyptus grandis (v2.0)*	Myrtaceae	Eudicots	8	3	74	9	14	17	125
*Vitis vinifera (v2.1)*	Vitaceae	Eudicots	2	1	70	5	26	12	116
*Carya illinoinensis (v1.1)*	Juglandaceae	Eudicots	9	2	44	7	9	17	88
*Cucumis sativus (v1.0)*	Cucurbitaceae	Eudicots	6	1	14	4	6	17	48
*Glycine max (Wm82.a4.v1)*	Fabaceae	Eudicots	13	15	51	5	22	29	135
*Malus domestica (v1.1)*	Rosaceae	Eudicots	8	15	49	4	15	25	116
*Corymbia citriodora (v2.1)*	Myrtaceae	Eudicots	7	1	59	10	11	19	107
*Linum usitatissimum (v1.0)*	Linaceae	Eudicots	9	10	42	6	16	33	116
*Manihot esculenta (v7.1)*	Euphorbiaceae	Eudicots	8	1	29	3	34	23	98
*Populus trichocarpa (v4.1)*	Salicaceae	Eudicots	8	12	49	7	13	19	108
*Anacardium occidentale (v0.9)*	Anacardiaceae	Eudicots	10	6	61	7	11	21	116
*Carica papaya (ASGPBv0.4)*	Carica papaya	Eudicots	6	6	14	1	10	12	49
*Theobroma cacao (v2.1)*	Malvaceae	Eudicots	7	6	27	5	11	15	71
*Arabidopsis thaliana (TAIR10)*	Brassicaceae	Eudicots	8	0	17	3	5	19	52
*Brassica rapa (FPsc v1.3)*	Brassicaceae	Eudicots	5	1	24	6	9	39	84
*Citrus sinensis (v1.1)*	Rutaceae	Eudicots	7	1	21	2	6	12	49
		Average	6	6	29	4	12	17	74

*JGI Phytozome 13 (Goodstein et al., 2012) was searched for genes encoding E.C. 1.4.3.21, 1.10.3.1, 1.14.18.1, 1.21.3.6, and 1.10.3.3 via Keyword search. Protein sequences of identified genes were filtered for full-length genes, aligned with sequences of proteins representative of CuAOs, T3 PPOs, laccases and ascorbate oxidases by muscle (Edgar, 2004), and a neighbor-joining phylogenetic tree (2000 bootstraps) (Saitou and Nei, 1987) was generated to predict the numbers of copper enzymes of respective enzyme classes in each plant genome with MEGA-X (Kumar et al., 2018). For all genomes, a clade of copper enzymes remained and was assigned as unidentified. For phylogenetic trees, please see Supplementary Information.*

**FIGURE 9:**
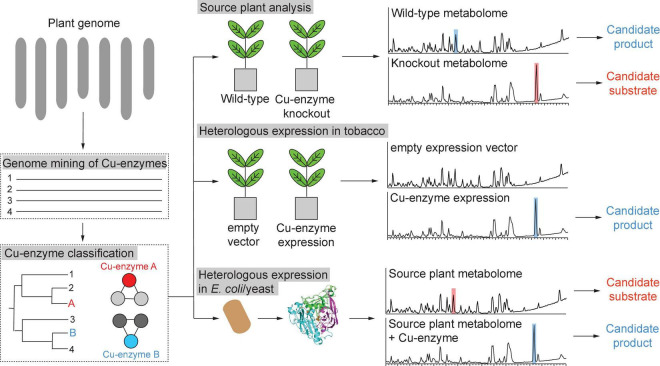
Strategies for characterization of copper metalloenzymes from plant genomes.

There are several strategies for copper enzyme characterization (Figure 9). The first strategy is the knockout of a target copper enzyme in a source plant and subsequent metabolomic identification of candidate substrates and products by comparative metabolomics of wild-type and Cu-enzyme mutant plant. Herein, an important example is the characterization of *Arabidopsis thaliana* laccase 5 in neolignan biosynthesis (Yonekura-Sakakibara et al., 2020). In this study, a laccase was elucidated as a metabolic enzyme in seed protective neolignan biosynthesis by differential gene expression analysis with known lignan-defining dirigent proteins and laccase 5 role in neolignan biosynthesis in *A. thaliana* seeds was established by comparative metabolomic analysis of laccase-5-dirigent-protein mutant plant seeds and wild-type plant seeds. Another example of discovery of candidate new plant copper enzymology via source plant knockout studies is the identification of a putative rhamnogalacturonan galactose oxidase involved in rhamnogalacturonan biosynthesis through *Arabidopsis* mutagenesis. As this type of copper enzyme has not been described in plant metabolism yet, this study highlights the potential for hidden copper biochemistry in plants (Šola et al., 2019). An alternative second strategy to studying metabolic roles of copper enzymes via genetic manipulation of source plants is the heterologous expression of target enzymes in a suitable host organism, which can provide endogenous substrates in the context of their biosynthetic pathways, and subsequent comparative metabolic profiling of copper-enzyme-expressing versus non-expression host tissue (Figure 9). A powerful tool for such pathway reconstitution experiments is transient gene expression in *Nicotiana benthamiana* (Sainsbury et al., 2009; Lau and Sattely, 2015). The reconstitution of copper plant enzymes in a model plant such as *N. benthamiana* also requires the absence of a similar copper enzyme in the host. An example of characterization of new copper enzymes via heterologous expression in tobacco is the identification of BURP domain cyclases (Chigumba et al., 2021). Herein, the BURP domain cyclases provided their endogenous substrate in the form of their core peptide motifs so that the heterologous host only needed to provide processing enzymes to proteolytically cleave the modified core peptides from the BURP domain proteins. For copper enzymes, which do not encode their substrates, the heterologous host tobacco has to provide the substrate or, if the context of the metabolic pathway of the target copper enzyme is known, enzymes from previous pathway steps should be co-expressed with the target enzyme (Schultz et al., 2019). A third strategy is the heterologous expression of target copper enzymes in a bacterial or yeast host, enzyme purification and *in vitro* substrate screening by reconstitution of purified copper enzymes with their required cofactor and a source plant metabolome (Figure 9). An advantage of this strategy is less interference from host enzymes during metabolic profiling of enzyme assays versus control experiments with inactive enzyme, whereas a disadvantage is the purification of an active plant enzyme in a non-native host, which can cause significant problems in case of disulfide-bond formation and glycosylation. In addition, any candidate signal and transit peptides will have to be truncated for purification in a non-plant host. All three strategies for copper enzyme analysis are based on metabolomic identification of candidate substrates and products from complex metabolomic samples. Improvements in mass spectrometry data analysis for untargeted metabolomics make the identification of candidate metabolites and their *de novo* structure prediction from tandem mass spectrometry (MS/MS) data feasible (Pluskal et al., 2010; Dührkop et al., 2019). The growth in MS/MS databases will increase the identification rate of target copper enzyme substrates and respective products, while improvement in NMR analysis and applications of new techniques such as MicroED will enable rapid structure elucidation of copper enzyme metabolites (Wang et al., 2016; Jones et al., 2018; Beniddir et al., 2021).

With an increasing synthetic biology toolkit for the manipulation of target gene expression in non-model plants and improving untargeted metabolomics tools for the characterization of metabolic changes related to the changed expression of a copper enzyme gene in a plant (Smith et al., 2005; Horai et al., 2010; Sawada et al., 2012; Wang et al., 2016; Fenaille et al., 2017), the elucidation of copper enzyme catalysis in their endogenous pathways will improve differentiation of plant copper enzymology and enable application of these copper enzymes in metabolic engineering (Srinivasan and Smolke, 2020). In addition, the dissection of mechanistic determinants in copper enzyme sequences such as monophenolase-determining residues in type III polyphenol oxidases will allow for improved functional prediction of copper enzyme genes in plant genomes (Kampatsikas et al., 2020). Ultimately, the endogenous catalytic capacity of known copper enzymes classes and the discovery of new copper enzymes will broaden our understanding of how plants utilize copper to control the cellular redox state and produce structural biopolymers, defense compounds and other specialized metabolites to withstand oxidative, abiotic and biotic stresses as oxygen-producing sessile organisms.

## Author Contributions

RK designed the review. RK and LM wrote the manuscript. DC designed and performed the phylogenetic analyses. RK, LM, and DC have reviewed and approved the final version of the manuscript. All authors contributed to the article and approved the submitted version.

## Conflict of Interest

The authors declare that the research was conducted in the absence of any commercial or financial relationships that could be construed as a potential conflict of interest.

## Publisher’s Note

All claims expressed in this article are solely those of the authors and do not necessarily represent those of their affiliated organizations, or those of the publisher, the editors and the reviewers. Any product that may be evaluated in this article, or claim that may be made by its manufacturer, is not guaranteed or endorsed by the publisher.
