# Supplementary material for: Plant Copper Metalloenzymes As Prospects for New Metabolism Involving Aromatic Compounds
Source: Front Plant Sci. 2021 Nov 29;12:692108. doi: 10.3389/fpls.2021.692108 (PMC8672867; doi:10.3389/fpls.2021.692108)
Supplement: Supplementary file 1 [file Data_Sheet_1.PDF]

Supplementary Information

Plant Copper Metalloenzymes as Prospects for New Metabolism Involving Aromatic Compounds

Lisa S. Mydy[1], Desnor N. Chigumba[1], Roland D. Kersten[1*]

[1]Department of Medicinal Chemistry, University of Michigan, Ann Arbor, MI, USA

*** Correspondence:**
Roland D. Kersten
rkersten@med.umich.edu

Phylogenetic analyses of genome-derived copper enzyme genes with characterized copper enzyme genes (Table 2). The evolutionary history was inferred using the Neighbor-Joining method [1]. The optimal trees are shown. The percentage of replicate trees in which the associated taxa clustered together in the bootstrap test (2000 replicates) are shown next to the branches [2]. The trees are drawn to scale, with branch lengths in the same units as those of the evolutionary distances used to infer the phylogenetic trees. The evolutionary distances were computed using the p-distance method [3] and are in the units of the number of amino acid differences per site. All ambiguous positions were removed for each sequence pair (pairwise deletion option). Evolutionary analyses were conducted in MEGA X [4].

1. Saitou N. and Nei M. (1987). The neighbor-joining method: A new method for reconstructing phylogenetic trees. Molecular Biology and Evolution 4:406-425.

2. Felsenstein J. (1985). Confidence limits on phylogenies: An approach using the bootstrap. Evolution 39:783-791.

3. Nei M. and Kumar S. (2000). Molecular Evolution and Phylogenetics. Oxford University Press, New York.

4. Kumar S., Stecher G., Li M., Knyaz C., and Tamura K. (2018). MEGA X: Molecular Evolutionary Genetics Analysis across computing platforms. Molecular Biology and Evolution 35:1547-1549.

Characterized copper enzymes: CAJ30499.1-laccase, AHN09736.1-catechol-oxidase, ACN86310.1-tyrosinase, XP_023531516.1-L-ascorbate-oxidase, NP_001312728.1-copper-methylamine-oxidase, AAQ67412.1-larreatricin-hydroxylase, Q9FRX6-Aureusidin-synthase-Antirrhinum-majus, Q5ENY2-Catechol-oxidase-Ipomoea-batatas, AT1G18140-AtLaccase, At2g29130-Atlaccase, At2g30210-Atlaccase, At2g40370-Atlaccase, At2g46570-Atlaccase, At3g09220-Atlaccase, At5g01040-Atlaccase, At5g01050-Atlaccase, At5g01190-Atlaccase, At5g03260-Atlaccase, At5g05390-Atlaccase, At5g07130-Atlaccase, At5g09360-Atlaccase, At5g48100-Atlaccase, LAC16_ARATH Laccase-16, LAC17_ARATH Laccase-17, BNM2A_BRANA, EA87_VICFA-USPL, RD22_ARATH, GP1_SOLLC-PG1b

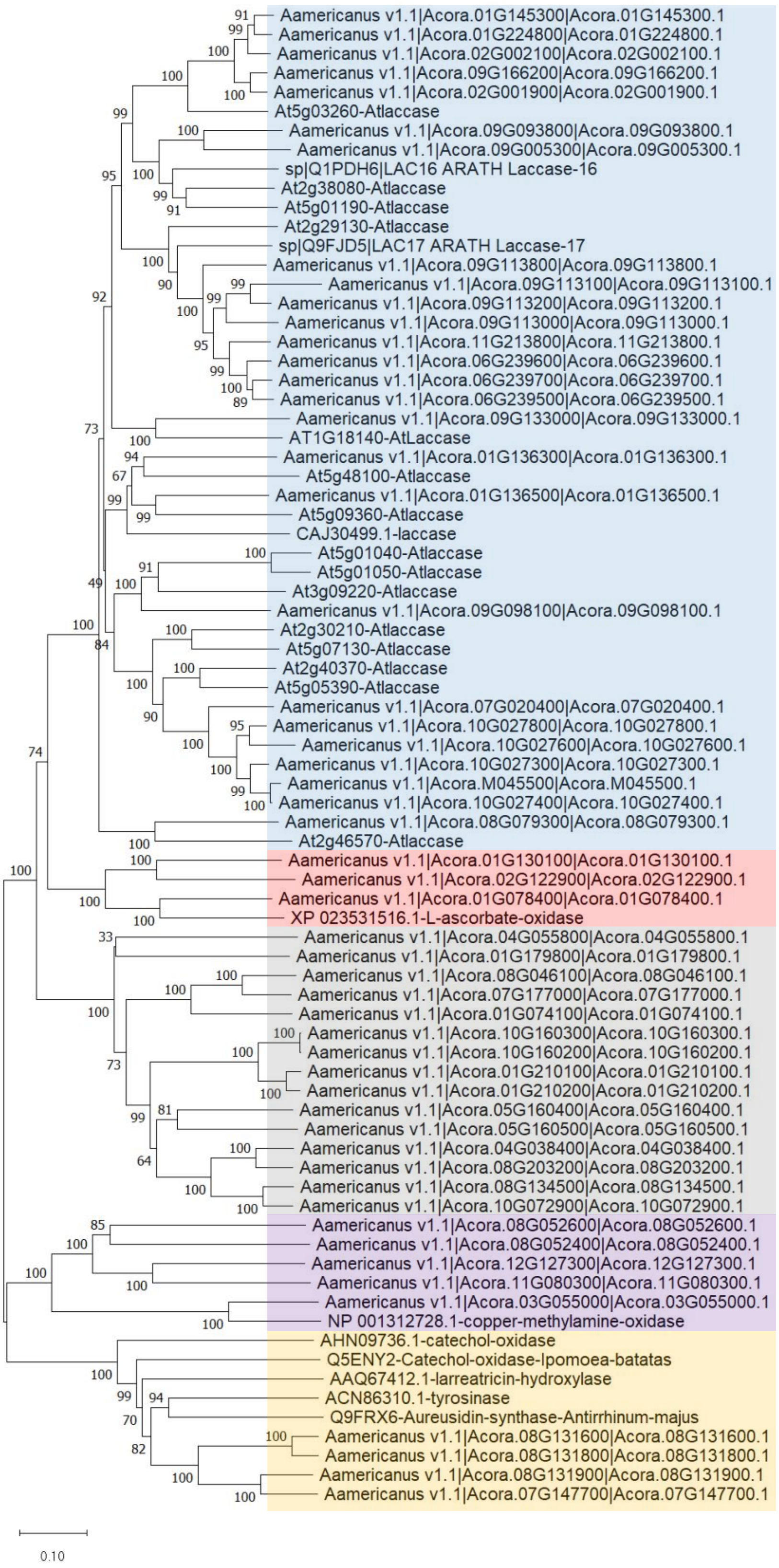

Figure S1 | Phylogenetic analyses of genome-derived copper enzyme genes with characterized copper enzyme genes. *Acorus americanus* (v1.1). Laccases highlighted in blue, ascorbate oxidases highlighted in red, undefined copper enzymes highlighted in grey, copper-methylamine-oxidases highlighted in purple, T3 polyphenol oxidases highlighted in orange.

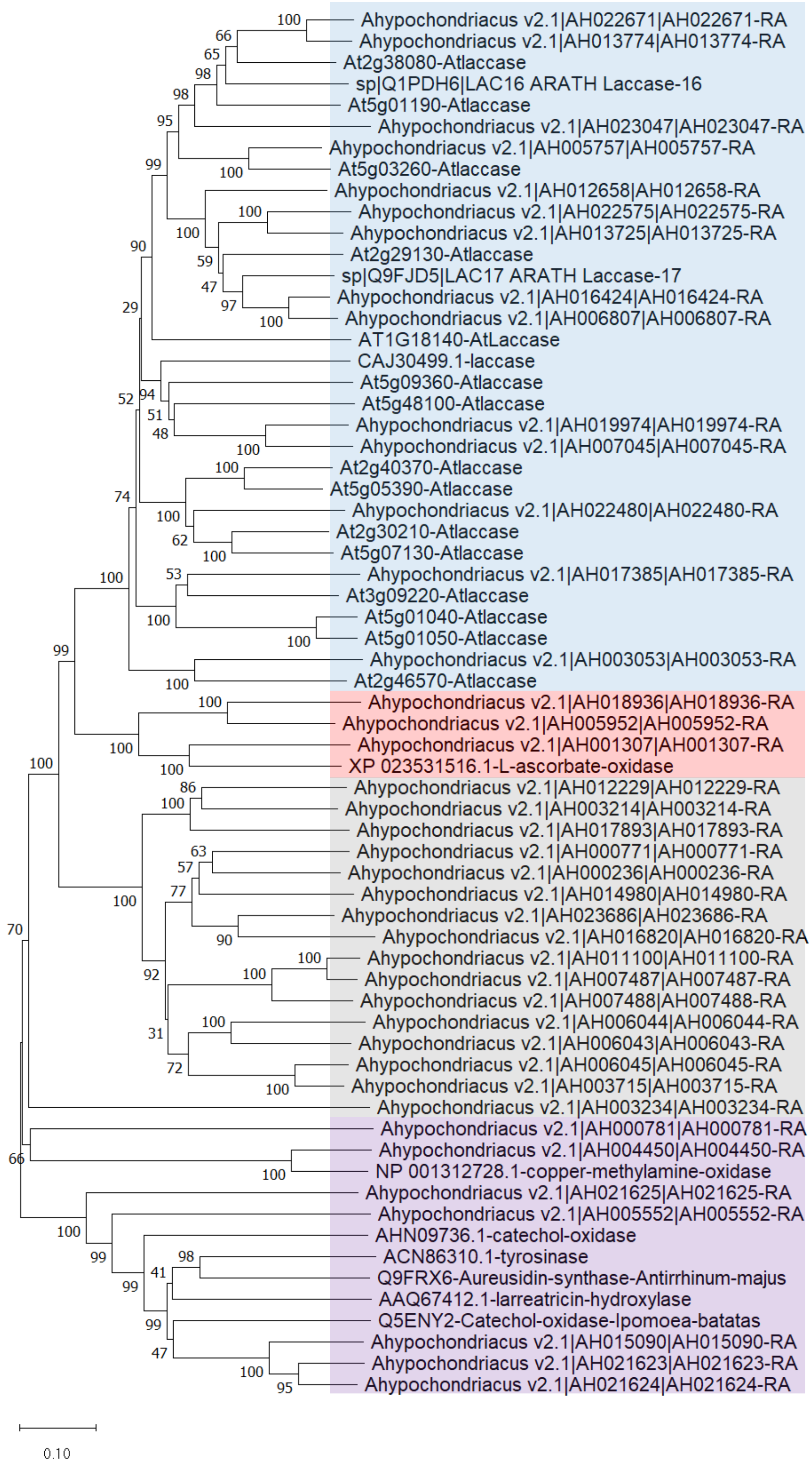

Figure S1 | Phylogenetic analyses of genome-derived copper enzyme genes with characterized copper enzyme genes. *Amaranthus hypochondriacus* (v2.1). Laccases highlighted in blue, ascorbate oxidases highlighted in red, undefined copper enzymes highlighted in grey, copper-methylamine-oxidases highlighted in purple, T3 polyphenol oxidases highlighted in orange.

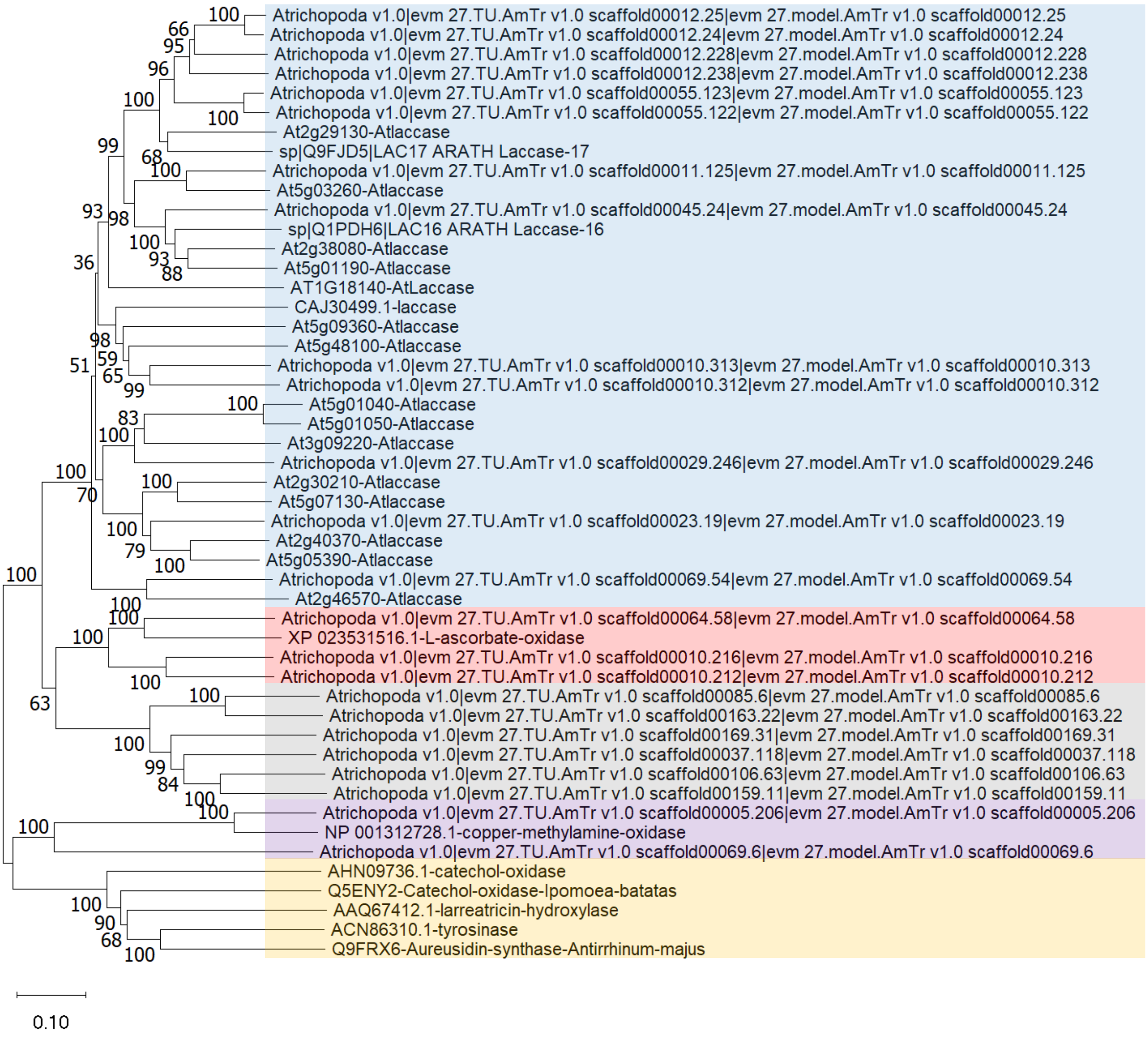

Figure S1 | Phylogenetic analyses of genome-derived copper enzyme genes with characterized copper enzyme genes. *Amborella trichopoda (v1.0).* Laccases highlighted in blue, ascorbate oxidases highlighted in red, undefined copper enzymes highlighted in grey, copper-methylamine-oxidases highlighted in purple, T3 polyphenol oxidases highlighted in orange.

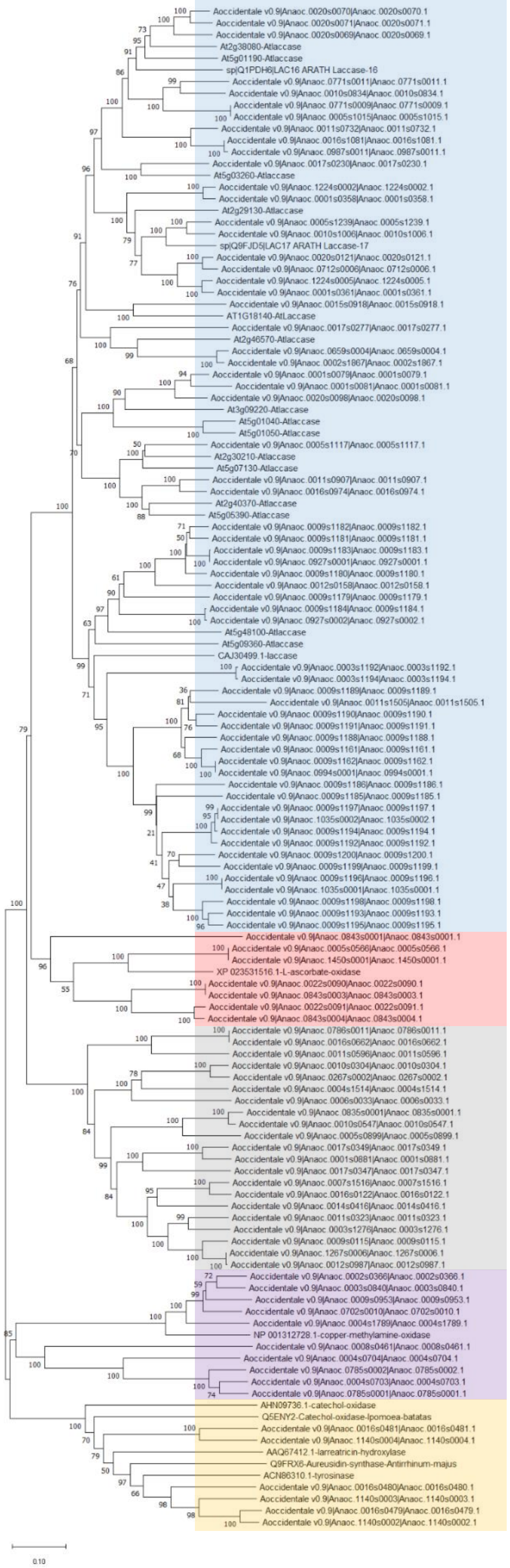

Figure S1 | Phylogenetic analyses of genome-derived copper enzyme genes with characterized copper enzyme genes. *Anacardium occidentale (v0.9).* Laccases highlighted in blue, ascorbate oxidases highlighted in red, undefined copper enzymes highlighted in grey, copper-methylamine-oxidases highlighted in purple, T3 polyphenol oxidases highlighted in orange.

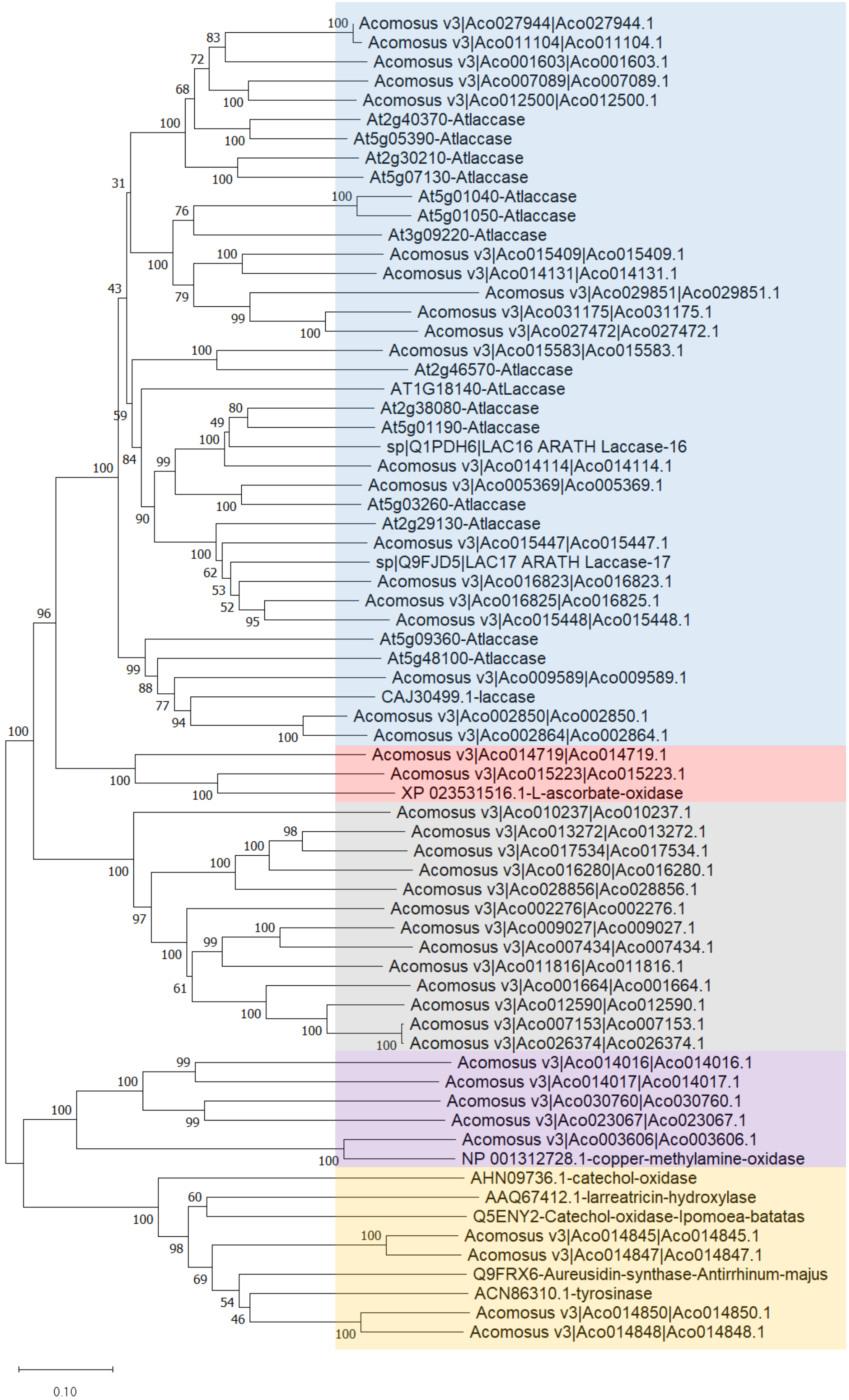

Figure S1 | Phylogenetic analyses of genome-derived copper enzyme genes with characterized copper enzyme genes. *Ananas comosus (v3).* Laccases highlighted in blue, ascorbate oxidases highlighted in red, undefined copper enzymes highlighted in grey, copper-methylamine-oxidases highlighted in purple, T3 polyphenol oxidases highlighted in orange.

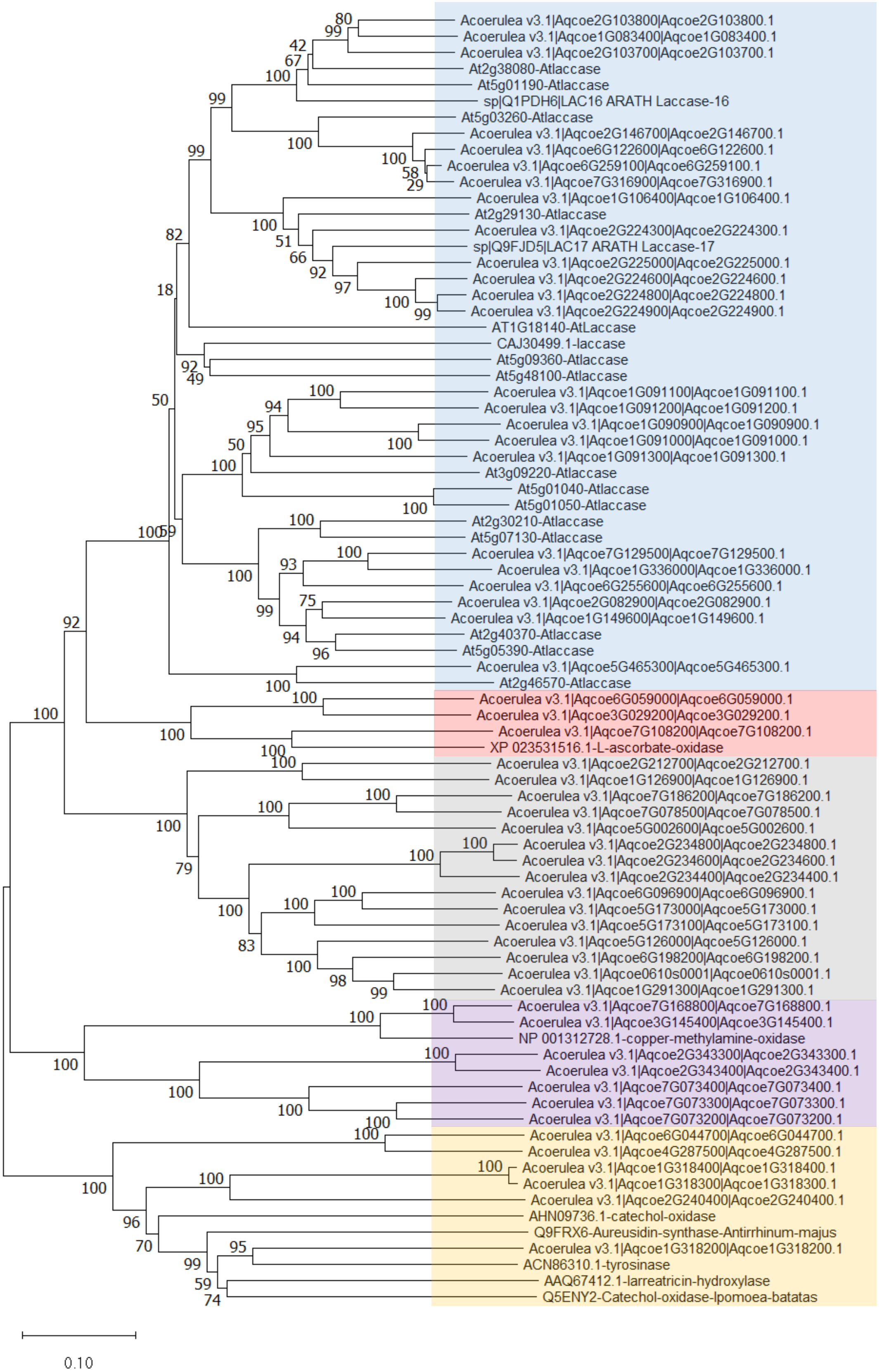

Figure S1 | Phylogenetic analyses of genome-derived copper enzyme genes with characterized copper enzyme genes. *Aquilegia coerulea (v3.1).* Laccases highlighted in blue, ascorbate oxidases highlighted in red, undefined copper enzymes highlighted in grey, copper-methylamine-oxidases highlighted in purple, T3 polyphenol oxidases highlighted in orange.

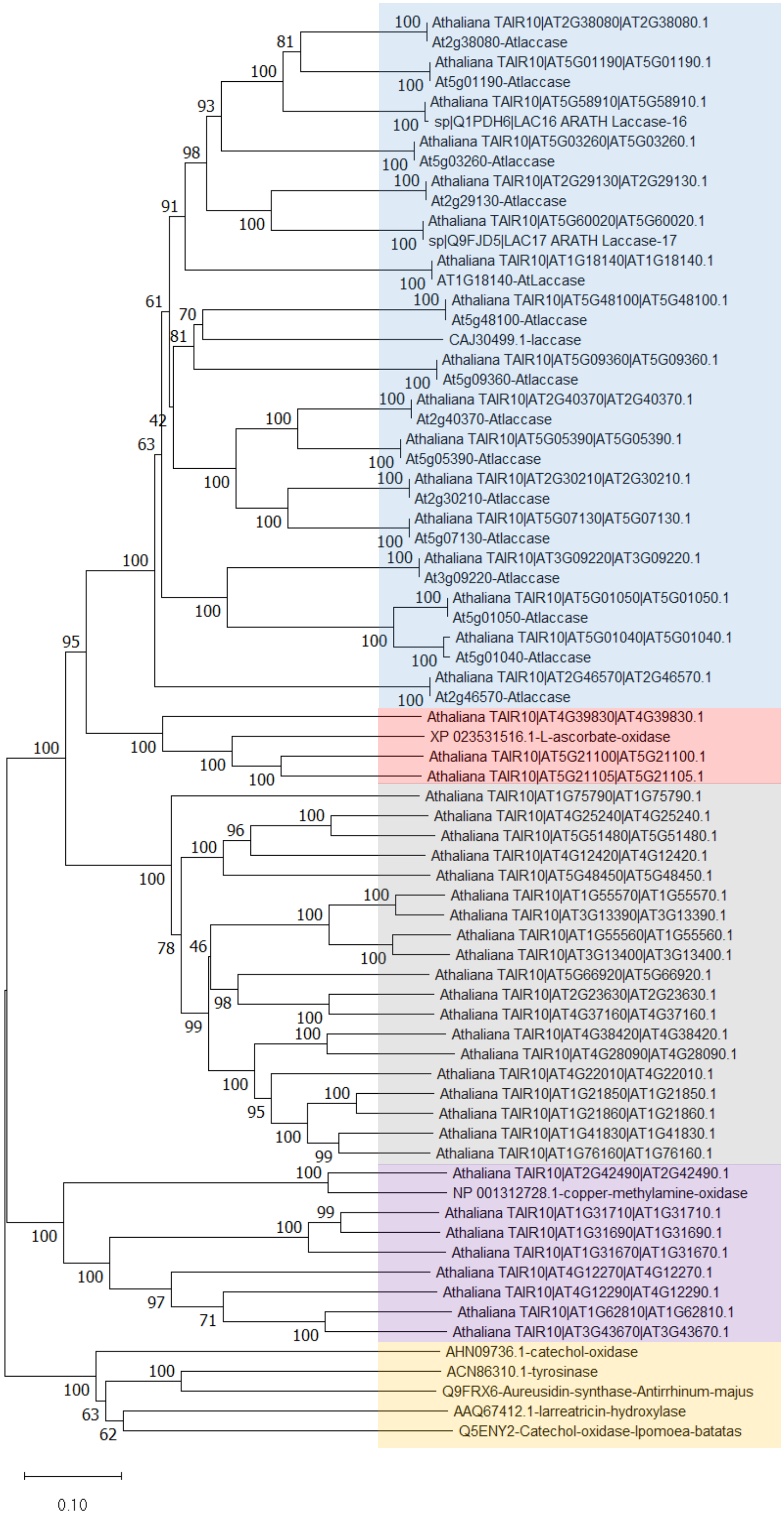

Figure S1 | Phylogenetic analyses of genome-derived copper enzyme genes with characterized copper enzyme genes. *Arabidopsis thaliana (TAIR10).* Laccases highlighted in blue, ascorbate oxidases highlighted in red, undefined copper enzymes highlighted in grey, copper-methylamine-oxidases highlighted in purple, T3 polyphenol oxidases highlighted in orange.

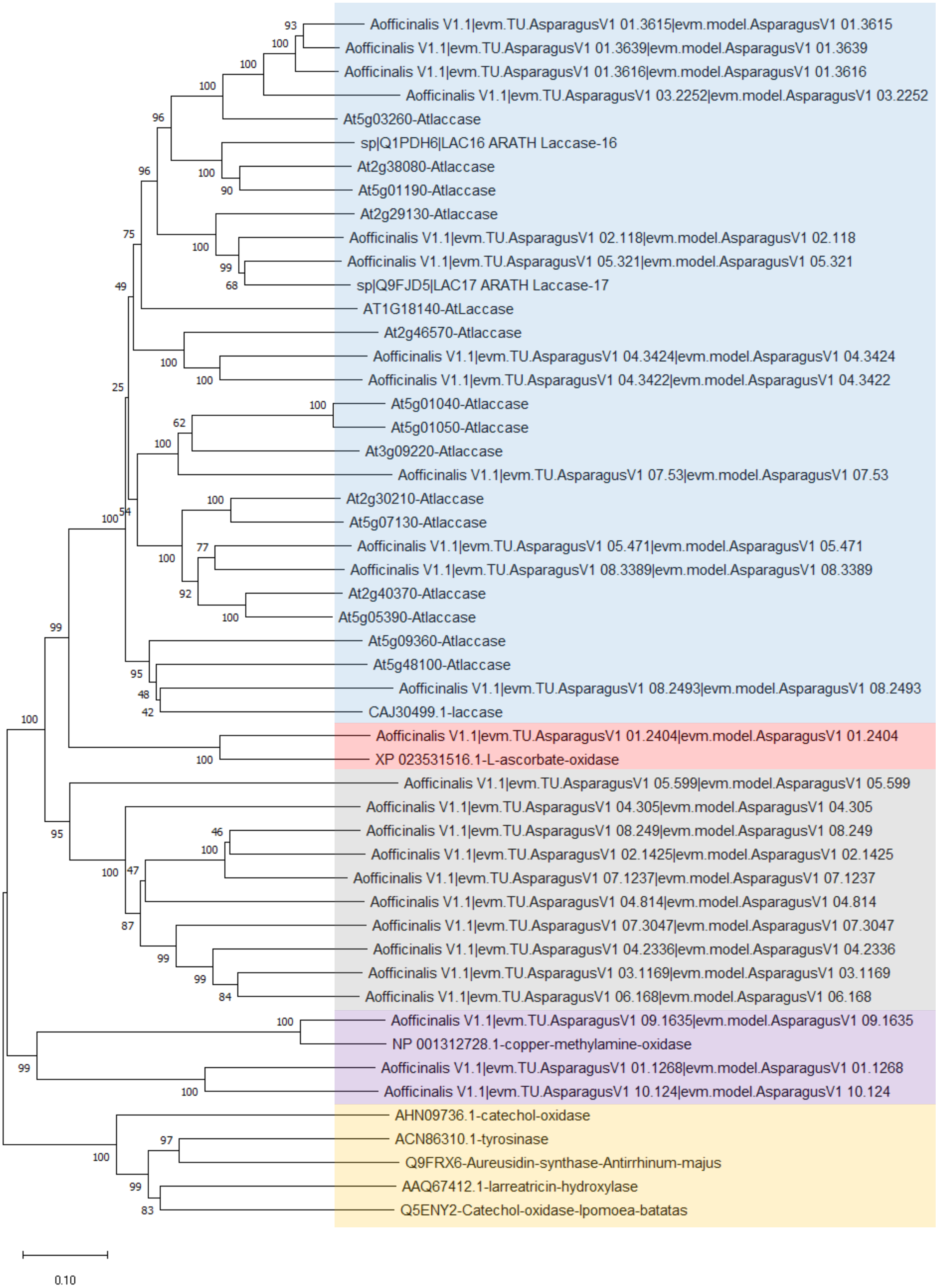

Figure S1 | Phylogenetic analyses of genome-derived copper enzyme genes with characterized copper enzyme genes. *Asparagus officinalis (v1.1).* Laccases highlighted in blue, ascorbate oxidases highlighted in red, undefined copper enzymes highlighted in grey, copper-methylamine-oxidases highlighted in purple, T3 polyphenol oxidases highlighted in orange.

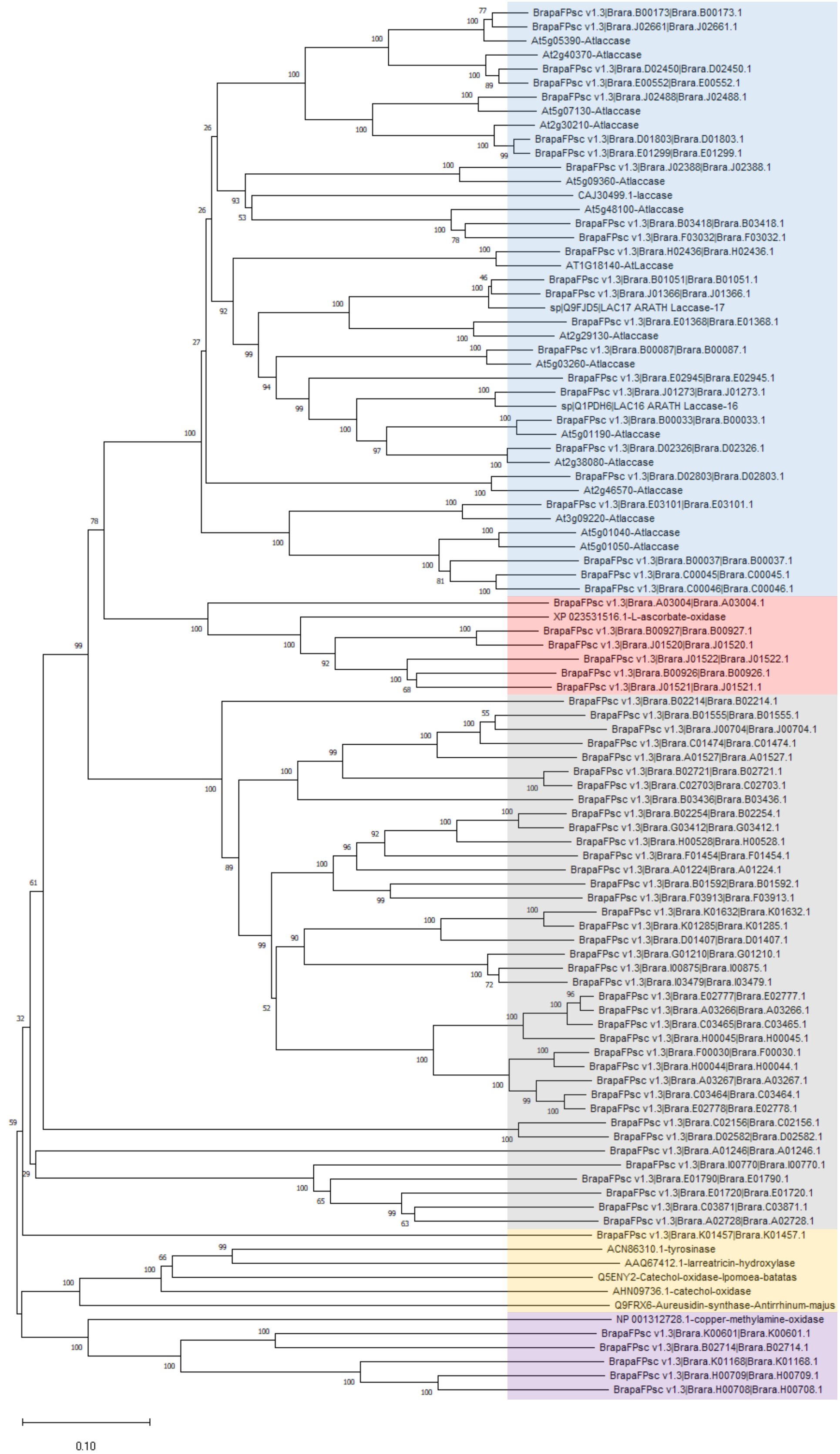

Figure S1 | Phylogenetic analyses of genome-derived copper enzyme genes with characterized copper enzyme genes. *Asparagus officinalis (v1.1).* Laccases highlighted in blue, ascorbate oxidases highlighted in red, undefined copper enzymes highlighted in grey, copper-methylamine-oxidases highlighted in purple, T3 polyphenol oxidases highlighted in orange.

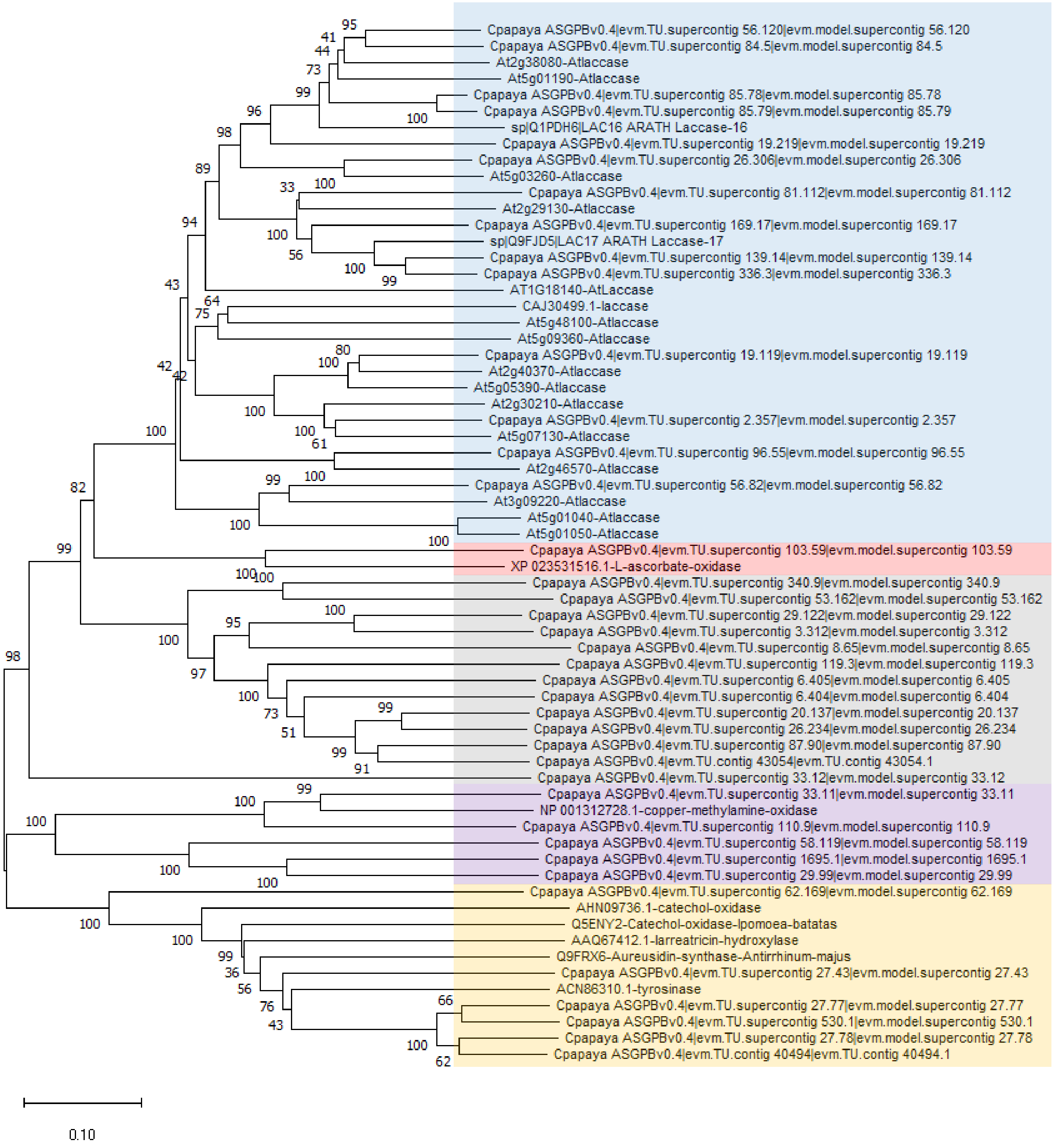

Figure S1 | Phylogenetic analyses of genome-derived copper enzyme genes with characterized copper enzyme genes. *Asparagus officinalis (v1.1).* Laccases highlighted in blue, ascorbate oxidases highlighted in red, undefined copper enzymes highlighted in grey, copper-methylamine-oxidases highlighted in purple, T3 polyphenol oxidases highlighted in orange.

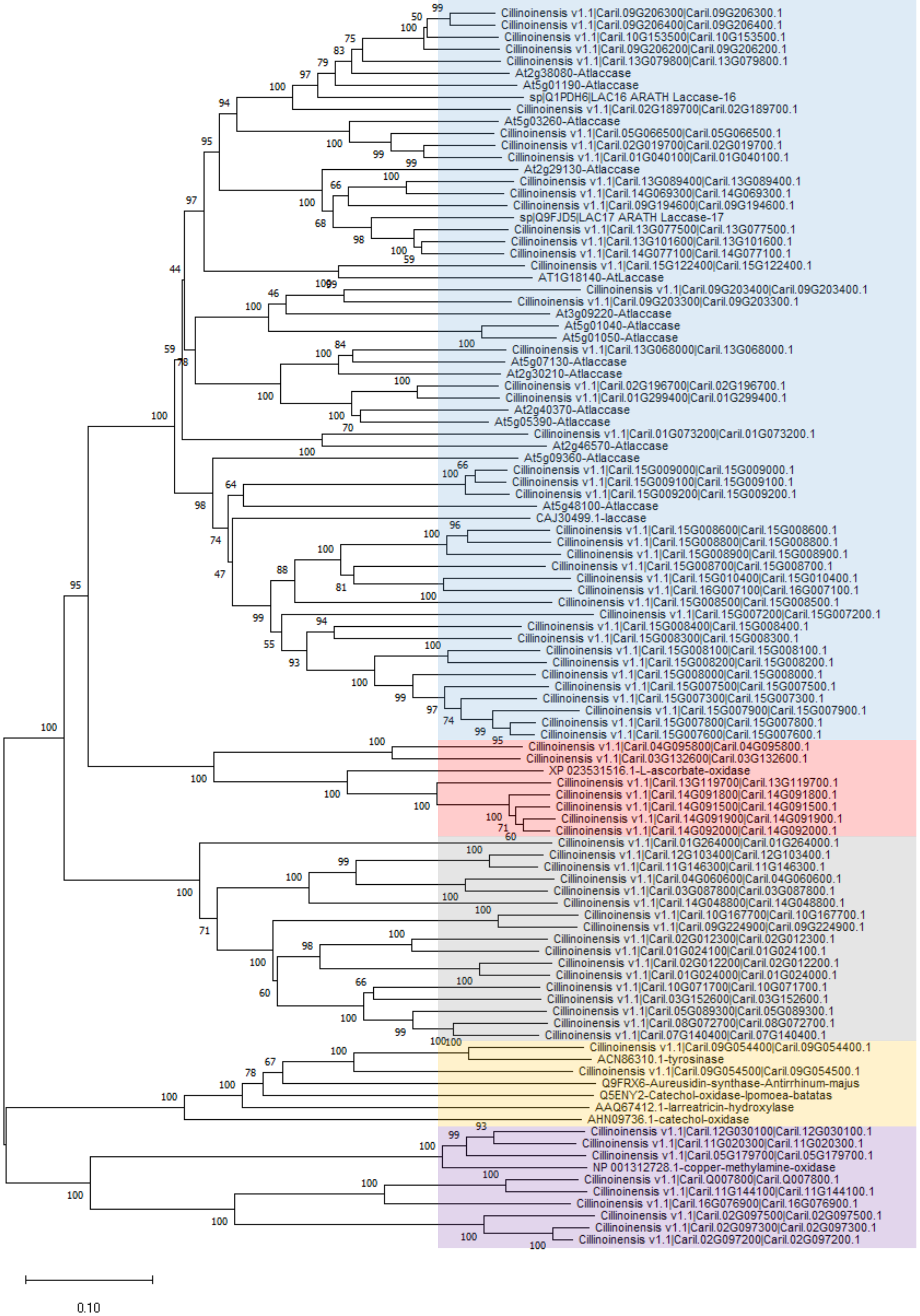

Figure S1 | Phylogenetic analyses of genome-derived copper enzyme genes with characterized copper enzyme genes. *Carya illinoinensis (v1.1).* Laccases highlighted in blue, ascorbate oxidases highlighted in red, undefined copper enzymes highlighted in grey, copper-methylamine-oxidases highlighted in purple, T3 polyphenol oxidases highlighted in orange.

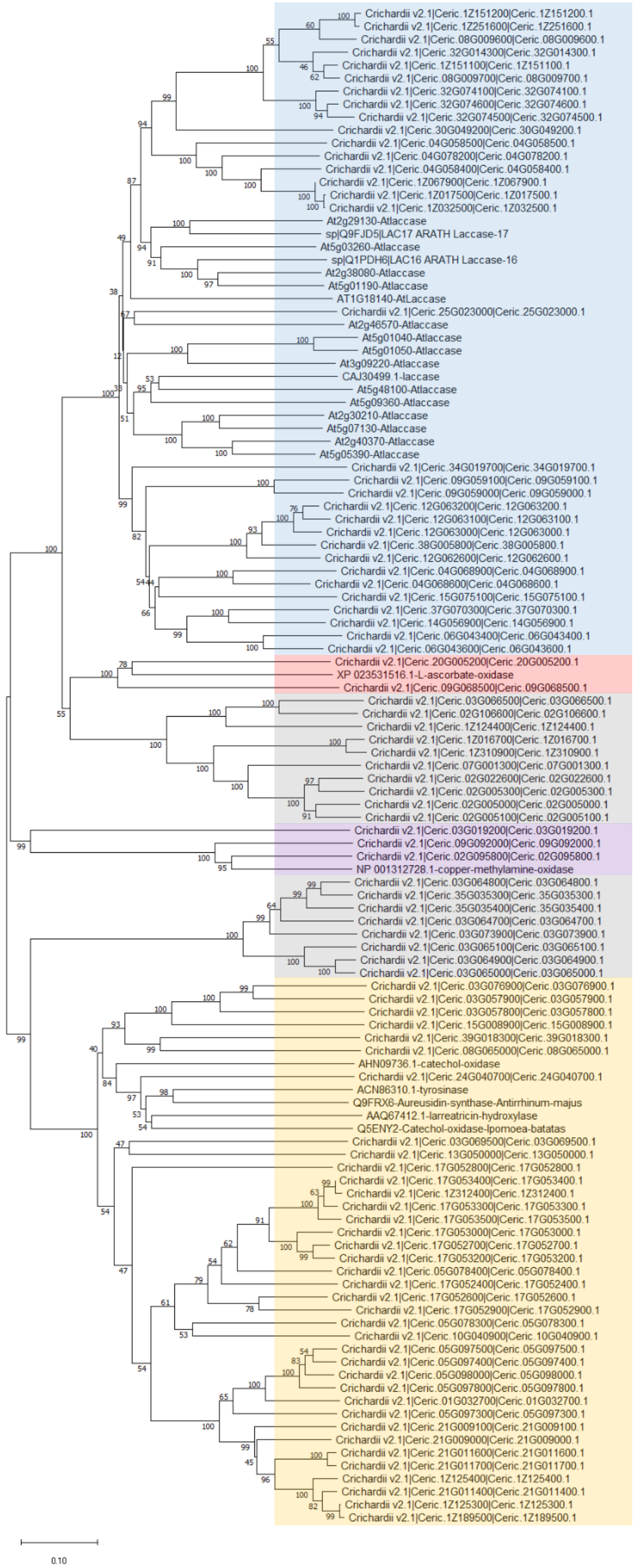

Figure S1 | Phylogenetic analyses of genome-derived copper enzyme genes with characterized copper enzyme genes. *Ceratopteris richardii (v2.1).* Laccases highlighted in blue, ascorbate oxidases highlighted in red, undefined copper enzymes highlighted in grey, copper-methylamine-oxidases highlighted in purple, T3 polyphenol oxidases highlighted in orange.

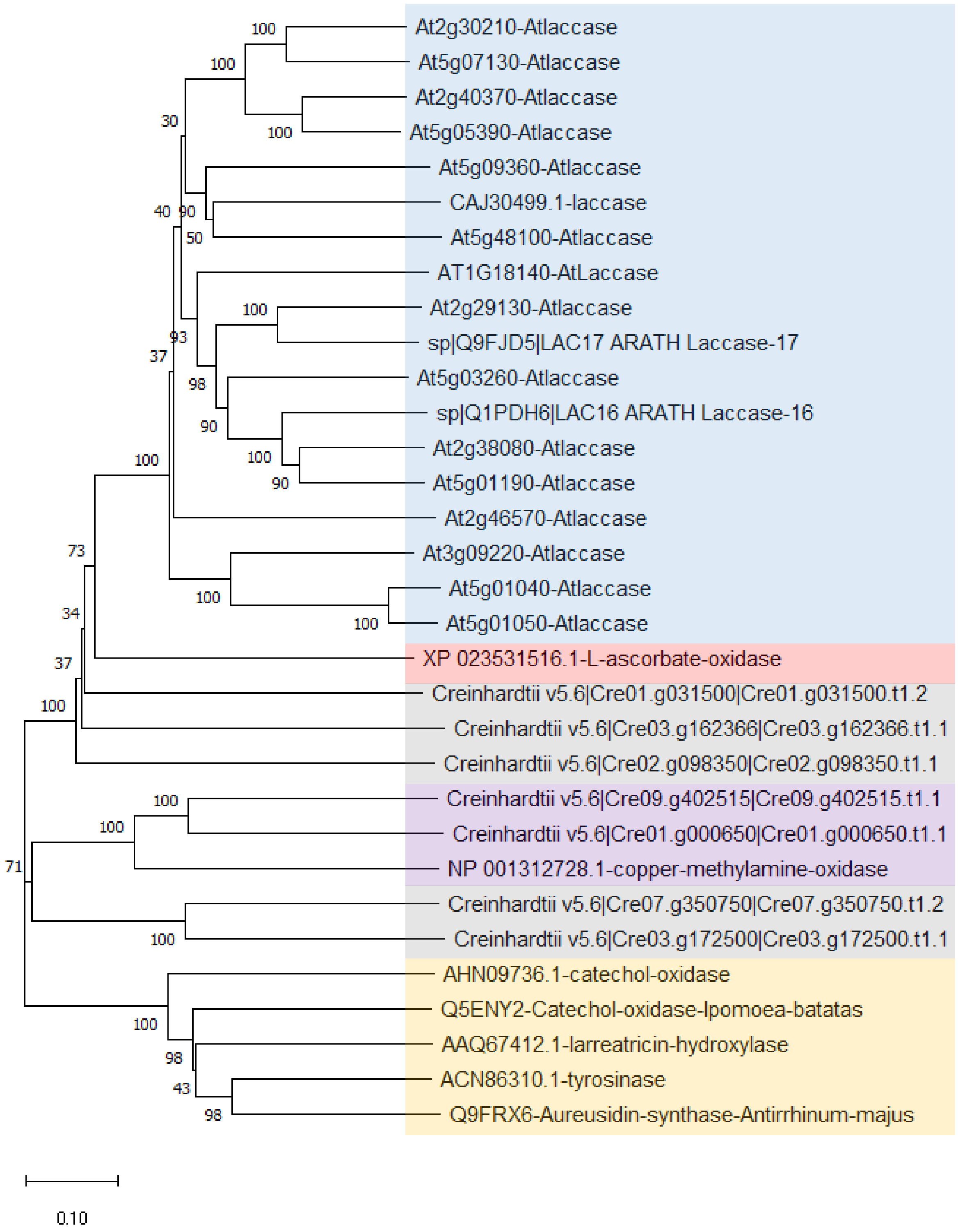

Figure S1 | Phylogenetic analyses of genome-derived copper enzyme genes with characterized copper enzyme genes. *Chlamydomonas reinhardtii (v5.6).* Laccases highlighted in blue, ascorbate oxidases highlighted in red, undefined copper enzymes highlighted in grey, copper-methylamine-oxidases highlighted in purple, T3 polyphenol oxidases highlighted in orange.

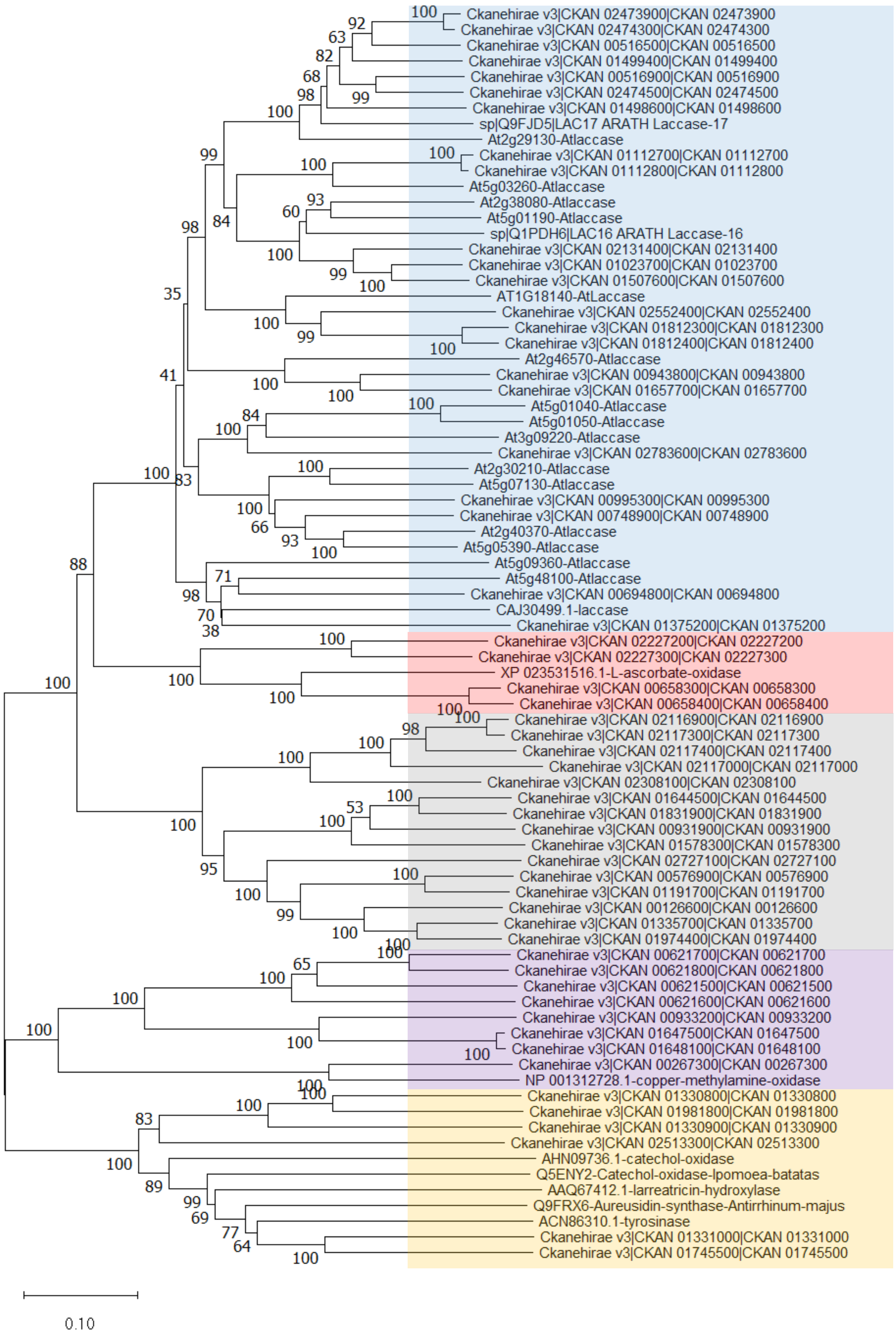

Figure S1 | Phylogenetic analyses of genome-derived copper enzyme genes with characterized copper enzyme genes. *Cinnamomum kanehirae (v3).* Laccases highlighted in blue, ascorbate oxidases highlighted in red, undefined copper enzymes highlighted in grey, copper-methylamine-oxidases highlighted in purple, T3 polyphenol oxidases highlighted in orange.

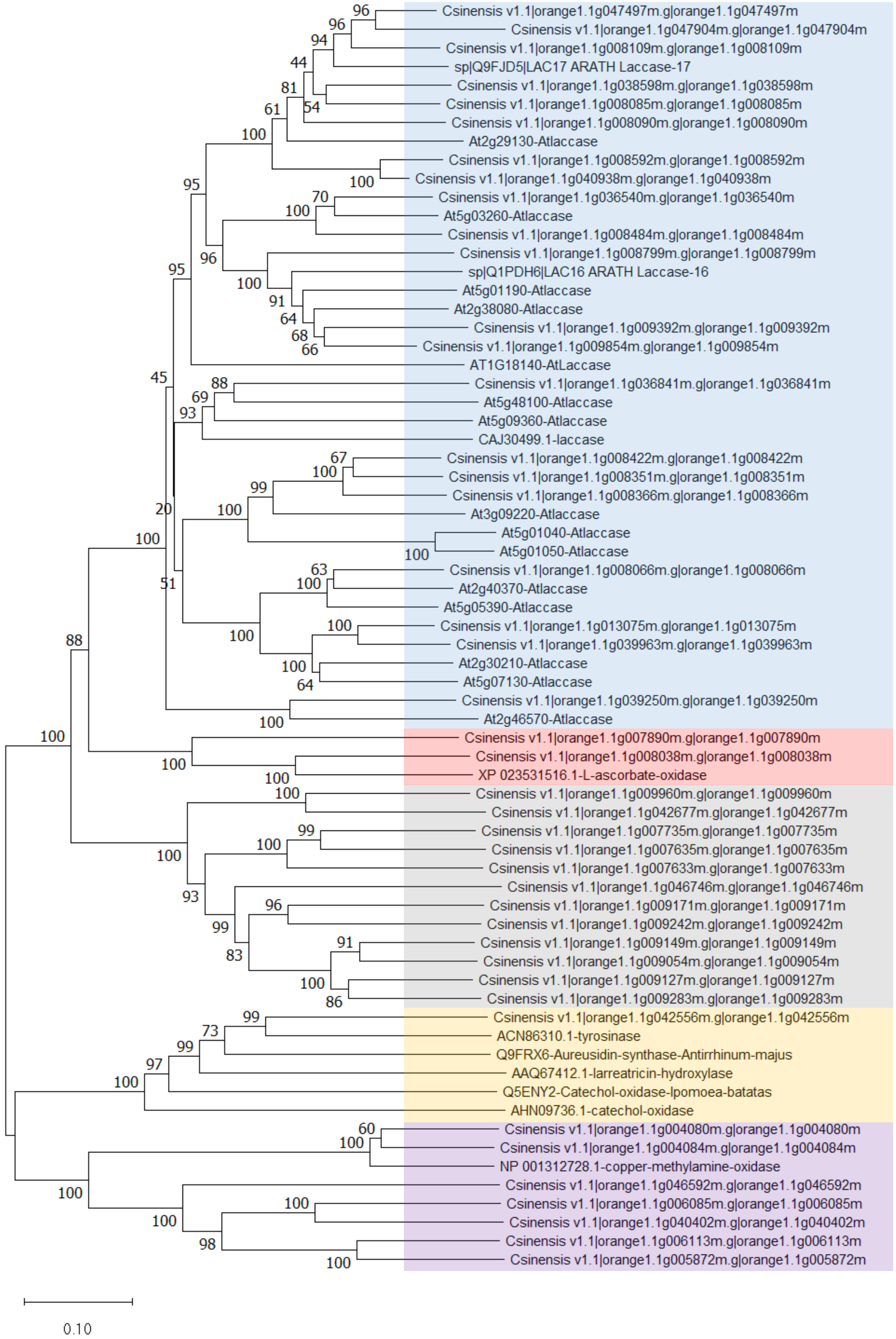

Figure S1 | Phylogenetic analyses of genome-derived copper enzyme genes with characterized copper enzyme genes. *Citrus sinensis (v1.1).* Laccases highlighted in blue, ascorbate oxidases highlighted in red, undefined copper enzymes highlighted in grey, copper-methylamine-oxidases highlighted in purple, T3 polyphenol oxidases highlighted in orange.

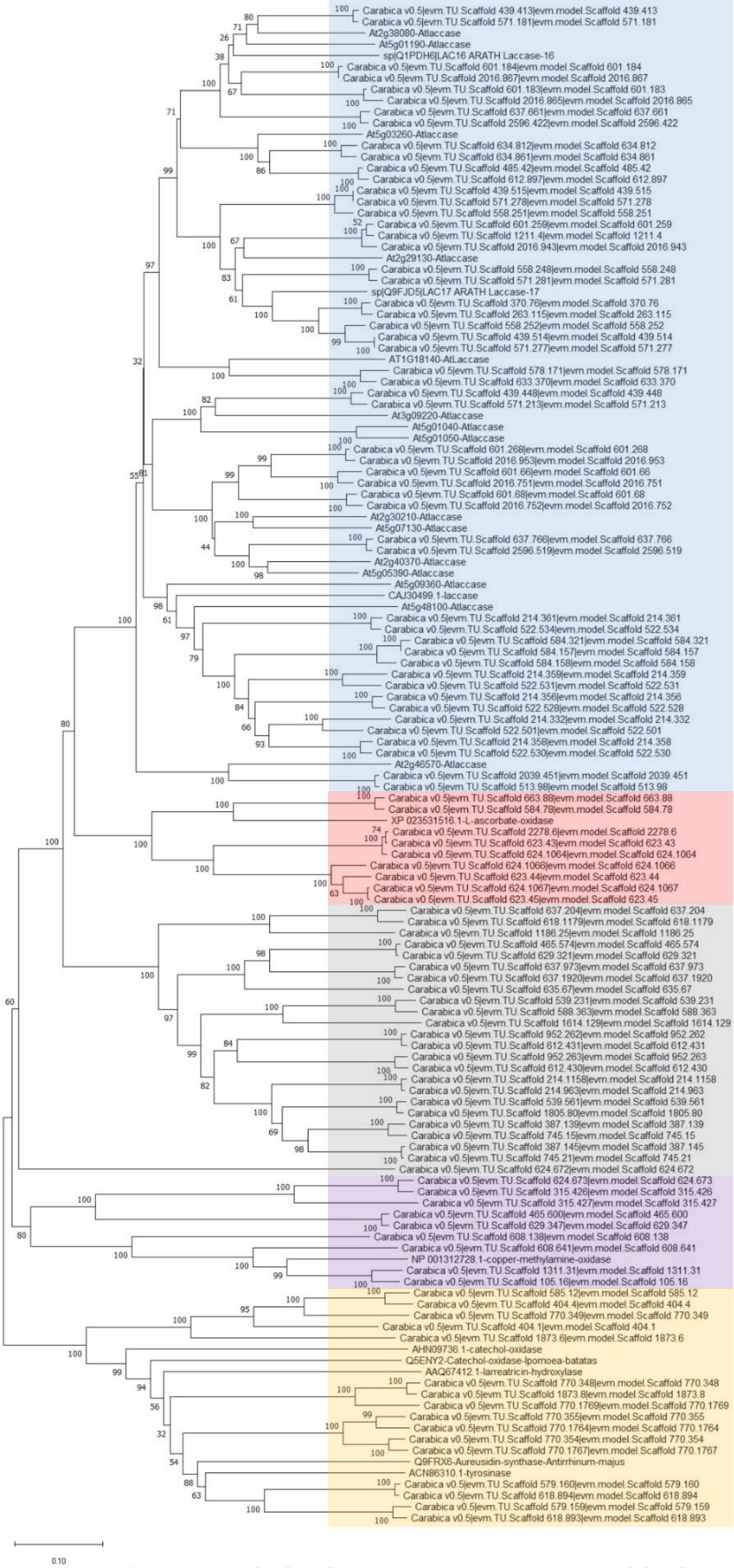

Figure S1 | Phylogenetic analyses of genome-derived copper enzyme genes with characterized copper enzyme genes. *Coffea arabica (v0.5).* Laccases highlighted in blue, ascorbate oxidases highlighted in red, undefined copper enzymes highlighted in grey, copper-methylamine-oxidases highlighted in purple, T3 polyphenol oxidases highlighted in orange.

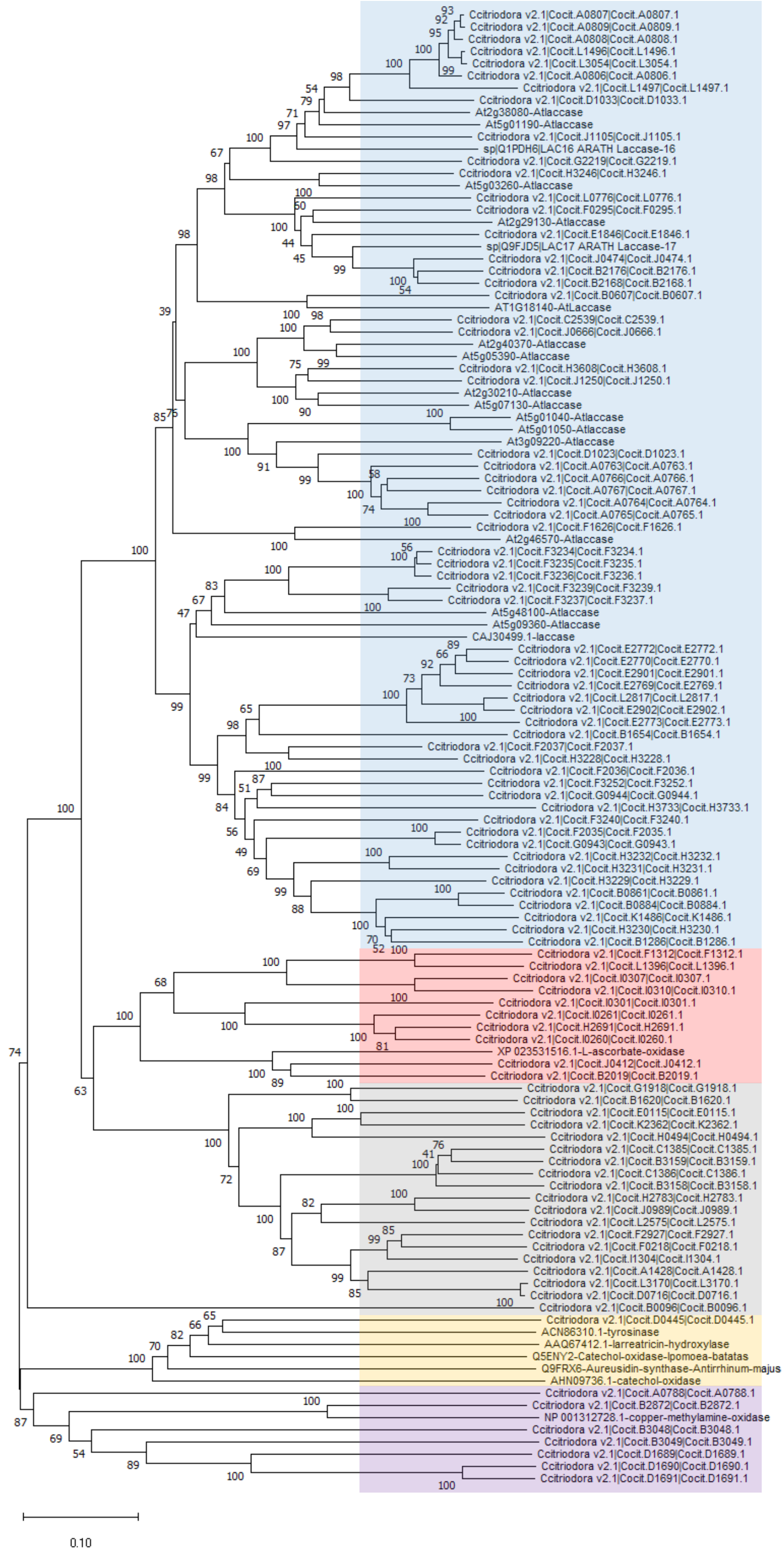

Figure S1 | Phylogenetic analyses of genome-derived copper enzyme genes with characterized copper enzyme genes. *Corymbia citriodora (v2.1).* Laccases highlighted in blue, ascorbate oxidases highlighted in red, undefined copper enzymes highlighted in grey, copper-methylamine-oxidases highlighted in purple, T3 polyphenol oxidases highlighted in orange.

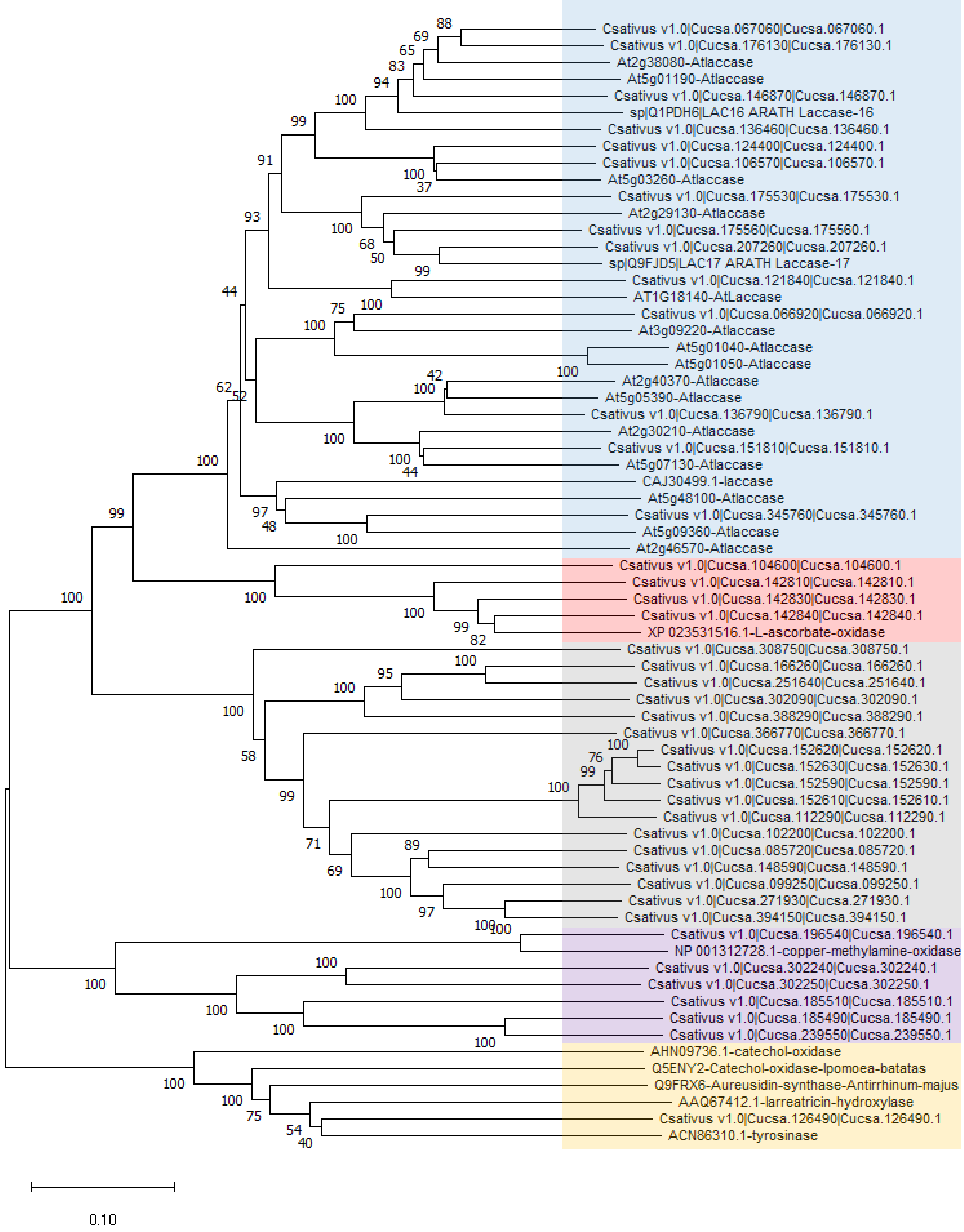

Figure S1 | Phylogenetic analyses of genome-derived copper enzyme genes with characterized copper enzyme genes. *Cucumis sativus (v1.0).* Laccases highlighted in blue, ascorbate oxidases highlighted in red, undefined copper enzymes highlighted in grey, copper-methylamine-oxidases highlighted in purple, T3 polyphenol oxidases highlighted in orange.

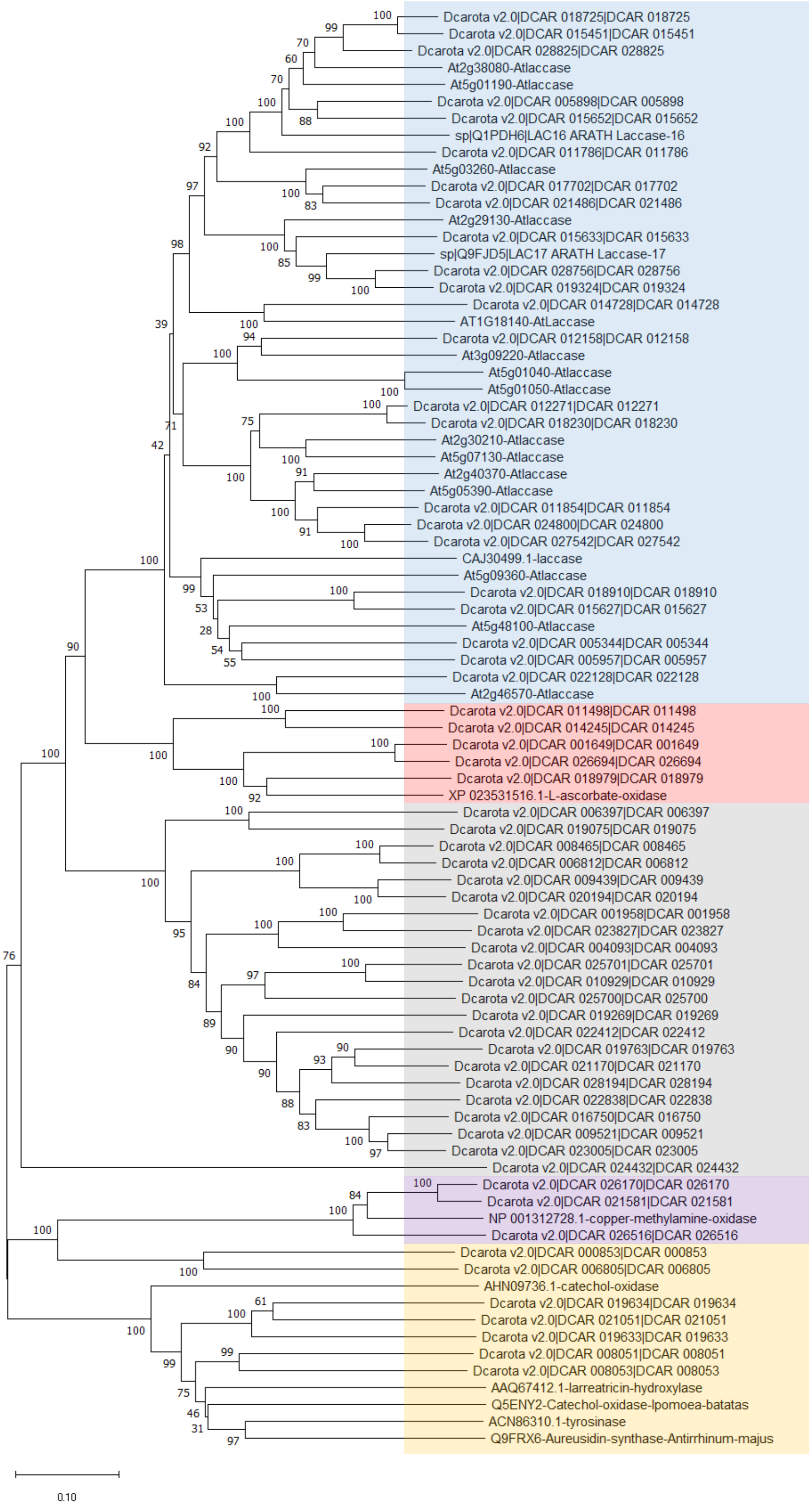

Figure S1 | Phylogenetic analyses of genome-derived copper enzyme genes with characterized copper enzyme genes. *Daucus carota (v2.0).* Laccases highlighted in blue, ascorbate oxidases highlighted in red, undefined copper enzymes highlighted in grey, copper-methylamine-oxidases highlighted in purple, T3 polyphenol oxidases highlighted in orange.

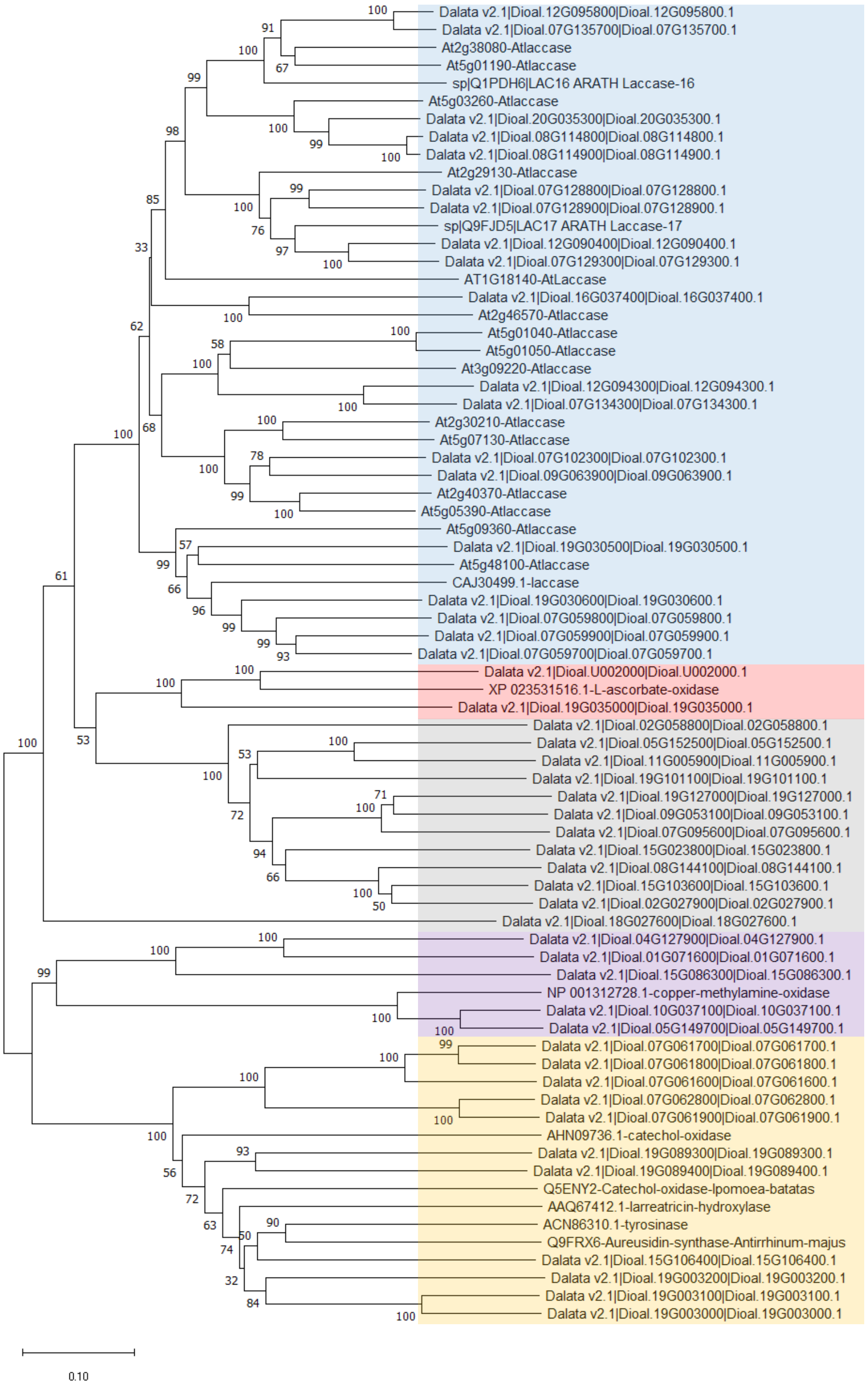

Figure S1 | Phylogenetic analyses of genome-derived copper enzyme genes with characterized copper enzyme genes. *Dioscorea alata (v2.1).* Laccases highlighted in blue, ascorbate oxidases highlighted in red, undefined copper enzymes highlighted in grey, copper-methylamine-oxidases highlighted in purple, T3 polyphenol oxidases highlighted in orange.

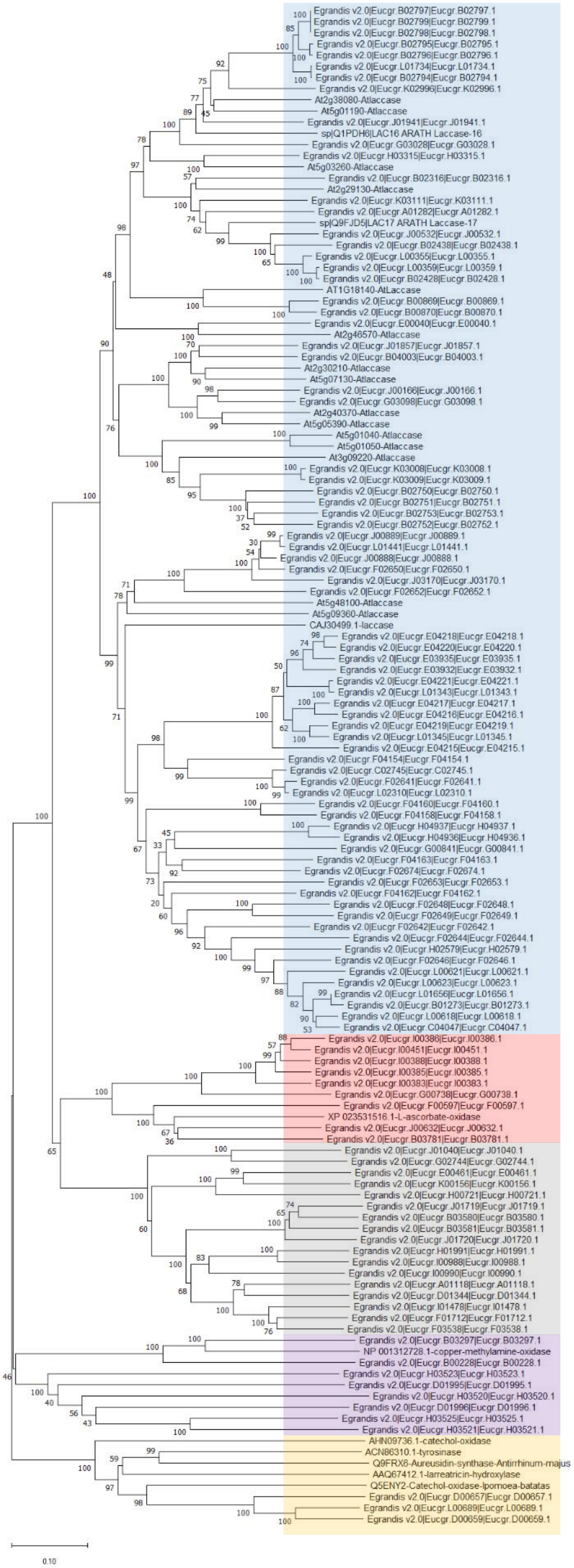

Figure S1 | Phylogenetic analyses of genome-derived copper enzyme genes with characterized copper enzyme genes. *Eucalyptus grandis (v2.0).* Laccases highlighted in blue, ascorbate oxidases highlighted in red, undefined copper enzymes highlighted in grey, copper-methylamine-oxidases highlighted in purple, T3 polyphenol oxidases highlighted in orange.

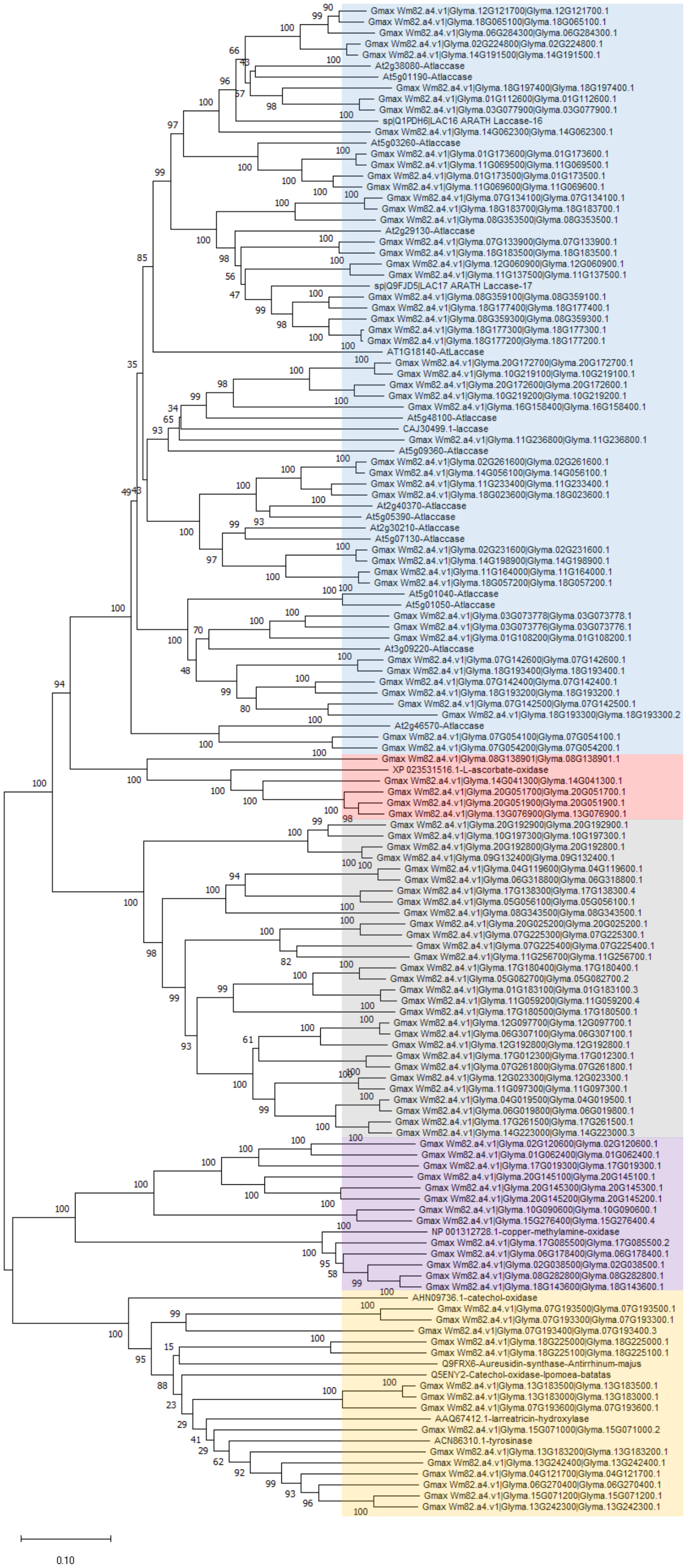

Figure S1 | Phylogenetic analyses of genome-derived copper enzyme genes with characterized copper enzyme genes. *Glycine max (Wm82.a4.v1).* Laccases highlighted in blue, ascorbate oxidases highlighted in red, undefined copper enzymes highlighted in grey, copper-methylamine-oxidases highlighted in purple, T3 polyphenol oxidases highlighted in orange.

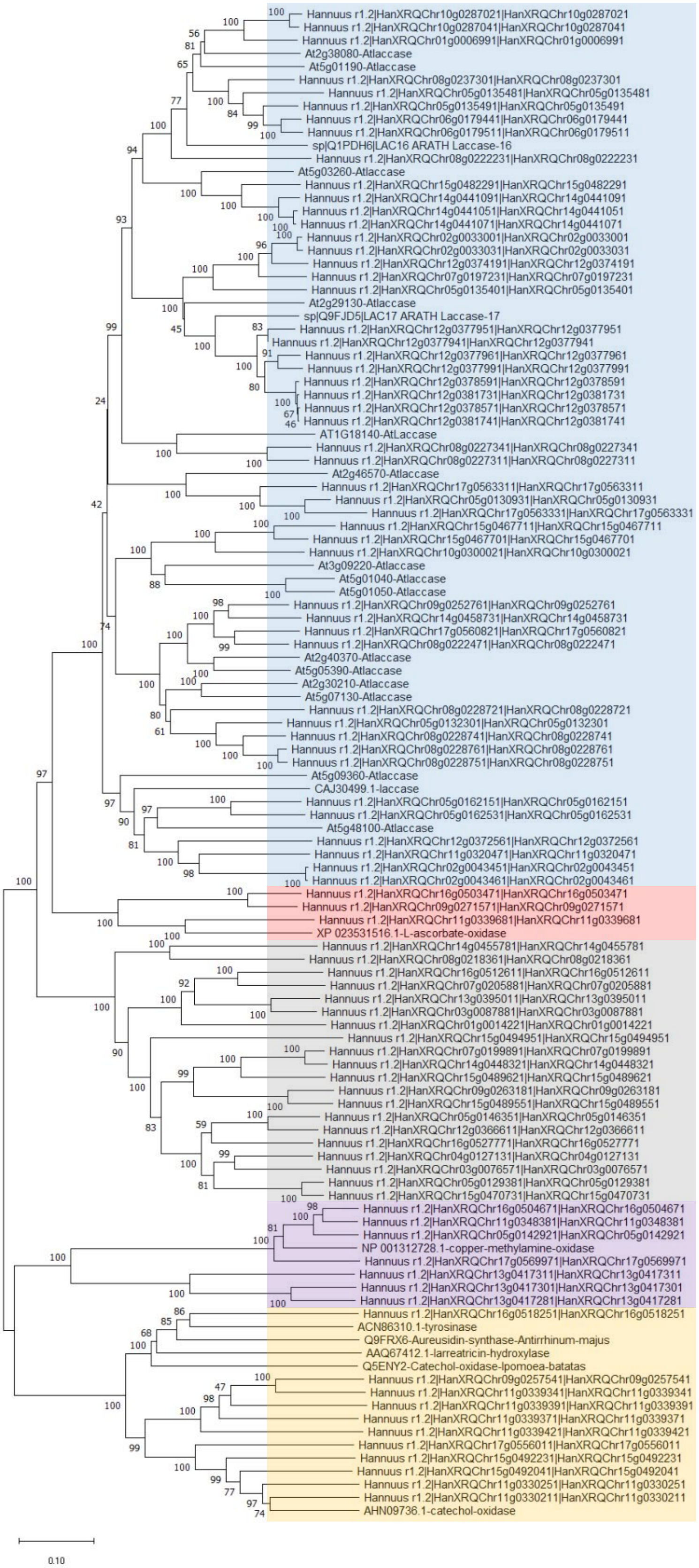

Figure S1 | Phylogenetic analyses of genome-derived copper enzyme genes with characterized copper enzyme genes. *Helianthus annuus (r1.2).* Laccases highlighted in blue, ascorbate oxidases highlighted in red, undefined copper enzymes highlighted in grey, copper-methylamine-oxidases highlighted in purple, T3 polyphenol oxidases highlighted in orange.

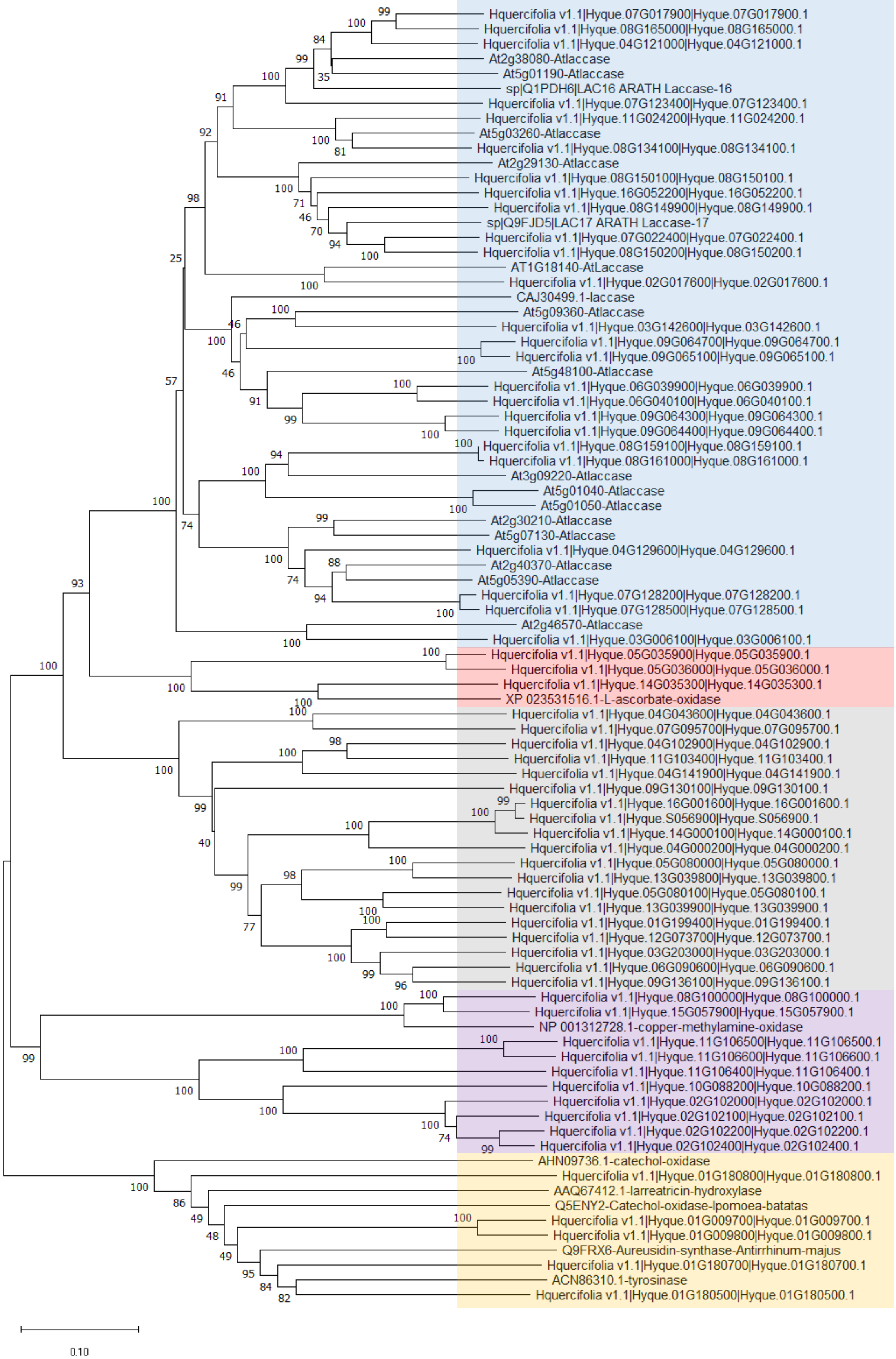

Figure S1 | Phylogenetic analyses of genome-derived copper enzyme genes with characterized copper enzyme genes. *Helianthus annuus (r1.2).* Laccases highlighted in blue, ascorbate oxidases highlighted in red, undefined copper enzymes highlighted in grey, copper-methylamine-oxidases highlighted in purple, T3 polyphenol oxidases highlighted in orange.

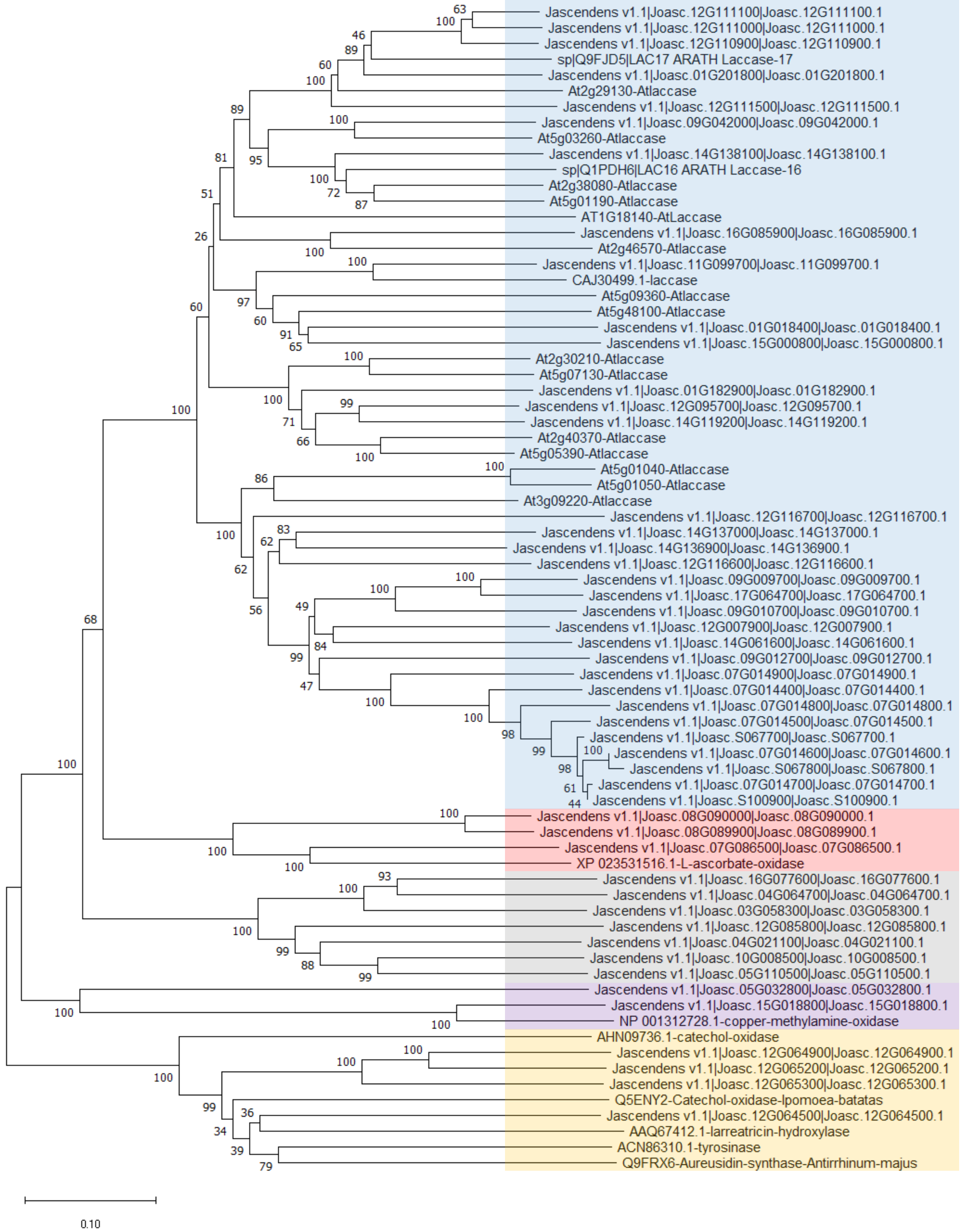

Figure S1 | Phylogenetic analyses of genome-derived copper enzyme genes with characterized copper enzyme genes. *Joinvillea ascendens (v1.1).* Laccases highlighted in blue, ascorbate oxidases highlighted in red, undefined copper enzymes highlighted in grey, copper-methylamine-oxidases highlighted in purple, T3 polyphenol oxidases highlighted in orange.

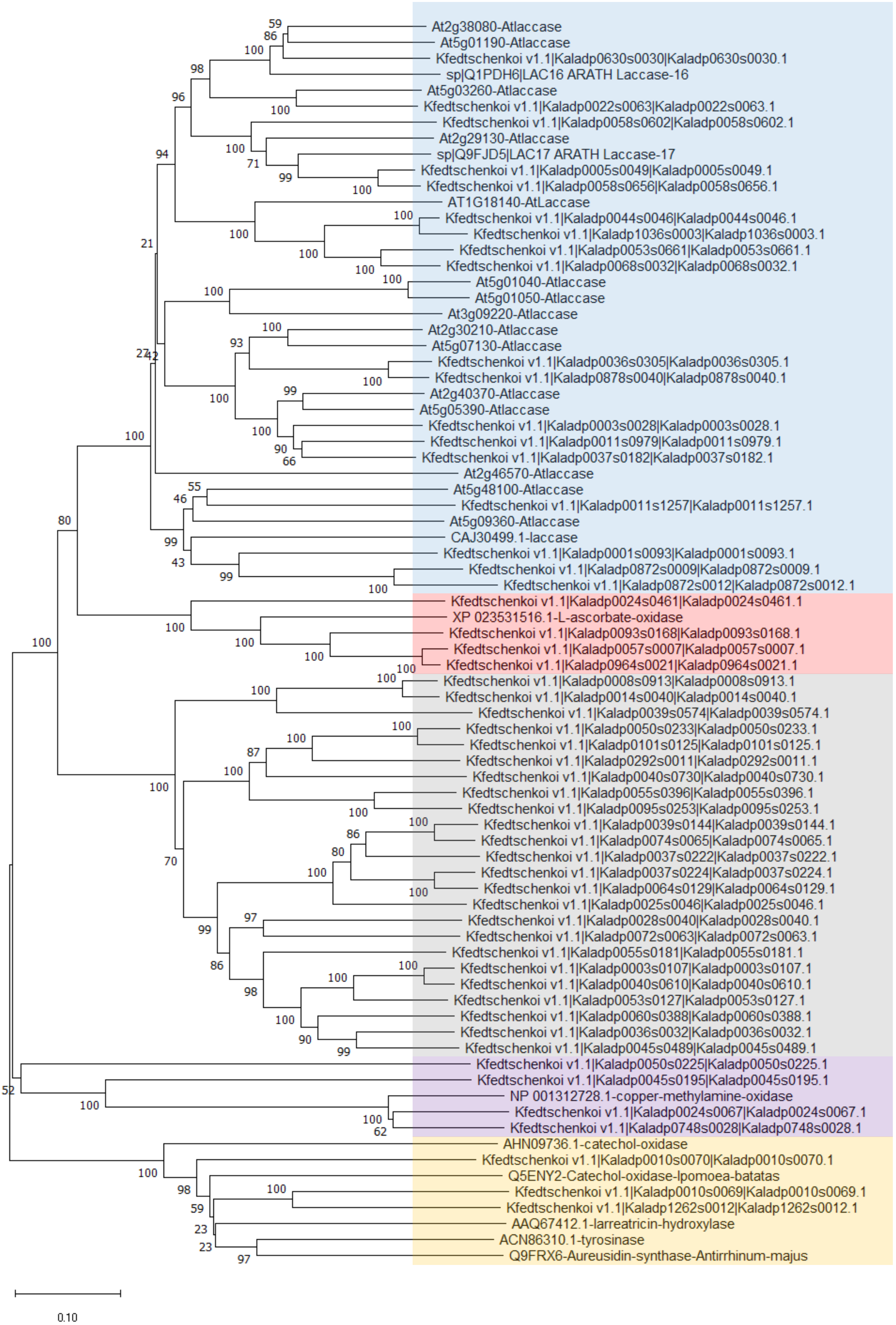

Figure S1 | Phylogenetic analyses of genome-derived copper enzyme genes with characterized copper enzyme genes. *Kalanchoe fedtschenkoi (v1.1).* Laccases highlighted in blue, ascorbate oxidases highlighted in red, undefined copper enzymes highlighted in grey, copper-methylamine-oxidases highlighted in purple, T3 polyphenol oxidases highlighted in orange.

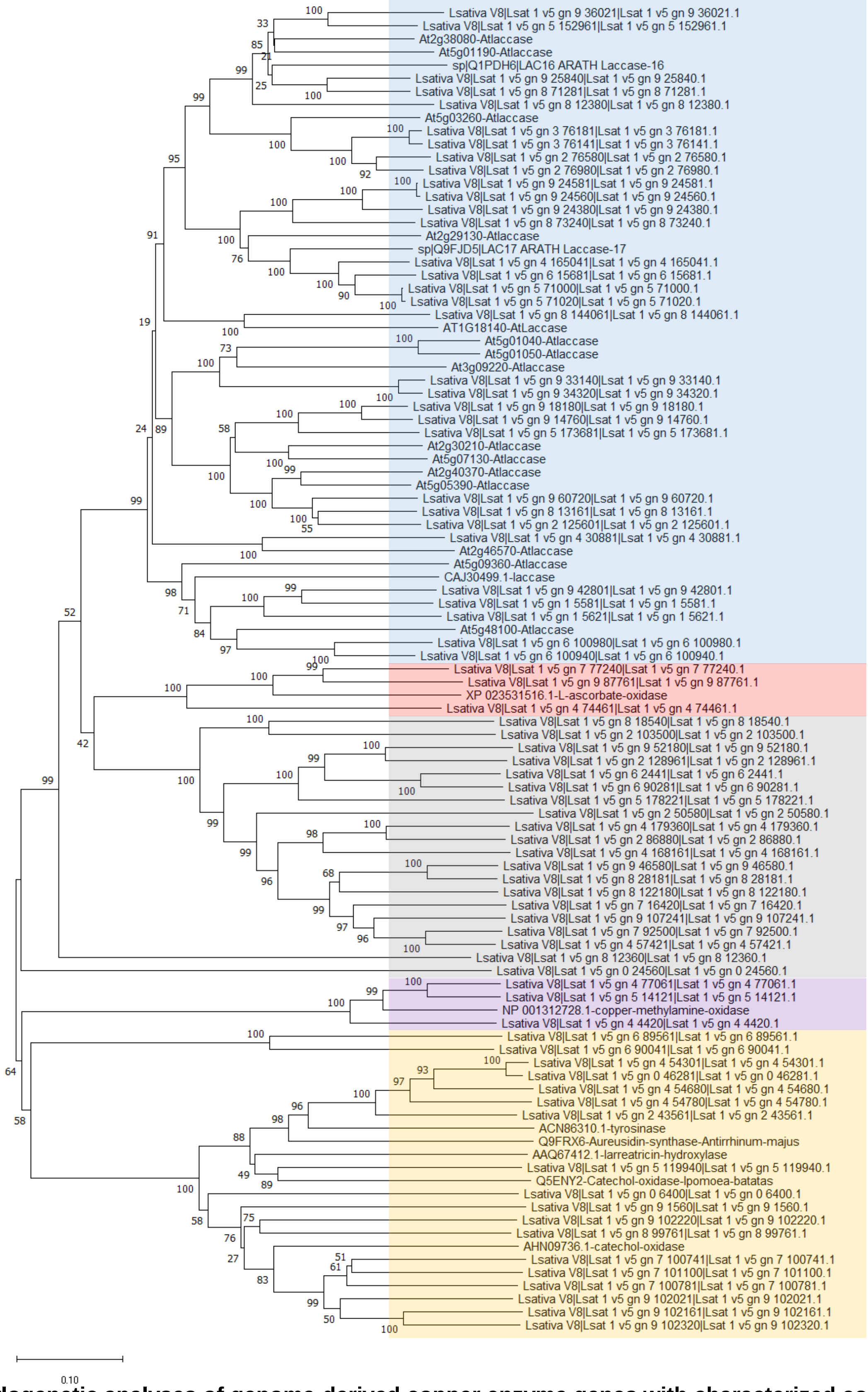

Figure S1 | Phylogenetic analyses of genome-derived copper enzyme genes with characterized copper enzyme genes. *Lactuca sativa (V8).* Laccases highlighted in blue, ascorbate oxidases highlighted in red, undefined copper enzymes highlighted in grey, copper-methylamine-oxidases highlighted in purple, T3 polyphenol oxidases highlighted in orange.

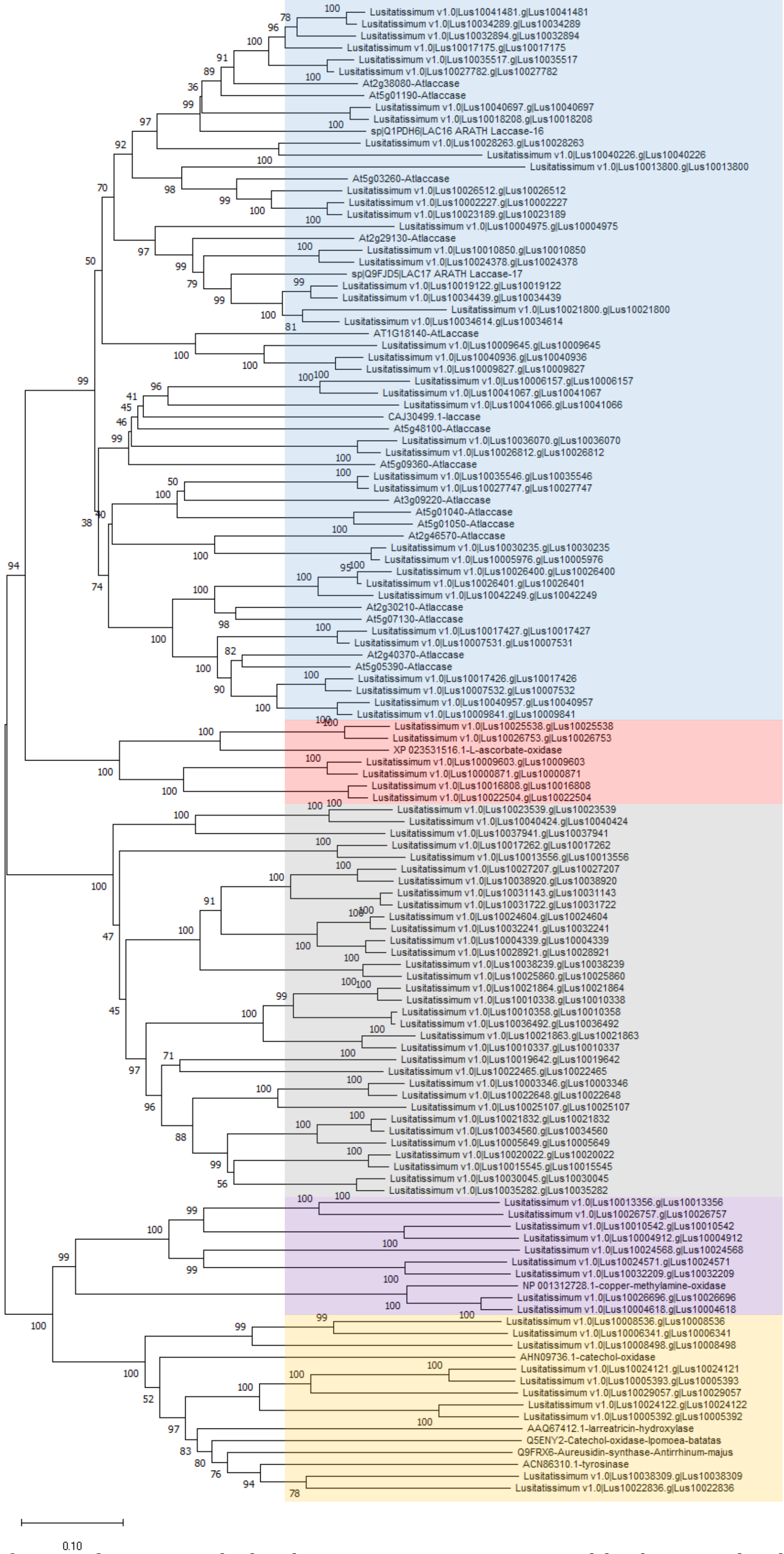

Figure S1 | Phylogenetic analyses of genome-derived copper enzyme genes with characterized copper enzyme genes. *Linum usitatissimum (v1.0).* Laccases highlighted in blue, ascorbate oxidases highlighted in red, undefined copper enzymes highlighted in grey, copper-methylamine-oxidases highlighted in purple, T3 polyphenol oxidases highlighted in orange.

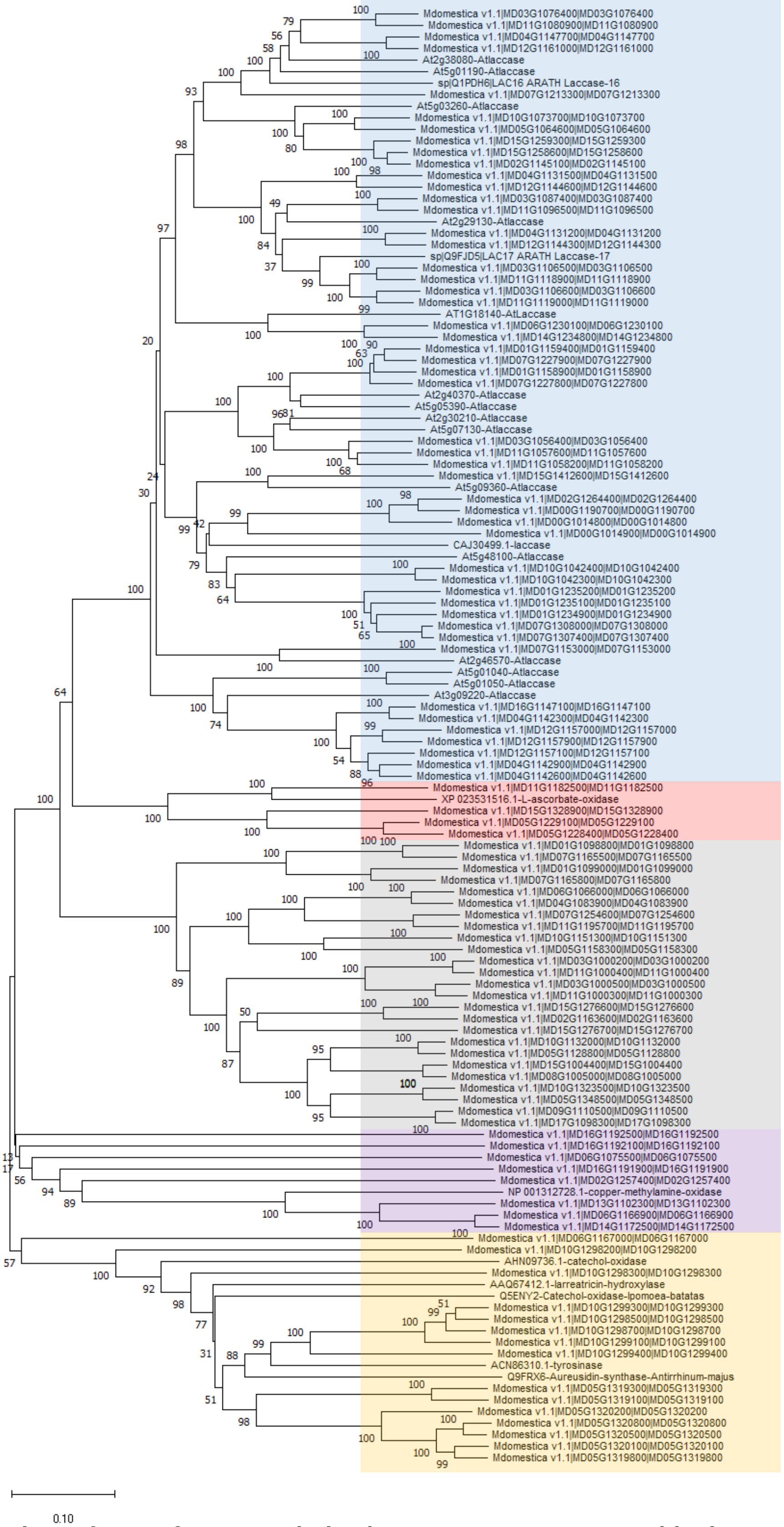

Figure S1 | Phylogenetic analyses of genome-derived copper enzyme genes with characterized copper enzyme genes. *Malus domestica (v1.1).* Laccases highlighted in blue, ascorbate oxidases highlighted in red, undefined copper enzymes highlighted in grey, copper-methylamine-oxidases highlighted in purple, T3 polyphenol oxidases highlighted in orange.

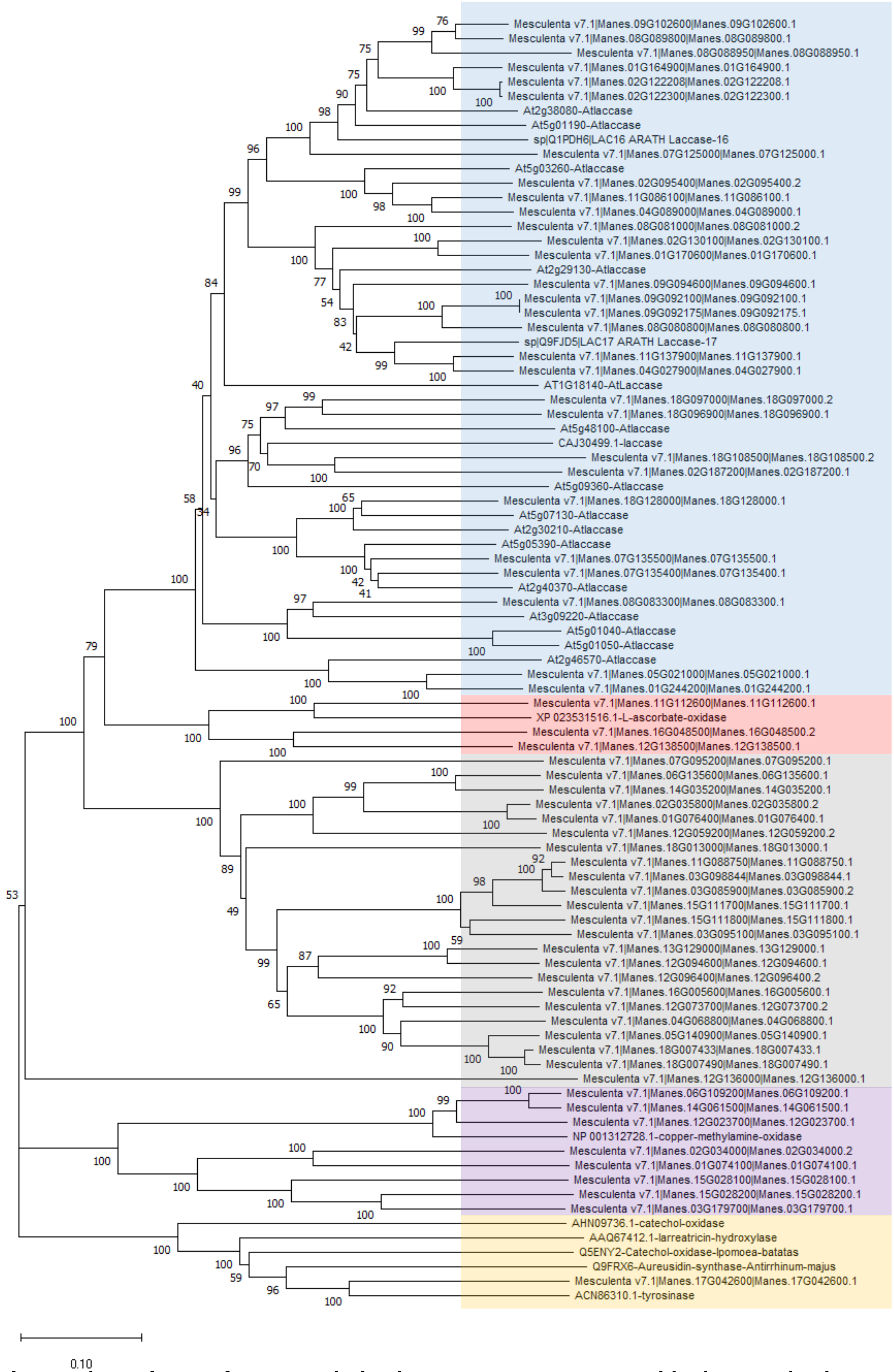

Figure S1 | Phylogenetic analyses of genome-derived copper enzyme genes with characterized copper enzyme genes. *Manihot esculenta (v7.1).* Laccases highlighted in blue, ascorbate oxidases highlighted in red, undefined copper enzymes highlighted in grey, copper-methylamine-oxidases highlighted in purple, T3 polyphenol oxidases highlighted in orange.

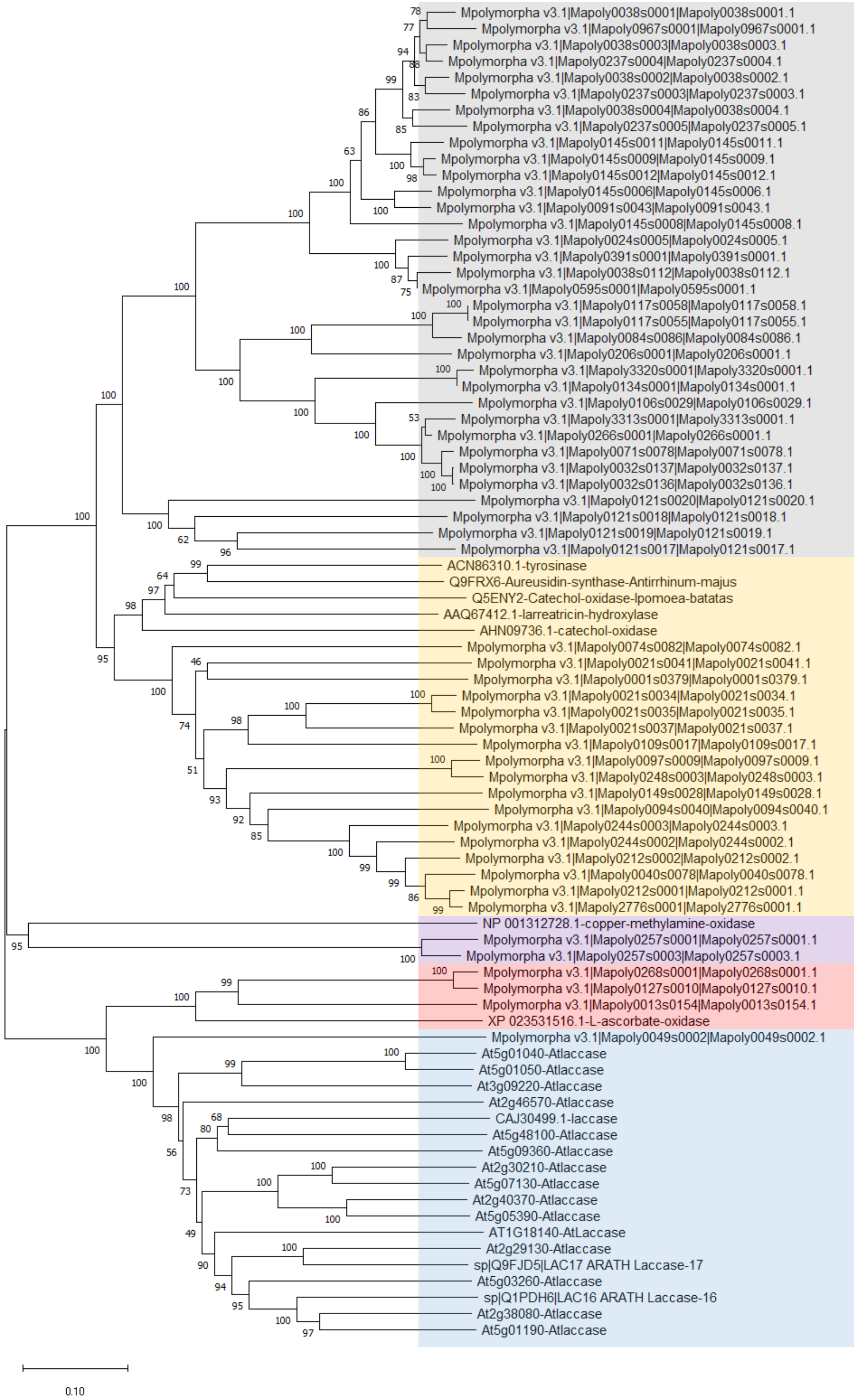

Figure S1 | Phylogenetic analyses of genome-derived copper enzyme genes with characterized copper enzyme genes. *Marchantia polymorpha (v3.1).* Laccases highlighted in blue, ascorbate oxidases highlighted in red, undefined copper enzymes highlighted in grey, copper-methylamine-oxidases highlighted in purple, T3 polyphenol oxidases highlighted in orange.

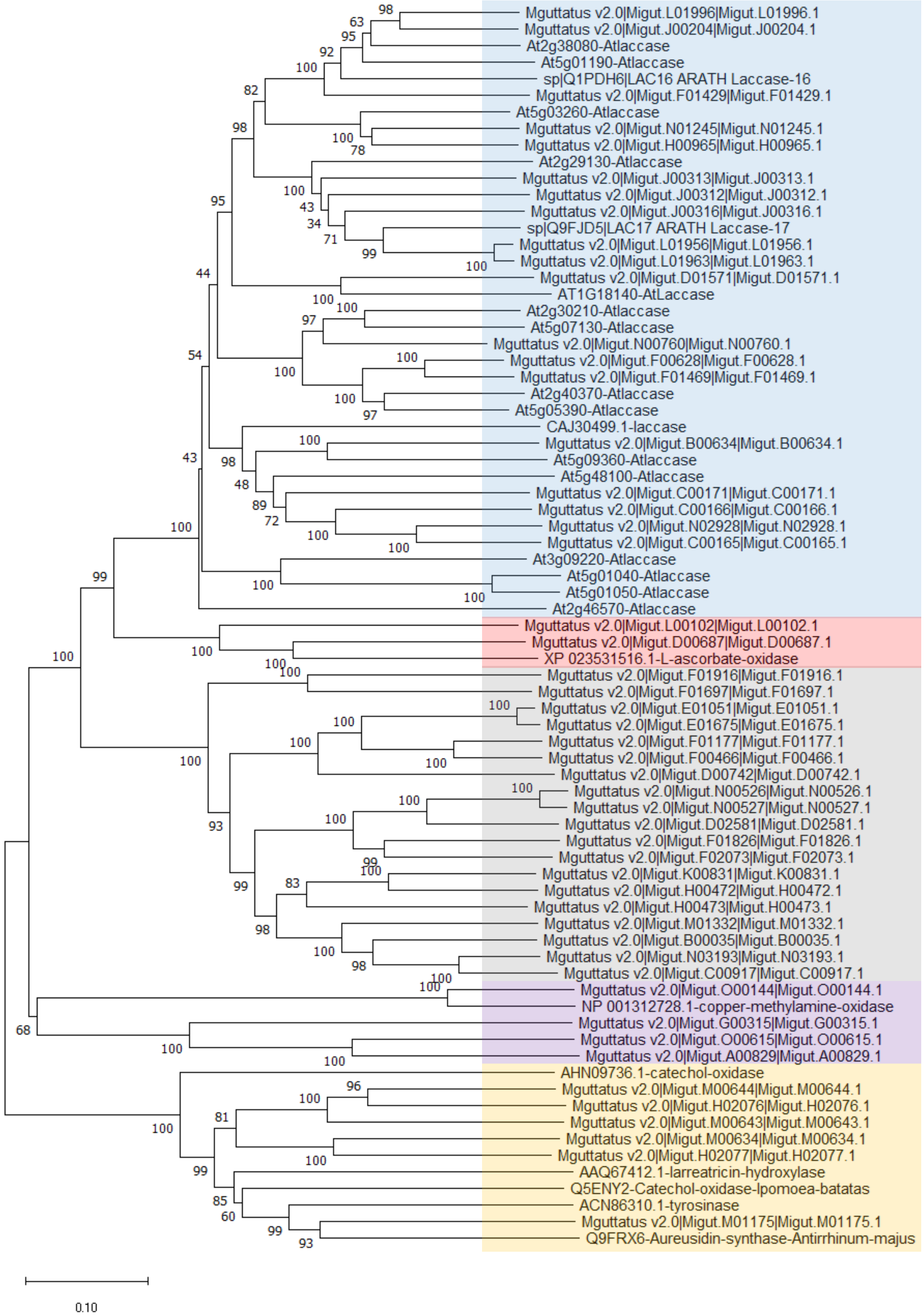

Figure S1 | Phylogenetic analyses of genome-derived copper enzyme genes with characterized copper enzyme genes. *Mimulus guttatus TOL (v5.0).* Laccases highlighted in blue, ascorbate oxidases highlighted in red, undefined copper enzymes highlighted in grey, copper-methylamine-oxidases highlighted in purple, T3 polyphenol oxidases highlighted in orange.

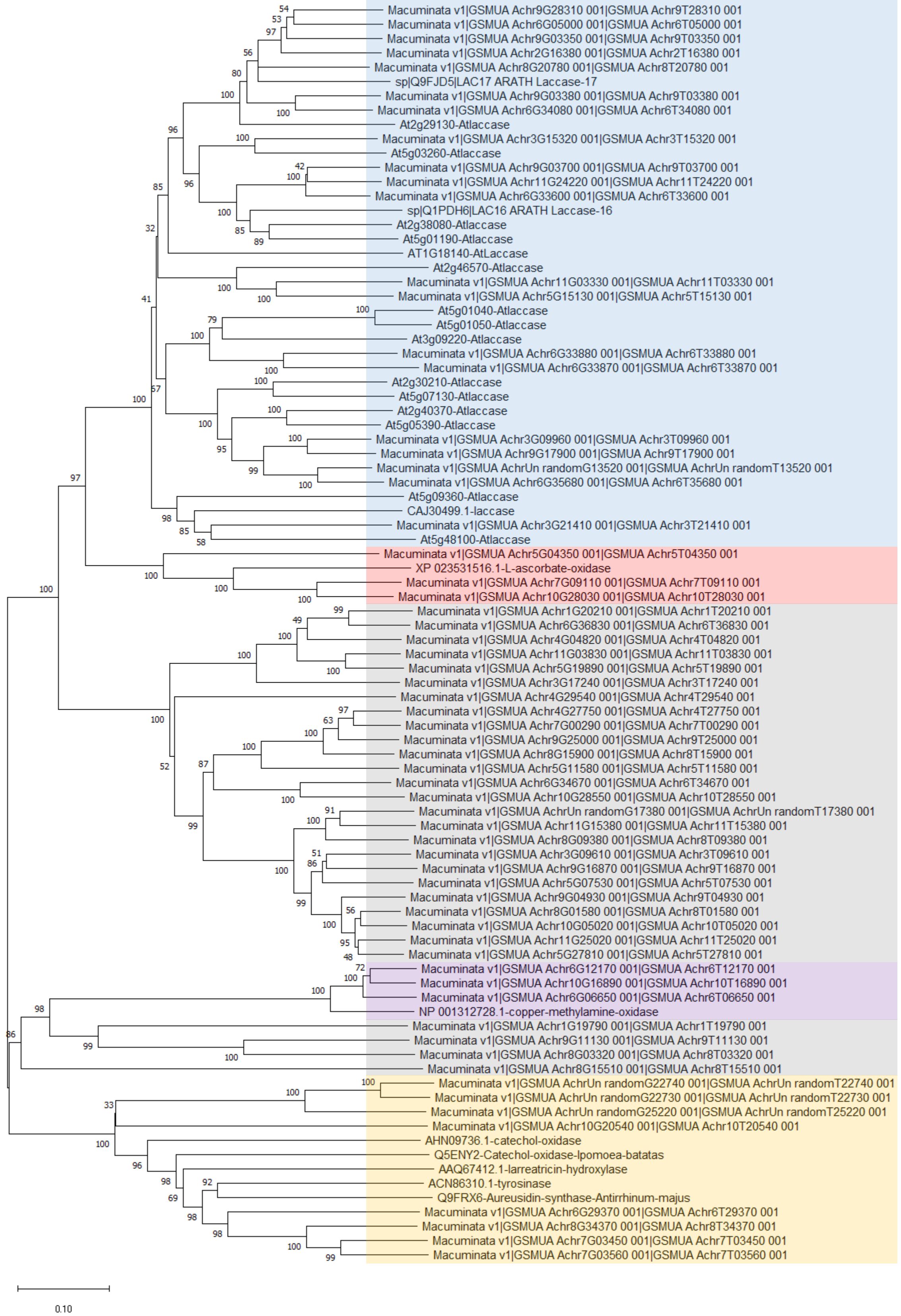

Figure S1 | Phylogenetic analyses of genome-derived copper enzyme genes with characterized copper enzyme genes. *Musa acuminata (v1).* Laccases highlighted in blue, ascorbate oxidases highlighted in red, undefined copper enzymes highlighted in grey, copper-methylamine-oxidases highlighted in purple, T3 polyphenol oxidases highlighted in orange.

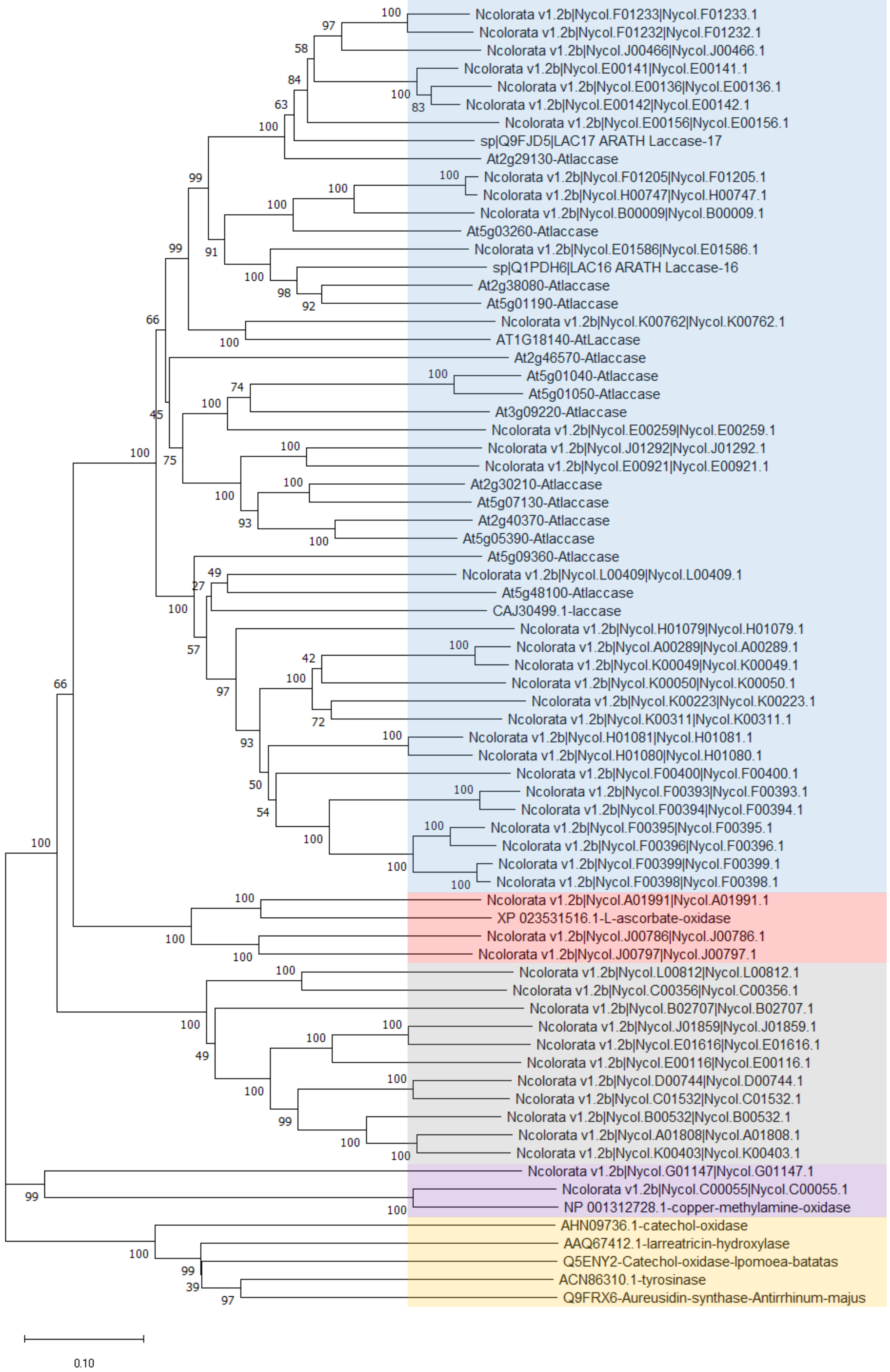

Figure S1 | Phylogenetic analyses of genome-derived copper enzyme genes with characterized copper enzyme genes. *Nymphaea colorata (v1.2).* Laccases highlighted in blue, ascorbate oxidases highlighted in red, undefined copper enzymes highlighted in grey, copper-methylamine-oxidases highlighted in purple, T3 polyphenol oxidases highlighted in orange.

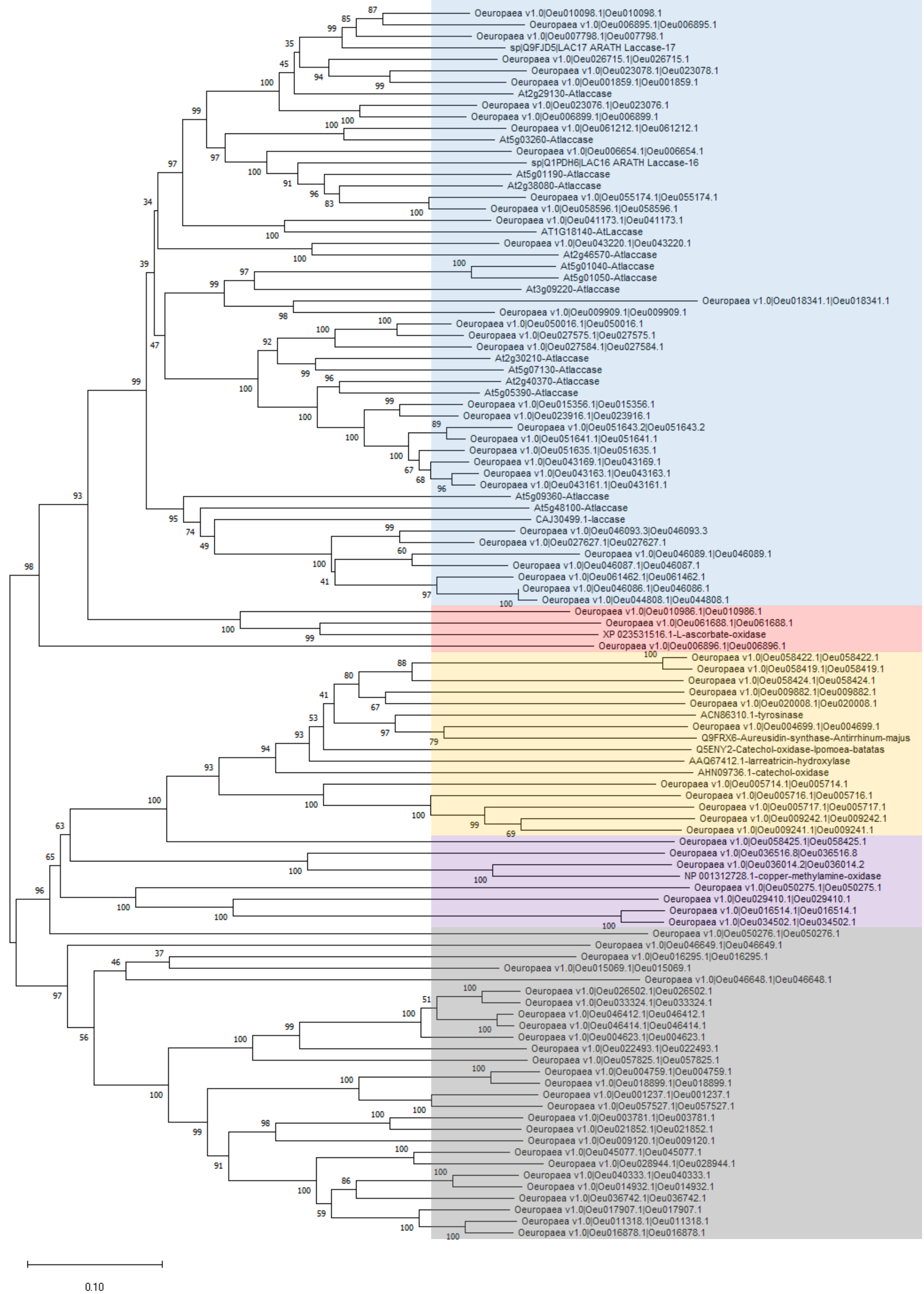

Figure S1 | Phylogenetic analyses of genome-derived copper enzyme genes with characterized copper enzyme genes. *Olea europaea (v1.0).* Laccases highlighted in blue, ascorbate oxidases highlighted in red, undefined copper enzymes highlighted in grey, copper-methylamine-oxidases highlighted in purple, T3 polyphenol oxidases highlighted in orange.

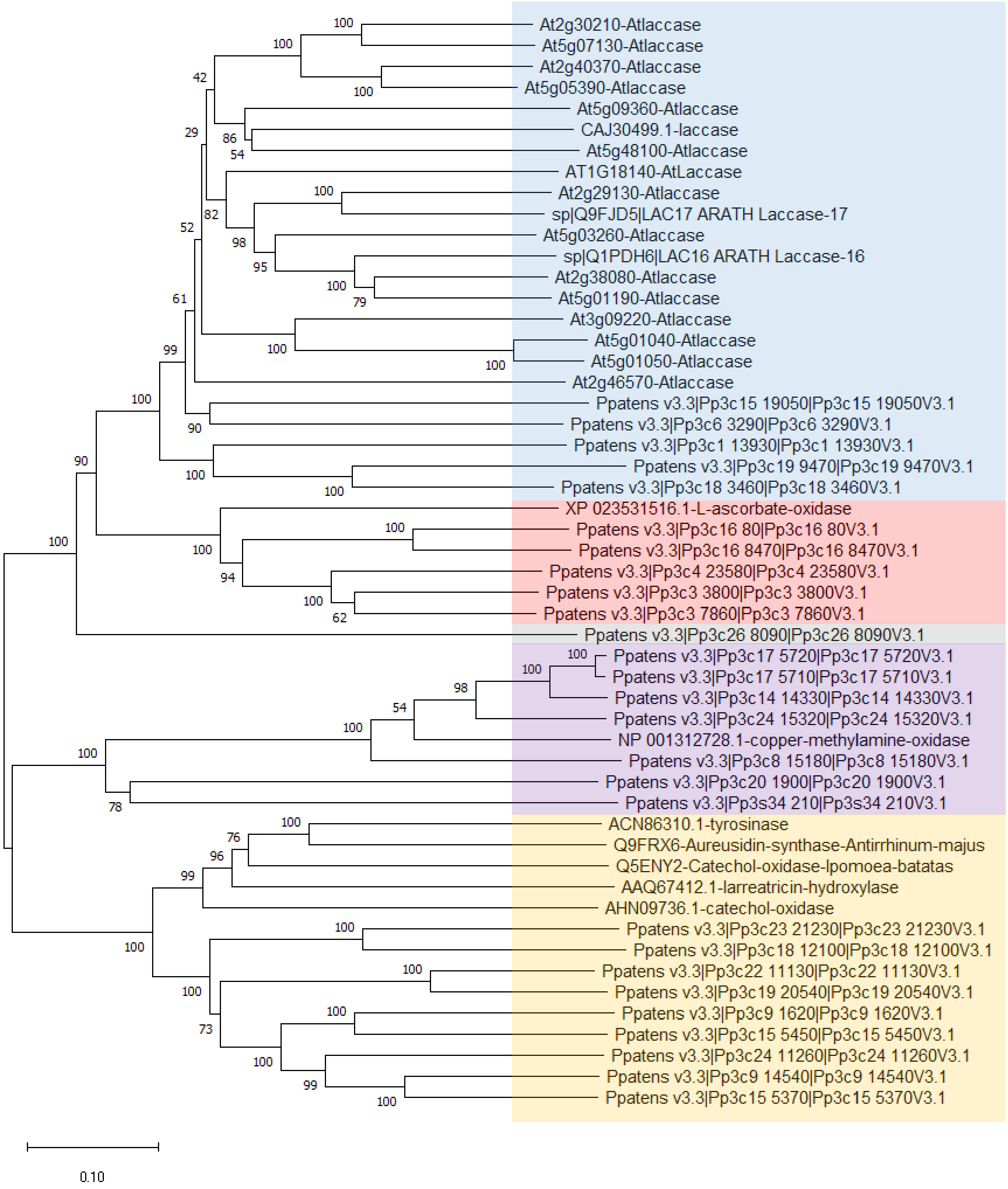

Figure S1 | Phylogenetic analyses of genome-derived copper enzyme genes with characterized copper enzyme genes. *Physcomitrium patens (v3.3).* Laccases highlighted in blue, ascorbate oxidases highlighted in red, undefined copper enzymes highlighted in grey, copper-methylamine-oxidases highlighted in purple, T3 polyphenol oxidases highlighted in orange.

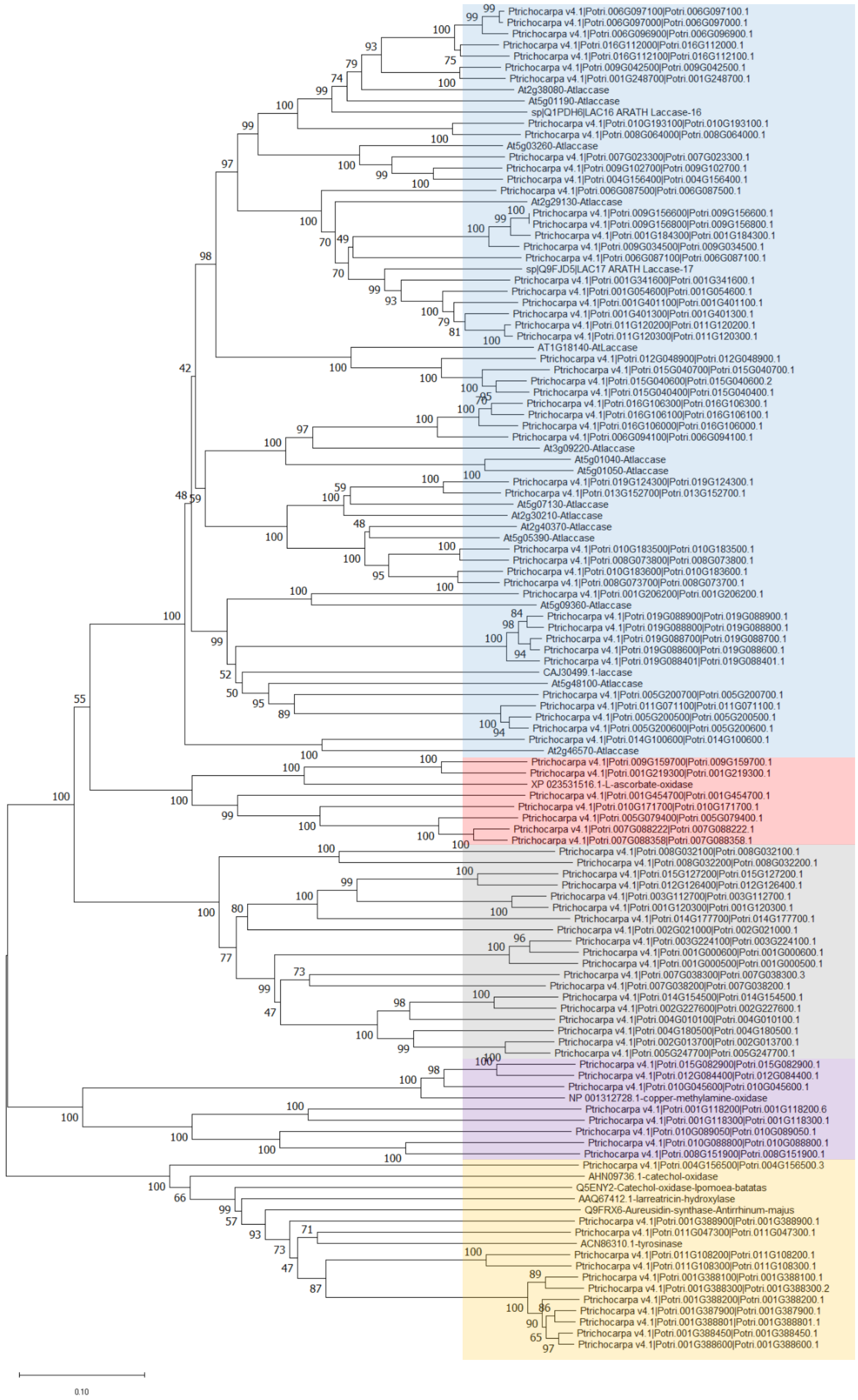

Figure S1 | Phylogenetic analyses of genome-derived copper enzyme genes with characterized copper enzyme genes. *Populus trichocarpa (v4.1).* Laccases highlighted in blue, ascorbate oxidases highlighted in red, undefined copper enzymes highlighted in grey, copper-methylamine-oxidases highlighted in purple, T3 polyphenol oxidases highlighted in orange.

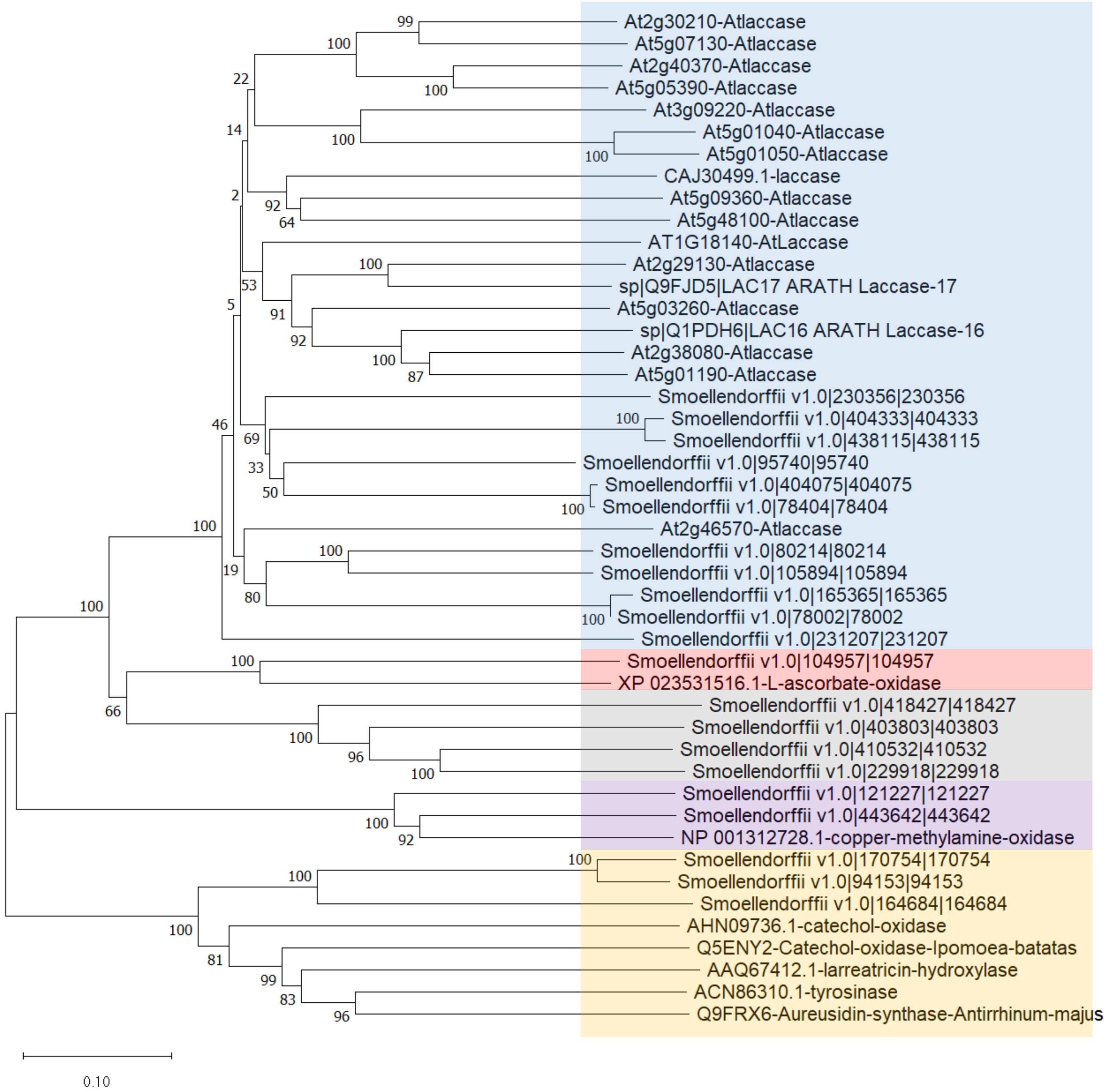

Figure S1 | Phylogenetic analyses of genome-derived copper enzyme genes with characterized copper enzyme genes. *Selaginella moellendorffii (v1.0).* Laccases highlighted in blue, ascorbate oxidases highlighted in red, undefined copper enzymes highlighted in grey, copper-methylamine-oxidases highlighted in purple, T3 polyphenol oxidases highlighted in orange.

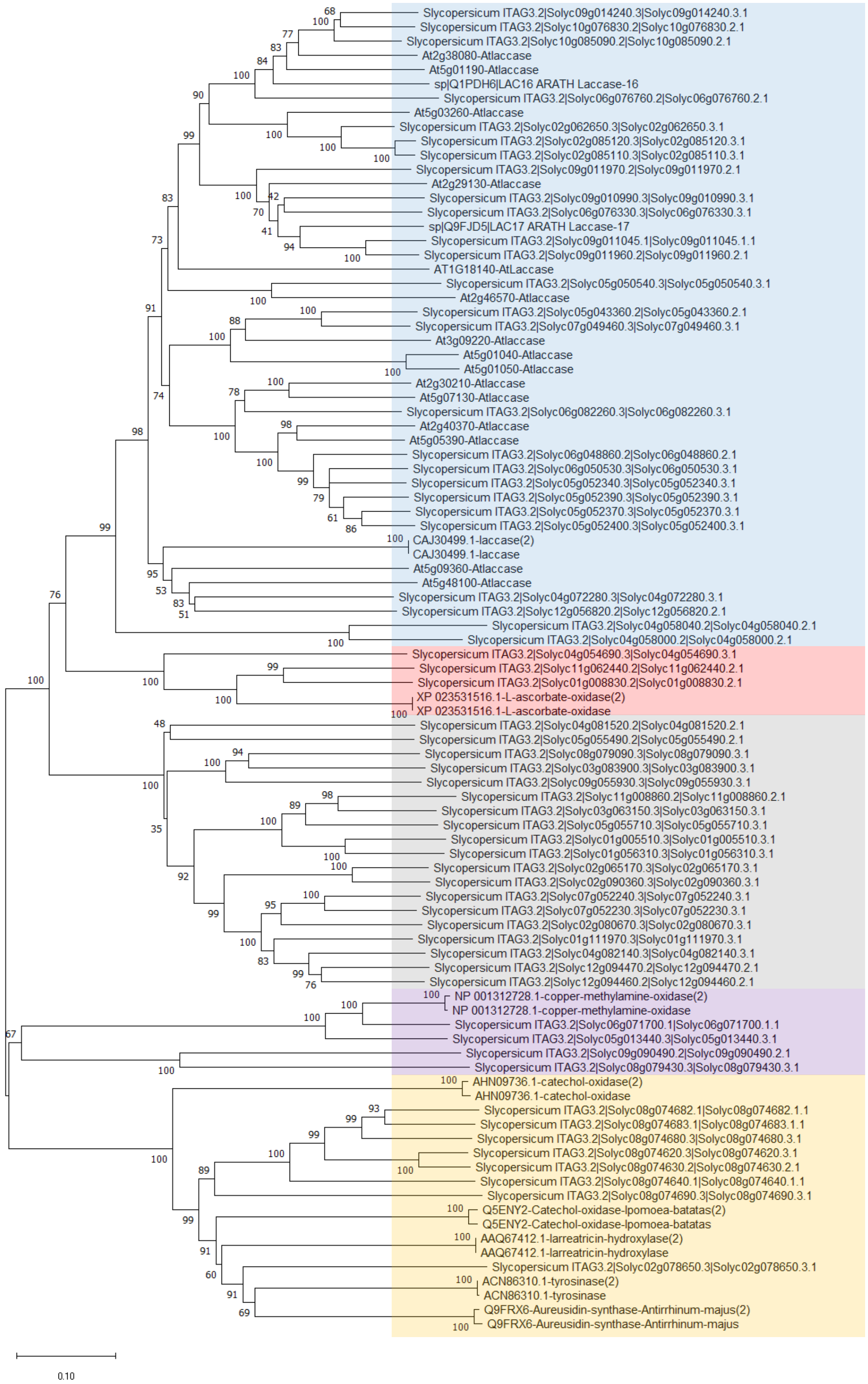

Figure S1 | Phylogenetic analyses of genome-derived copper enzyme genes with characterized copper enzyme genes. *Selaginella moellendorffii (v1.0).* Laccases highlighted in blue, ascorbate oxidases highlighted in red, undefined copper enzymes highlighted in grey, copper-methylamine-oxidases highlighted in purple, T3 polyphenol oxidases highlighted in orange.

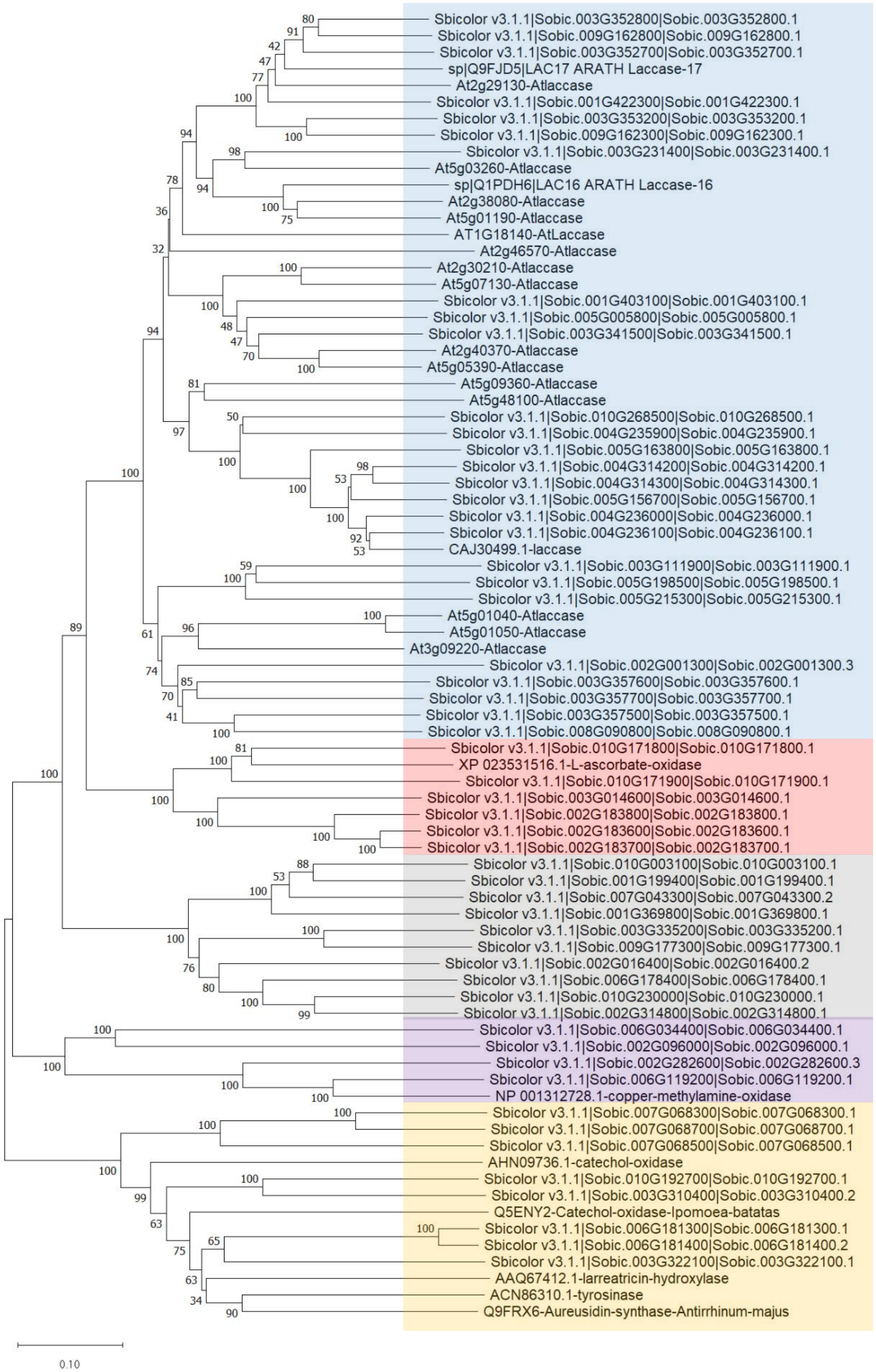

Figure S1 | Phylogenetic analyses of genome-derived copper enzyme genes with characterized copper enzyme genes. *Sorghum bicolor (v3.1.1).* Laccases highlighted in blue, ascorbate oxidases highlighted in red, undefined copper enzymes highlighted in grey, copper-methylamine-oxidases highlighted in purple, T3 polyphenol oxidases highlighted in orange.

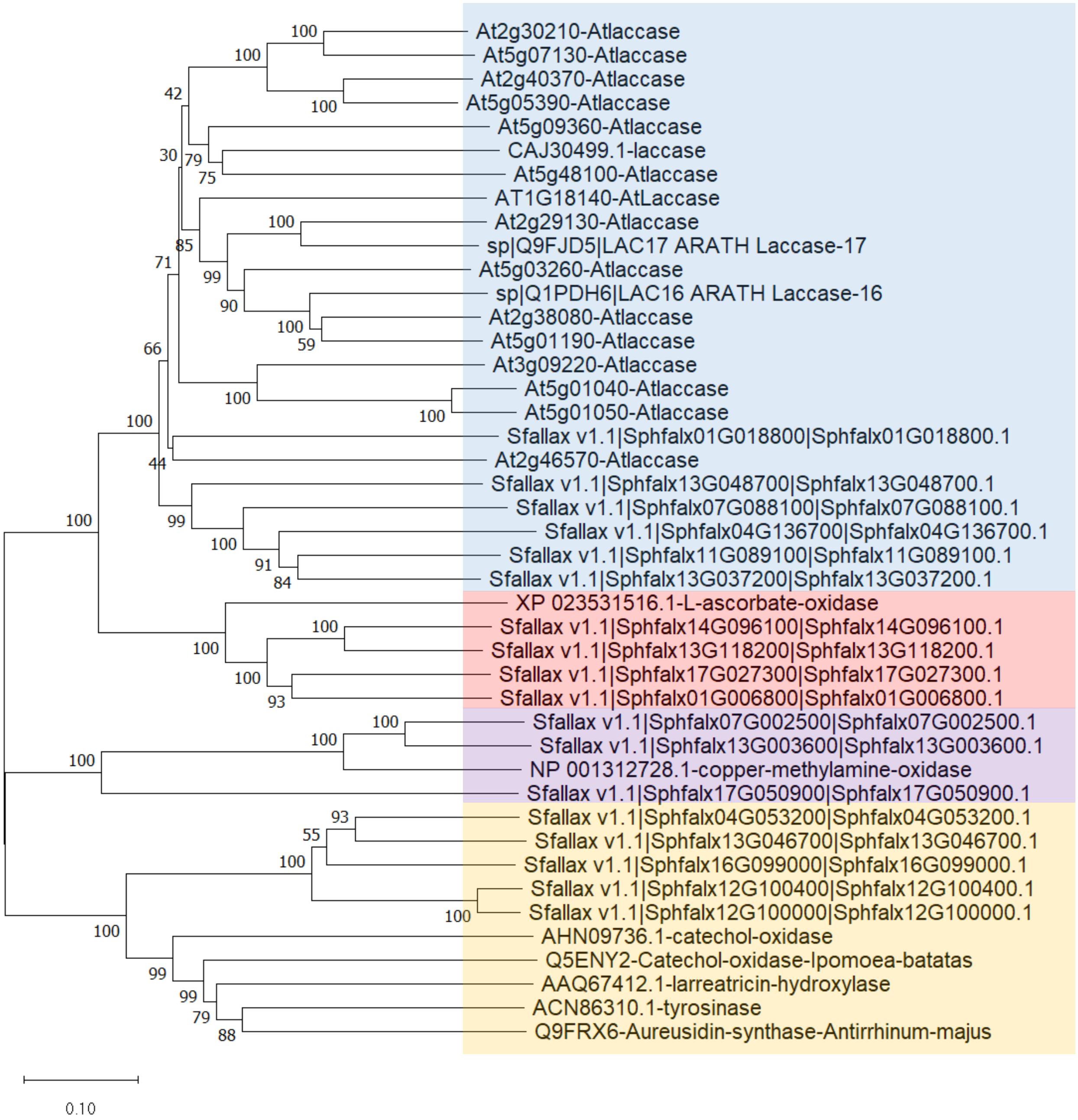

Figure S1 | Phylogenetic analyses of genome-derived copper enzyme genes with characterized copper enzyme genes. *Sphagnum fallax (v1.1).* Laccases highlighted in blue, ascorbate oxidases highlighted in red, undefined copper enzymes highlighted in grey, copper-methylamine-oxidases highlighted in purple, T3 polyphenol oxidases highlighted in orange.

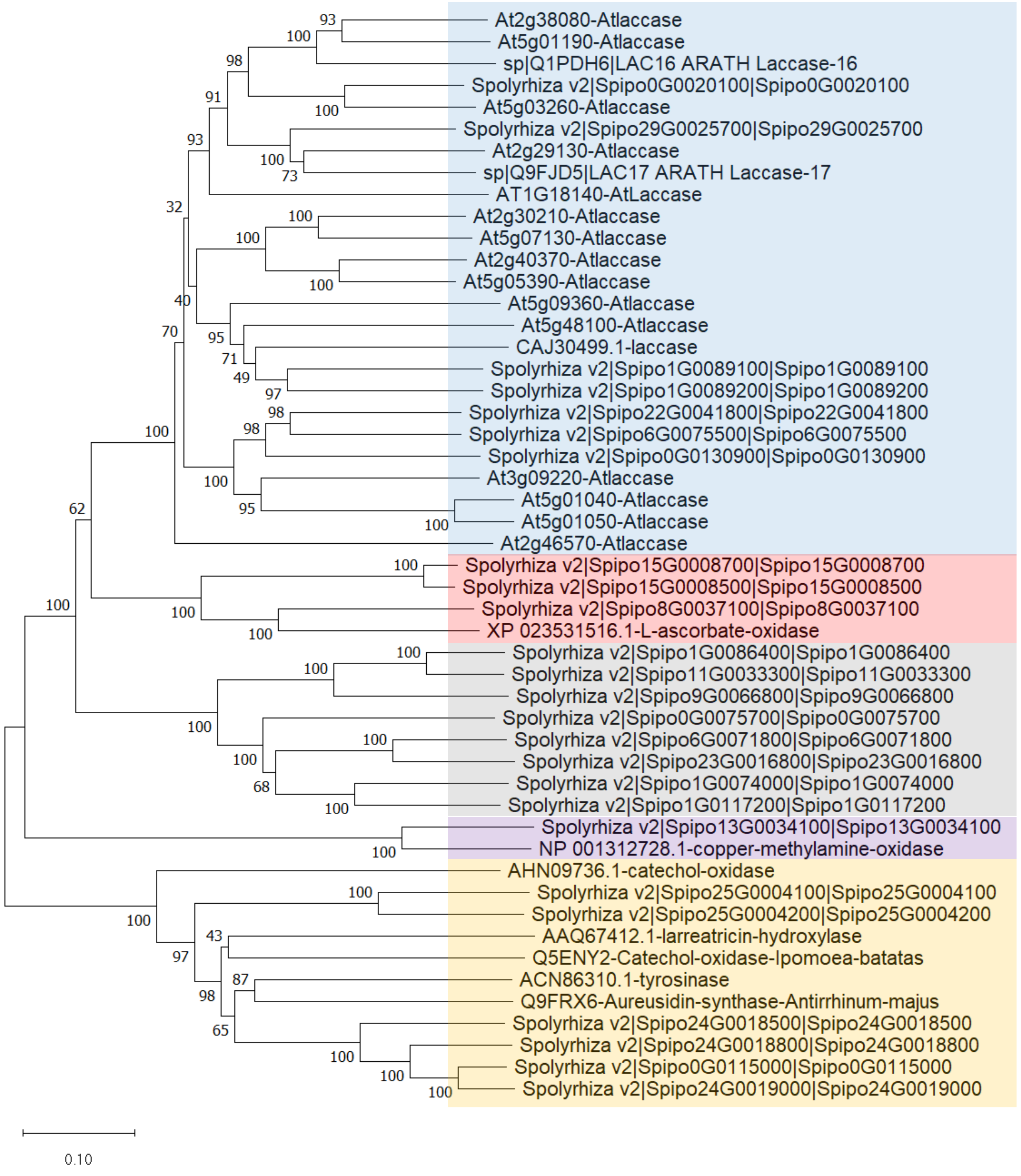

Figure S1 | Phylogenetic analyses of genome-derived copper enzyme genes with characterized copper enzyme genes. *Spirodela polyrhiza (v2).* Laccases highlighted in blue, ascorbate oxidases highlighted in red, undefined copper enzymes highlighted in grey, copper-methylamine-oxidases highlighted in purple, T3 polyphenol oxidases highlighted in orange.

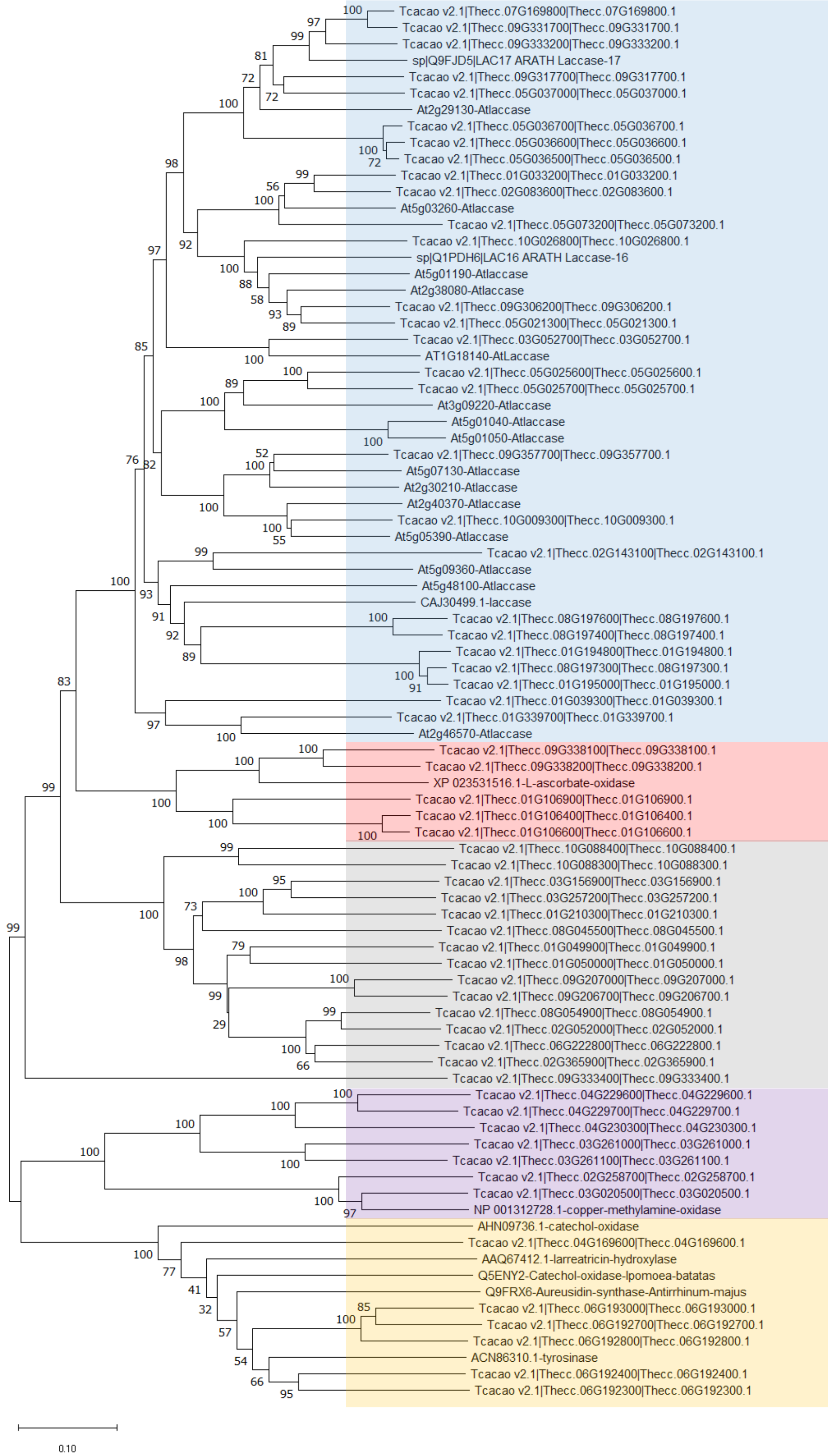

Figure S1 | Phylogenetic analyses of genome-derived copper enzyme genes with characterized copper enzyme genes. *Theobroma cacao (v2.1).* Laccases highlighted in blue, ascorbate oxidases highlighted in red, undefined copper enzymes highlighted in grey, copper-methylamine-oxidases highlighted in purple, T3 polyphenol oxidases highlighted in orange.

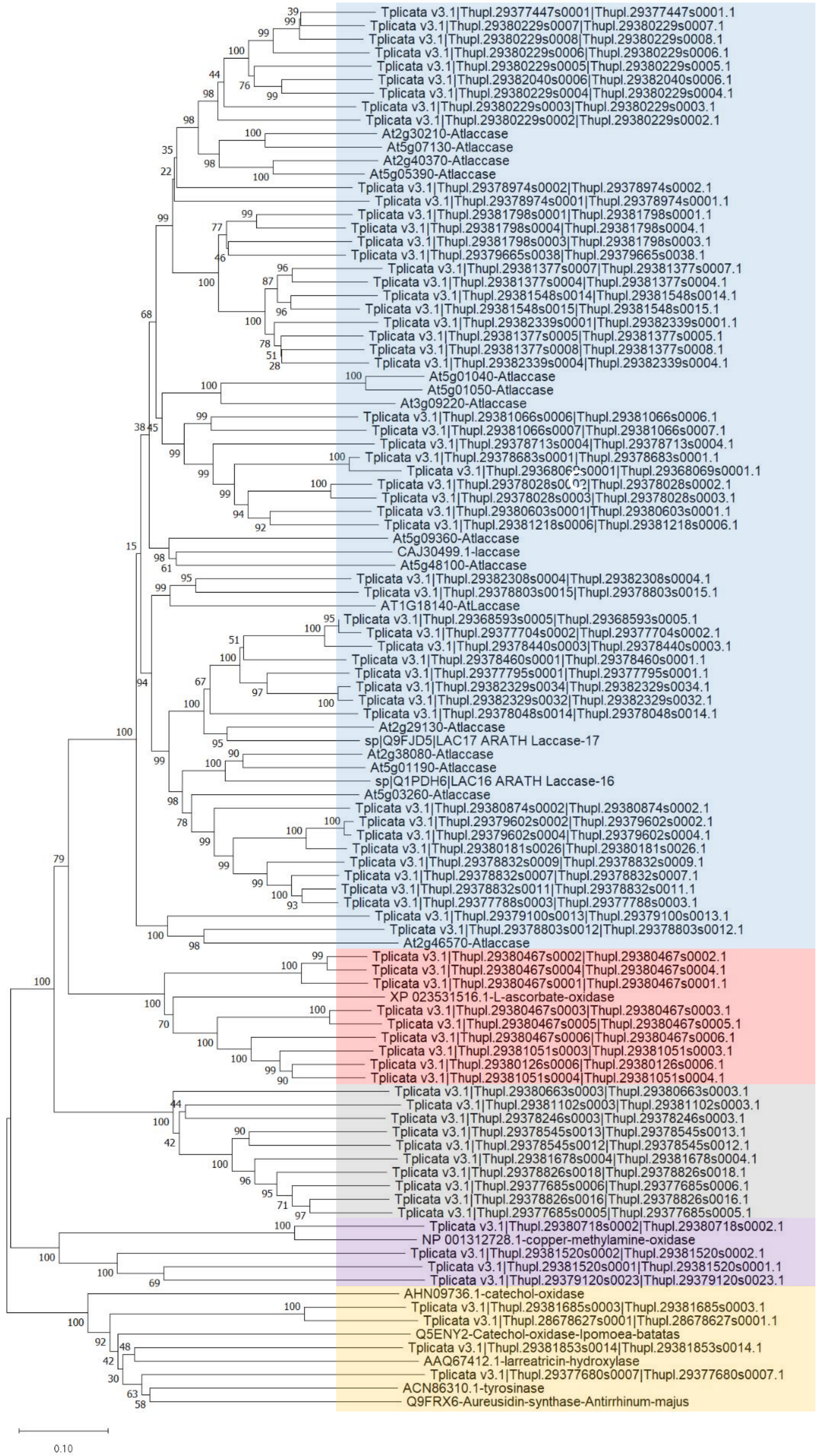

Figure S1 | Phylogenetic analyses of genome-derived copper enzyme genes with characterized copper enzyme genes. *Theobroma cacao (v2.1).* Laccases highlighted in blue, ascorbate oxidases highlighted in red, undefined copper enzymes highlighted in grey, copper-methylamine-oxidases highlighted in purple, T3 polyphenol oxidases highlighted in orange.

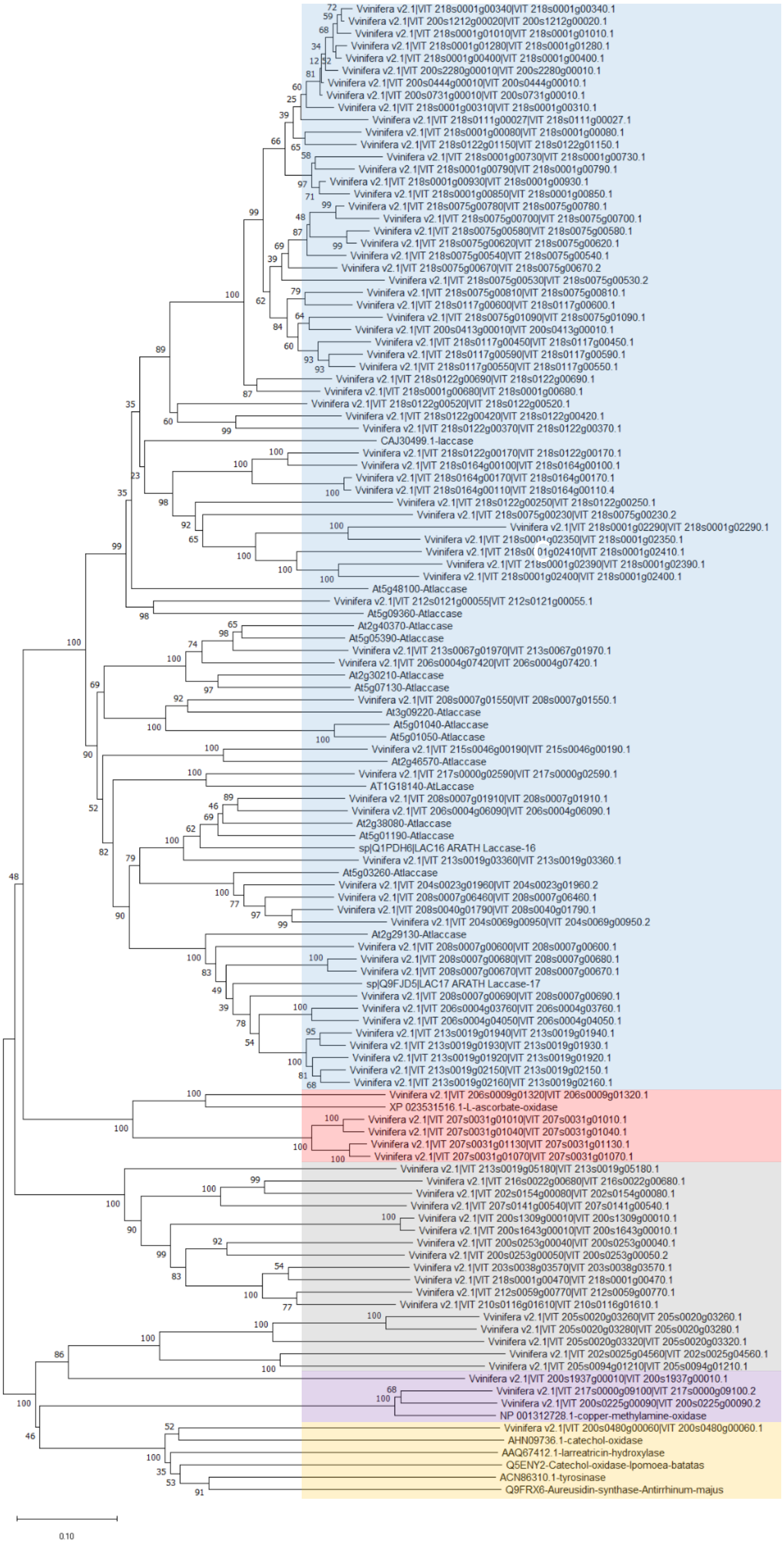

Figure S1 | Phylogenetic analyses of genome-derived copper enzyme genes with characterized copper enzyme genes. *Vitis vinifera (v2.1).* Laccases highlighted in blue, ascorbate oxidases highlighted in red, undefined copper enzymes highlighted in grey, copper-methylamine-oxidases highlighted in purple, T3 polyphenol oxidases highlighted in orange.

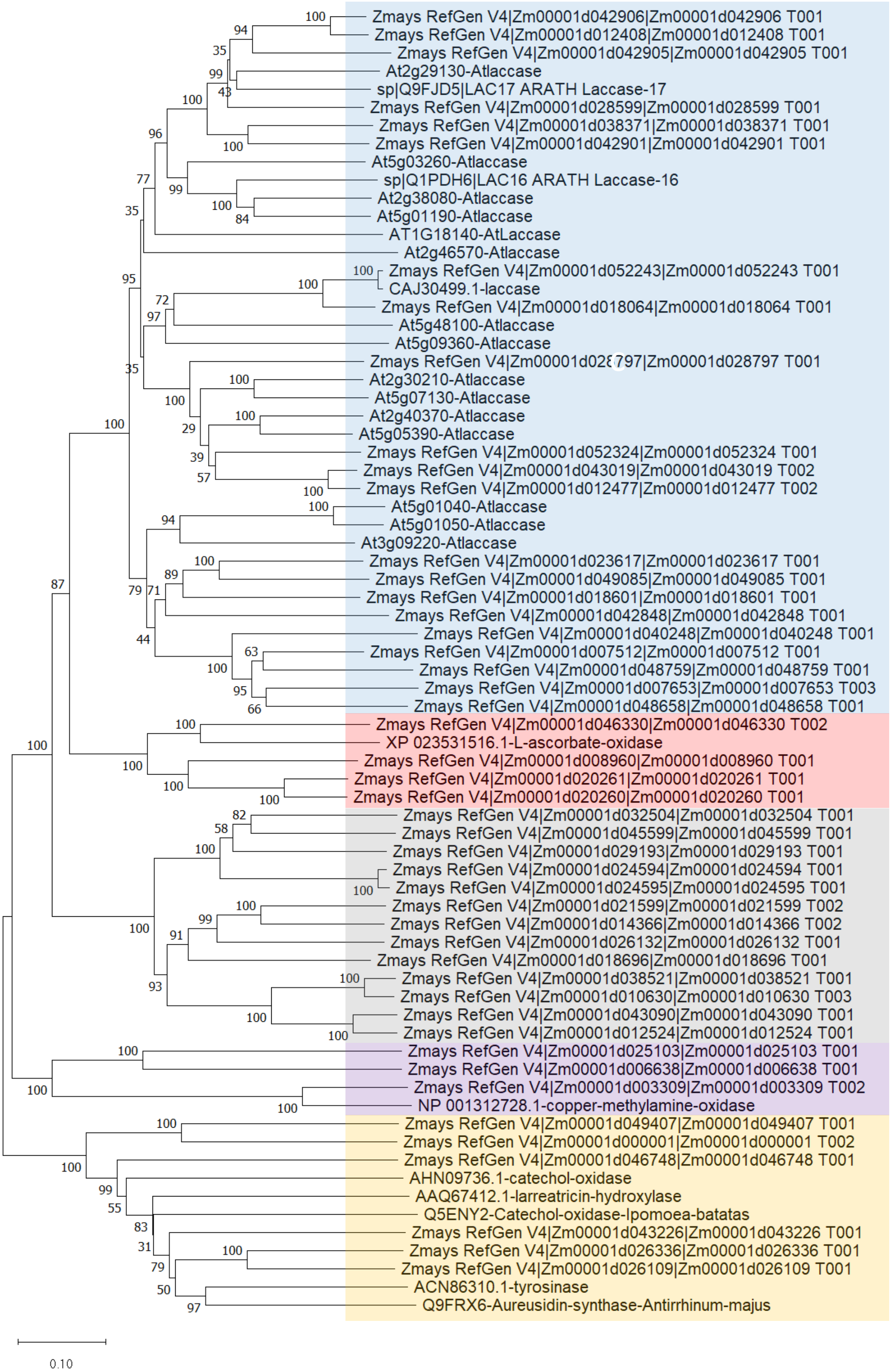

Figure S1 | Phylogenetic analyses of genome-derived copper enzyme genes with characterized copper enzyme genes. *Zea mays (RefGen_V4).* Laccases highlighted in blue, ascorbate oxidases highlighted in red, undefined copper enzymes highlighted in grey, copper-methylamine-oxidases highlighted in purple, T3 polyphenol oxidases highlighted in orange.

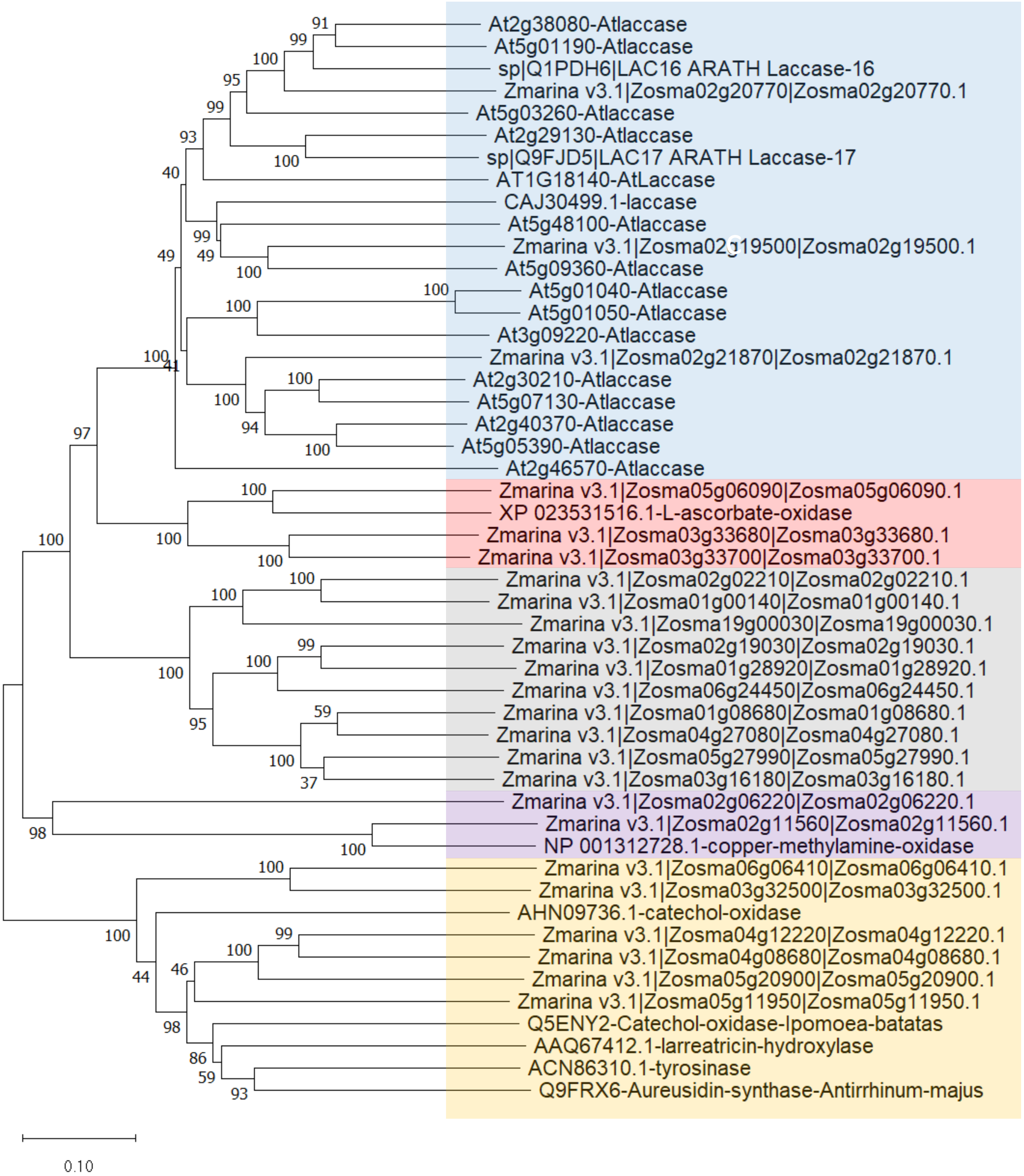

Figure S1 | Phylogenetic analyses of genome-derived copper enzyme genes with characterized copper enzyme genes. *Zostera marina (v3.1).* Laccases highlighted in blue, ascorbate oxidases highlighted in red, undefined copper enzymes highlighted in grey, copper-methylamine-oxidases highlighted in purple, T3 polyphenol oxidases highlighted in orange.
